# Incorporating historic information to further improve power when conducting Bayesian information borrowing in basket trials

**DOI:** 10.1093/biostatistics/kxaf016

**Published:** 2025-06-18

**Authors:** Libby Daniells, Pavel Mozgunov, Helen Barnett, Alun Bedding, Thomas Jaki

**Affiliations:** STOR-i Centre for Doctoral Training, Lancaster University, Fylde College, Lancaster, Lancashire, LA1 4YF, United Kingdom; MRC Biostatistics Unit, University of Cambridge, East Forvie Building, Robinson Way, Cambridge, Cambridgeshire, CB2 0SR, United Kingdom; Department of Mathematics and Statistics, Lancaster University, Fydle College, Lancaster, Lancashire, LA1 4YF, United Kingdom; Roche Products Ltd, Welwyn Garden City, 6 Falcon Way, Shire Park, Welwyn Garden City, Hertfordshire, AL7 1TW, United Kingdom; MRC Biostatistics Unit, University of Cambridge, East Forvie Building, Robinson Way, Cambridge, Cambridgeshire, CB2 0SR, United Kingdom; Faculty of Informatics and Data Science, University of Regensburg, Bajuwarenstraße 4, Regensburg, Bavaria, 93053, Germany

**Keywords:** basket trial, Bayesian modeling, historic information, information borrowing

## Abstract

In basket trials a single therapeutic treatment is tested on several patient populations simultaneously, each of which forming a basket, where patients across all baskets on the trial share a common genetic aberration. These trials allow testing of treatments on small groups of patients, however, limited basket sample sizes can result in inadequate precision and power of estimates. It is well known that Bayesian information borrowing models such as the exchangeability-nonexchangeability (EXNEX) model can be implemented to tackle such a problem, drawing on information from one basket when making inference in another. An alternative approach to improve power of estimates, is to incorporate any historical or external information available. This paper considers models that amalgamate both forms of information borrowing, allowing borrowing between baskets in the ongoing trial whilst also drawing on response data from historical sources, with the aim to further improve treatment effect estimates. We propose several Bayesian information borrowing approaches that incorporate historical information into the model. These methods are data-driven, updating the degree of borrowing based on the level of homogeneity between information sources. A thorough simulation study is presented to draw comparisons between the proposed approaches, whilst also comparing to the standard EXNEX model in which no historical information is utilized. The models are also applied to a real-life trial example to demonstrate their performance in practice. We show that the incorporation of historic data under the novel approaches can lead to a substantial improvement in precision and power of treatment effect estimates when such data is homogeneous to the responses in the ongoing trial. Under some approaches, this came alongside an inflation in type I error rate in cases of heterogeneity. However, the use of a power prior in the EXNEX model is shown to increase power and precision, whilst maintaining similar error rates to the standard EXNEX model.

## 1. INTRODUCTION

Basket trials have been developed as a form of precision medicine in which an experimental treatment is targeted to a specific genetic make-up rather than a disease type as a whole. This acknowledges that not all patients with the same disease will benefit from a treatment in the same way. This may be due to individual variability in genetics alongside other environmental causes. Within a basket trial a single treatment is tested on multiple disease types under one master protocol. Each disease type forms a “basket,” with patients across all baskets harboring the same genetic mutation (Park et al. 2019). Typically such basket trials are implemented in the early stage of the drug development process to assess the efficacy of a treatment on each of the individual baskets. A major advantage of basket trials is the flexibility to test treatments on patients with rare diseases that would not typically warrant their own investigation due to financial and time constraints. However, the small basket sample size that results may cause issues when making inference on treatment effects, particularly in terms of statistical power and precision. Bayesian methodology has been utilized throughout the literature to try and tackle the problem of small sample sizes in basket trials through information borrowing.

Information borrowing refers to the utilization of information from one basket when making inference in another. Most information borrowing approaches utilize an exchangeability assumption, which assumes that the response rates in each of the individual baskets arise from a common distribution (Liu et al. 2022). The rationale behind the exchangeability assumption is the belief that patients with the same genetic mutation demonstrate a similar response to a treatment. The assumption of a common distribution allows the response rates to be modeled through Bayesian hierarchical models, which are a popular approach to borrow information between baskets. They model the response rates as a joint distribution with a common mean and a single parameter that characterizes heterogeneity across the baskets, known as the “borrowing parameter.” The observed response data is used to update the mean and borrowing parameter to obtain posterior parameters in the common distribution and as such, the observed response data for all baskets contributes to the posterior estimates in each of the individual baskets. This is what is referred to as information borrowing. Some prominent information borrowing methods in the literature include the Bayesian hierarchical (BHM, Berry et al. 2013), the exchangeability-nonexchangeability (EXNEX, Neuenschwander et al. 2016) and the modified exchangeability-nonexchangeability models (mEXNEX$ {}_{c} $, Daniells et al. 2023) to name a few. Empirical Bayesian approaches have also been suggested such as Fujikawa’s design (Fujikawa et al. 2020) and power prior approaches first proposed by Ibrahim and Chen (2000). Such empirical methods have the advantage of analytical posteriors and thus are computationally less intensive.

An alternative approach to improve power and precision is to draw on information from historical/external data sources. Historical information may be available for some or all baskets in a trial, where in previous studies the experimental treatment was tested in a similar patient population (Hobbs et al. 2011). An example of this is the MyPathway study (Hainsworth et al. 2018) which investigated the use of Vemurafenib in BRAFV600 mutation cancers, with the VE-BASKET study (Hyman et al. 2015) also examining the same combination. These trials had three baskets in common. Another example is the BELIEVE trial, in which the efficacy and safety of Dabrafenib and Trametinib was investigated in subjects with various BRAF V600E-Mutated cancers. The BELIEVE trial (Shimoi et al. 2024) was a successor of the earlier ROAR (Rare Oncology Agnostic Research) trial (Subbiah et al. 2023) which considered the same treatments and genetic marker, with a thyroid cancer basket in common. Alternatively, historical or external data could be sourced from earlier phases of the study as opposed to separate studies.

Bayesian methods have also been used to borrow information from historic sources, which typically incorporate historic data into the prior distribution used in the ongoing trial. Most methods down-weight historical data depending on heterogeneity to the current data in the ongoing trial. Bennett et al. (2021) outline a comparison of several methods for borrowing from historical control data, these include the power prior (PP, Ibrahim and Chen 2000), modified power prior (MPP, Duan et al. 2006) and commensurate prior (Hobbs et al. 2011).

The methods listed above either borrow within a trial or from historic sources but, to the best of our knowledge, none do both simultaneously. It is well known that information borrowing from *any* source can increase the power and precision of treatment effect estimates, thus incorporating both forms of borrowing is expected to further benefit power. However, this may come with an inflation in the type I error rate when the assumption of exchangeability between baskets is broken. This occurs when there is heterogeneity between baskets’ observed responses. Type I error inflation could also be a result of heterogeneity between current and historic data sources (Kopp-Schneider et al. 2020). To add to this, one must be wary of concerns of bias in historical sources which may arise due to differences in patient populations over time and differing trial conditions (van Rosmalen et al. 2018). We therefore consider it desirable to prioritize and put more weight on borrowing within an ongoing trial than from historic baskets in order to minimize these potential biases.

In this paper, we propose several Bayesian approaches for borrowing between both current baskets and historic sources under one framework. Note that current baskets refers to baskets that form the ongoing study and historic baskets refers to baskets from historical/external data sources. The proposed approaches include: an EXNEX model where a baskets’ probability of exchangeability is determined by the homogeneity between historic baskets; an EXNEX model with a power prior placed on the NEX component; a multi-level mixture model consisting of two EXNEX models (one with historic information and one without); an EXNEX model with pooled historic and current data. Approaches are explored through a simulation study which focuses on binary response data. The design parameters implemented in the simulation study are motivated by the MyPathway and VE-BASKET trials. These design parameters are used to make the simulation as close to a real trial application as possible, this will demonstrate what the potential operating characteristics of the proposed designs would be in practice. Results display the clear benefit of incorporating the historic information alongside borrowing between current baskets in terms of power gain compared to analyzing current data independent of historic data. The results also show a trade-off of this power gain with a slight inflation of error rates, with some approaches demonstrating more inflation than others. These conclusions are supported by our findings from applying the models to the BELIEVE and ROAR trial data.

## 2. MOTIVATING EXAMPLE

The MyPathway trial (Hainsworth et al. 2018) ran from 2014 to 2023 and consisted of multiple non-randomised basket trials under one master protocol. One branch of this trial applied the drug Vemurafenib in patients with solid tumors harboring the BRAFV600 mutation. Patients with the BRAFV600E-mutated cancers were enrolled across the following baskets: non-small-cell lung cancer (NSCLC), ovarian cancer, colorectal cancer, anaplastic thyroid cancer and head/neck (larynx) cancer. A Simon’s two-stage design (Simon 1989) for 10% type I error rate and 80% power was used to determine planned sample sizes, with the null and target responses set dependent on the classification of treatment resistance of each tumor type: treatment resistant cancers (eg NSCLC) have a null and target response rate of 5% and 20% respectively, resulting in a sample size of 21 patients per basket; non-treatment resistant cancers (eg colorectal or ovarian cancer) have a null and target response rate of 10% and 25% respectively, resulting in a sample size of 34 patients per basket.

The combination of Vemurafenib on patients with BRAFV600 mutation cancers was also studied in the earlier VE-BASKET trial (Hyman et al. 2015) which ran from 2012 to 2014. Both the MyPathway and VE-BASKET trials shared three baskets is common: NSCLC, colorectal cancer and anaplastic thyroid cancer. In the VE-BASKET trial a smaller sample size of 13 patients per basket was planned via a Simon’s two-stage design based on 10% type I error rate and 80% power with a null and target response rate of 15% and 45%. It appears that both trials were conducted distinctly, with information from the VE-BASKET trial not incorporated into the design or analysis of the MyPathway study. One could argue that the information from the three baskets of common interest could have been utilized in the MyPathway study to inform analysis in some meaningful way. This provides motivation for a trial design that can incorporate borrowing from both current and historic baskets.

## 3. METHODS

### 3.1. Setting

This paper focuses on non-randomised basket trials with a single treatment arm and binary endpoint, where a patient either responds to the treatment or does not. Let there be at least one historic basket trial investigating the same treatment on the same genetic aberration with some baskets in common with the current trial.

Consider a basket trial consisting of $ K $ baskets with historic information available for $ K^{*}\in 1, \dots, K $ of them. For current basket $ k $, there are a total of $ H_{k} $ historic sources of data, where in each past study patients of the same disease type as in basket $ k $ received the experimental treatment under investigation. Assume the first $ 1,2, \ldots, K^{*} $ current baskets have historic information and that current baskets $ K^{*}+1, \ldots, K $ do not. Responses in a current basket $ k $ are denoted by $ Y_{k} $ which follows a Binomial distribution: $ Y_{k}\sim\mathrm{Binomial}(n_{k},p_{k}) $ with sample size $ n_{k} $ and the unknown response rate, $ p_{k} $. Given that current basket $ k $ has historic data from $ H_{k} $ previous studies, denote the basket from historic study $ j $ ($ j\in\{1, \ldots, H_{k}\} $) associated with current basket $ k $ as $ k^{*^{(j)}} $, the responses in basket $ k^{*^{(j)}} $ are distributed $ Y_{k^{*^{(j)}}}\sim\mathrm{Binomial}(n_{k^{*^{(j)}}},p_{k^{*^{(j)}}}) $ with sample size $ n_{k^{*^{(j)}}} $ and response rate $ p_{k^{*^{(j)}}} $. Should only one historic study exist, the superscript $ (j) $ is removed and historic data is simply denoted $ k^{*} $ for basket $ k $.

Denote the null response rate in the current trial as $ q_{0} $. The objective is to test the family of hypotheses: $ H_{0}\;:\; p_{k}\leq q_{0} $ vs. $ H_{1}\;:\; p_{k} > q_{0} $ ($ k\,=\,1, \ldots, K $), which is done under a Bayesian framework. If historic data, $ D_{h} $, is available, having observed response data $ D $ for the current trial, the treatment is deemed effective in basket $ k $ if $ \mathbb{P}(p_{k} > q_{0}|D, D_{h}) \gt\Delta_{k} $. The decision criteria $ \Delta_{k} $ is typically determined through calibration in order to control some metric to a nominal level, which is often the basket-wise type I error rate.

### 3.2. EXNEX model

The exchangeability-nonexchangeability (EXNEX, Neuenschwander et al. 2016) model is an approach to borrow information between baskets. This model provides adaptive and flexible borrowing by relaxing the exchangeability assumption that the response rates in *all* baskets share a common distribution. As such, the response rates from only a subset of baskets share a common distribution, whilst the response rate in other baskets are modeled by independent distributions. This accounts for some heterogeneity between baskets. The EXNEX model as proposed by Neuenschwander et al. (2016) does not take into account any historic or external data and considers current baskets only. The EXNEX model consists of a mixture of two components:

1. Exchangeable (EX) component: Baskets are considered exchangeable within this component and therefore, information borrowing is conducted between them using a Bayesian hierarchical model (BHM, Berry et al. 2013). Basket $ k $ is assigned to the EX component with prior probability $ \pi_{k} $. The EX component is denoted as $ M_{1k} $, with a BHM placed on the logit-transformed parameter $ \theta_{1k} $.
2. Nonexchangeable (NEX) component: Baskets are analyzed independently in this component and are considered nonexchangeable with other baskets. As such, basket specific priors are placed on the response rate and no information drawn from the other baskets. Baskets are assigned to the NEX component with prior probability $ 1-\pi_{k} $. The NEX component is denoted as $ M_{2k} $, with the prior placed on the logit-transformed parameter $ \theta_{2k} $.
  (3.1) \begin{align*} Y_{k}\sim\mathrm{Binomial}(n_{k},p_{k}),\quad k\,=\,1, \ldots, K\end{align*}
   
  (3.2) \begin{align*} p_{k}=\delta_{k}M_{1k}+(1-\delta_{k})M_{2k},\end{align*}
   
  (3.3) \begin{align*} \delta_{k}\sim\mathrm{Bernoulli}(\pi_{k}),\end{align*}
   
  (3.4) \begin{align*} \theta_{1k}=\mathrm{logit}(M_{1k})\sim\mathrm{N}(\mu, \sigma^{2}),\mathrm{(EX)}\end{align*}
   
  (3.5) \begin{align*} \mu\sim\mathrm{N}(m_{\mu},\nu_{\mu}),\end{align*}
   
  (3.6) \begin{align*} \sigma\sim g(\cdot),\end{align*}
   
  (3.7) \begin{align*} \theta_{2k}=\mathrm{logit}(M_{2k})\sim\mathrm{N}(m_{k},\nu_{k}).\mathrm{(NEX)}\end{align*}

As the EX component is a BHM, response rate estimates within this component are shrunk toward the common mean, $ \mu $, with the degree of shrinkage controlled by $ \sigma^{2} $. As $ \sigma^{2} $ tends to 0, borrowing becomes akin to complete pooling of results, however, as it tends to infinity, stratified analysis of each basket is conducted. Typically it is suggested that a slightly informative prior is placed on $ \mu $ (Dayimu et al. 2024), for instance by setting $ m_{\mu} $ in (3.5) to $ \mathrm{logit}(q_{0}) $ with a large variance $ \nu_{\mu} $. Several arguments have been made around the choice of prior, $ g(\cdot) $, on $ \sigma $, with a Half-Normal, Inverse-Gamma or Half-Cauchy density among those suggested. Gelman (2006) argued that the original suggestion of an Inverse-Gamma prior by Berry et al. (2013) had poor behavior when $ \sigma^{2} $ is too close to 0, thus suggested a Half-Cauchy prior instead. Neuenschwander et al. (2016) implemented a Half-Normal prior with scale parameter equal to 1 as this led to conservative borrowing, limiting the potential for type I error inflation when responses are heterogeneous. A half-normal prior was also suggested by Zheng and Wason(2022), Dayimu et al. (2024) and Cunanan et al. (2019). Values for the $ m_{k} $ and $ \nu_{k} $ parameters in the NEX component (3.7) were suggested by Neuenschwander et al. (2016) as: $ m_{k}=\mathrm{logit}(\rho_{k}) $ and $ \nu_{k}=1/\rho_{k}+1/(1-\rho_{k}) $, where $ \rho_{k} $ is a plausible guess for $ p_{k} $. This plausible guess could be based on the observed response of the treatment under investigation in other populations or as a value somewhere between the null and target response rate (as supported by a sensitivity analysis presented in the Supplementary Materials).

The $ \delta_{k} $ mixtures are binary variables with prior probabilities of success denoted $ \pi_{k} $. These prior mixture weights, $ \pi_{k} $, reflect the probability of exchangeability between basket $ k $ and the other baskets on the trial. Often these values are set a priori at $ \pi_{k}=0.5 $ for all $ k $ baskets as little to no prior knowledge of the probability of exchangeability is known. As data is observed, it is used to update the prior values to obtain posterior probabilities of exchangeability. The posterior probabilities are higher when a basket has a homogenous response, thus increasing its probability of being in the EX component of the EXNEX model. Whereas, when a basket has a heterogeneous response rate, more weight is placed on the NEX component and the posterior of $ \pi_{k} $ reduced. Alternatively a Dirichlet prior could be placed on $ \pi_{k} $ but as stated by Neuenschwander et al. (2016) this has little to no effect on operating characteristics.

### 3.3. EXNEX with a power prior in the NEX component

Considering the EXNEX with a power prior in the NEX component (EXNEX) model, baskets with a homogeneous response rate are assigned to the EX component and information is borrowed between them using a hierarchical model. Power improvement is expected in these baskets due to this borrowing, however, baskets assigned to the NEX component are analyzed independently and thus still suffer from the lack of statistical power and precision previously discussed due to their limited sample size. Therefore, it is likely that baskets in the NEX component will benefit more substantially from borrowing from historical data than those already implementing information borrowing in the EX component.

To incorporate historical information into the NEX component, a power prior approach is used. A power prior (PP) was first introduced by Ibrahim and Chen(2000) in order to incorporate historical information into a current trial. This is achieved by raising the likelihood of the historical data for each of the $ j\,=\,1, \ldots, H_{k} $ studies to a fixed power, $ \alpha_{j} $. The power prior for basket $ k $, having observed historic response data $ y_{k^{*{(j)}}} $ for each of the $ j\,=\,1, \ldots, H_{k} $ historic studies, has the following form: $ \pi(p_{k}|\boldsymbol{y_{k^{*}}},\boldsymbol{\alpha})\propto\prod^{H_{k}}_{j\,=\,1}L(p_{k}|y_{k^{*^{(j)}}})^{\alpha_{j}}\times\pi_{0}(p_{k}) $, where $ L() $ denotes the likelihood function and $ \pi_{0}(p_{k}) $ is an initial vague prior on $ p_{k} $, defined before looking at any historic data and $ \boldsymbol{y_{k^{*}}} $ is the set of historic responses for basket $ k $, whilst $ \boldsymbol{\alpha} $ is the set of $ \alpha_{j} $ power values associated with studies $ j\,=\,1, \ldots, H_{k} $. The power values, $ \alpha_{j} $, are typically bound between 0 and 1 and reflects the expected homogeneity between historic and current data. $ \alpha_{j} $ controls the amount of borrowing as it weights the contribution of the historic data in the posterior parameters: values closer to 0 will result in no borrowing from the historic information, whereas values closer to 1 will result in the prior corresponding to the posterior of the historic data with no down-weighting. These $ \alpha_{j} $ parameters are trial specific, allowing some historical studies to carry more weight than others.

For the EXppNEX approach, the NEX component in (3.7) is replaced with a mixture prior consisting of an informative part ($ P_{1k} $) based on the historic information available, and non-informative part ($ P_{0k} $). The mixture is dependent on the presence of historical data: $ M_{2k}=\mathbb{I}_{k}P_{1k}+(1-\mathbb{I}_{k})P_{0k}, $ where $ \mathbb{I}_{k}=1 $ if historic data $ y_{k^{*^{(j)}}} $ exists for basket $ k $ for some $ j\geq 1 $, and 0 should no historic information be available for basket $ k $. Now $ P_{1k} $ takes the form of the PP, and as such, given an initial $ \pi_{0}(p_{k})= $ Beta($ a_{k},b_{k} $) prior on $ p_{k} $ for current basket $ k $:


(3.8) \begin{align*} P_{1k}\sim\mathrm{Beta}\left(a_{k}+\sum^{H_{k}}_{j=1}\alpha_{j}y_{k^{*^{(j)}}},b _{k}+\sum^{H_{k}}_{j=1}\alpha_{j}(n_{k^{*^{(j)}}}-y_{k^{*^{(j)}}})\right).\end{align*}

When available, the PP incorporates the historic information into the model but does not induce borrowing between other baskets on the current trial within the PP itself. To allow for unavailable historic information, $ P_{0k} $ is an uninformative normal distribution placed on the logit-transformed parameter as in (3.7): $ \theta_{2k}=\mathrm{logit}(P_{0k})\sim N(m_{k},\nu_{k}). $

### 3.4. A multi-level mixture model

The proposed EXppNEX approach only incorporates historic information in the NEX component of the EXNEX model and thus baskets assigned to the EX component do not benefit from the historic data. Should all baskets on the trial demonstrate very similar or identical response rates, all baskets are exchangeable and as such are all assigned to the EX component (3.4) of the EXNEX model. This means that all available historic information is completely disregarded, therefore any potential power gain is wasted. This motivates the need to also incorporate historical information into the EX component to some degree.

One could argue for including historic baskets as distinct baskets in the current trial when conducting analysis, treating them identically to baskets in the ongoing study. When applying the EXNEX model to such a scenario, the historic baskets could be included in the EX’s Bayesian hierarchical model, thus inducing borrowing directly from the historic information. However, this ignores the fact that historic baskets correspond to specific baskets in the current trial, inducing the same level of borrowing between a basket and its’ own historic information as it does between this historic basket and other non-corresponding baskets on the trial. It also puts equal importance of borrowing from historic and current baskets. On the other hand, due to the exchangeability assumption it is assumed a priori that *all* baskets are exchangeable due to the shared genetic component, thus a basket borrowing from its’ own historic information should be just as acceptable as borrowing from another baskets’ historic data. The mixture weights, $ \pi_{k} $, within the EXNEX model should update to assign any heterogeneous historic information into the NEX component in order to restrict borrowing and limit error inflation. However, it is known that the EXNEX model is not sensitive enough to the presence of heterogeneity and thus weights are set too high in this case, inducing too much borrowing resulting in error inflation. This approach would be seen as a more “extreme” method for borrowing from historic information, which in cases of homogeneity will give substantial improvements in power, but as stated, will likely observe unacceptable error inflation in cases of heterogeneity.

We take this concept of an EXNEX model consisting of all current and historic information and extend it to better handle cases of heterogeneity between current and historic data sources. This is achieved by taking a mixture of such an EXNEX model with a standard EXNEX model that disregards historic information. The mixture weights between these two models will reflect the degree of conflict between the current and historic data. In cases of homogeneity between a current basket and the historic information, mixture weights will shift and put a higher weight on the EXNEX model consisting of historic data, and in cases of heterogeneity, put more weight on the standard EXNEX model, which disregards the heterogeneous historic data. The mixture weights can also be adjusted to put a heavier emphasis on borrowing between baskets on the current trial.

First, to re-emphasize, all baskets current and historic are modeled in the multi-level mixture (MLMixture) model: $ Y_{i}\sim\mathrm{Binomial}(n_{i},p_{i}) i = 1, \ldots, K,1^{*^{(1)}},\ldots, 1^{*^{(H_{1})}},\ldots, K^{*^{(1)}},\ldots, K^{*^{(H_{K^{*}})}} $, however, interest lies only in the estimation of the response rates in the current baskets $ 1, \ldots, K $. Note the subscript has been altered to $ i $ as opposed to $ k $ in order to distinguish that all current and historic baskets are modeled within this MLMixture model, as historic baskets are treated akin to current baskets in the ongoing trial. We also define an indicator $ \psi_{i} $ that takes value 1 if basket if $ i\,=\,1 $ is a historic basket or 0 otherwise.

The MLMixture model comprises of two EXNEX models, the first of which is denoted EXNEX$ {}_{\mathrm{all},i} $, which models all current and historic baskets through an EXNEX model, treating historic in the same way as current. The EX component, M$ {}_{\mathrm{all},1i} $, therefore will consist of a subset of current and historic baskets within which information is shared through a BHM. In EXNEX$ {}_{\mathrm{all},i} $, the NEX component, M$ {}_{\mathrm{all},2i} $ is an informative prior based on the observed historic data. If basket $ i $ is historic and therefore $ \psi_{i}=1 $, this prior is just an uninformative Beta($ a_{i},b_{i} $) prior. As such, the EXNEX$ {}_{\mathrm{all},i} $ component has the following form:


\begin{align*}& \mathrm{EXNEX}_{\mathrm{all},i}=\delta_{i, \mathrm{all}}M_{\mathrm{all},1i}+(1-\delta_{i, \mathrm{all}})M_{\mathrm{all},2i},\\& \delta_{i, \mathrm{all}}\sim\mathrm{Bernoulli}(\pi_{\mathrm{all},i}),\\& \theta_{\mathrm{all},1i}=\mathrm{logit}(M_{\mathrm{all},1i})\sim\mathrm{N}(\mu_{\mathrm{all}},\sigma^{2}_{\mathrm{all}}),\\& \mu_{\mathrm{all}}\sim\mathrm{N}(m_{\mu_{\mathrm{all}}},\nu_{\mu_{\text {all}}}),\\& \sigma_{\mathrm{all}}\sim g(\cdot),\\& M_{\mathrm{all},2i}\sim\mathrm{Beta}\left(a_{i}+(1-\psi_{i})\sum^{H_ {i}}_{t=1}y_{i^{*^{(t)}}},b_{i}+(1-\psi_{i})\sum^{H_{i}}_{t=1}(n_{i^{*^{(t)}}} -y_{i^{*^{(t)}}})\right),\end{align*}

where the mixture weights, $ \delta_{i, \mathrm{all}} $, are updated by the data to reflect the degree of homogeneity between the current and historic baskets. These mixture weights are sampled from a Bernoulli distribution, with the posterior mean close to 1 when basket $ i $ has a homogeneous response rate to other current and historic baskets, thereby increasing the degree of borrowing by placing a greater weight on the exchangeability component. As $ \pi_{\mathrm{all},i} $ move toward 0, more weight is placed on the nonexchangeability component, which borrows from the historic information but does not borrow information from current baskets.

The second EXNEX model, denoted EXNEX$ {}_{\mathrm{curr},i} $, does not induce any borrowing from historic data. This model has a very similar form to the EXNEX$ {}_{\mathrm{all},i} $ model, however, historic baskets are forced into the NEX component, M$ {}_{\mathrm{curr},2i} $, meaning that information is not borrowed from historic data in the EX component. The M$ {}_{\mathrm{curr},2i} $ component is simply an uninformative Beta($ a_{i},b_{i} $) prior, therefore, also ignores historic data. The EXNEX$ {}_{\mathrm{curr},i} $ model has the following form:


\begin{align*}& \mathrm{EXNEX}_{\mathrm{curr},i}=\delta_{i, \mathrm{curr}}M_{\mathrm{curr},1i}+(1-\delta_{i, \mathrm{curr}})M_{\mathrm{curr},2i},\\& \delta_{i, \mathrm{curr}}\sim\mathrm{Bernoulli}((1-\psi)\pi_{\mathrm{curr},i}),\\& \theta_{\mathrm{curr},1i}=\mathrm{logit}(M_{\mathrm{curr},1i})\sim\text {N}(\mu_{\mathrm{curr}},\sigma^{2}_{\mathrm{curr}}),\\& \mu_{\mathrm{curr}}\sim\mathrm{N}(m_{\mu_{\mathrm{curr}}},\nu_{\mu_{\mathrm{curr}}}),\\& \sigma_{\mathrm{curr}}\sim f(\cdot),\\& M_{\mathrm{curr},2i}\sim\mathrm{Beta}(a_{i},b_{i}),\end{align*}

where mixture weights, $ \delta_{i, \mathrm{curr}} $ are set to 0 for all historic baskets $ i=1^{*^{(1)}},\ldots, 1^{*^{(H_{1})}},\ldots, K^{*^{(1)}},\ldots, K^{*^{(H_{K^{*}})}} $. For current baskets $ 1, \ldots, K $, these mixture weights now only reflect the level of homogeneity between itself and all other current baskets.

To fit the MLMixture model, both EXNEX$ {}_{\mathrm{all},i} $ and EXNEX$ {}_{\mathrm{curr},i} $ are fit distinctly. The posterior for basket $ k $ is then a mixture of the posteriors obtained under both models:


\begin{align*}& p_{k}=\lambda_{k}\mathrm{EXNEX}_{\mathrm{all},k}+(1-\lambda_{k})\mathrm{EXNEX}_{\mathrm{curr},k},\\& \lambda_{k}\sim\mathrm{Bernoulli}(\pi_{\lambda, k}),\end{align*}

where $ \lambda_{k} $ reflects the degree of homogeneity between a current basket $ k $ and its own historic baskets’ $ k^{*^{(j)}} $, as well as, the homogeneity to other baskets’ historic data. A prior distribution is placed on the $ \lambda_{k} $ parameter with prior probabilities $ \pi_{\lambda, k} $, where values close to 1 can induce a higher level of borrowing from historic baskets, whilst values close to 0 analyze current baskets as independent from any historic data. The prior probabilities are updated by the observed response data to obtain posterior estimates. Note, the $ \lambda_{k} $ value will not measure the degree of homogeneity between current baskets as there is potential to borrow between these baskets in both sides of the mixture.

This model provides flexibility, allowing baskets with historic sources to borrow between both current baskets and all historic data, whilst letting baskets without historic information to also gain from the historic information of other exchangeable baskets. Similarly, should data be heterogeneous, the model has the option of analyzing as completely independent. A downside of this approach is its computational intensity as the extra layers of mixture and increased number of variables increases the model complexity. The computational time will also increase as both $ K $ and $ K^{*} $ increase. A further discussion on computation time is presented in Appendix S6 of the Supplementary Materials.

## 4. SIMULATION STUDY

Two approaches for incorporating historic information have been outlined, in this section we aim to explore the operating characteristics of each model to compare performance. Performance of approaches are assessed using extensive simulation studies. The design parameters from the MyPathway and VE-BASKET trials form the basis of these simulation studies. Thus, as in the MyPathway trial, the simulation study consists of $ K\,=\,5 $ current baskets with historical information for the first $ K^{*}=3 $, also assume that $ H_{k}=1 $ for $ k\,=\,1,2,3 $ so that when historic information is available, there is only a single source of historic data, thus any superscripts $ (j) $ may be dropped for notation sake. Sample sizes are fixed and equal across the current baskets $ k\,=\,1, \ldots, K $ at $ n_{k}=34 $, with a null and target response rate of $ q_{0}=0.1 $ and $ q_{1}=0.25 $ respectively. Sample sizes for each of the historic baskets are fixed at the planned sample size of the VE-BASKET study at $ n_{k^{*}}=13 $, whilst a simulation study that uses the actual observed unequal sample sizes and number of responses for the historic data is provided in the Supplementary Materials. As in the MyPathway study, the target/nominal type I error rate and power are 10% and 80% respectively.

Within the simulation study, responses in the current baskets are randomly sampled based on a true response rate, whilst historic data is fixed. This mimics a trial setting where simulation studies are conducted prior the current trial, at which time the historic information has already been observed. A total of 8 true response rate data scenarios were considered for the current data and are presented in the left-hand side of Table 1. Scenario 1 represents the global null in which the treatment is ineffective in all baskets, whereas, scenario 6 is the global alternative under which all are effective. Scenarios 2-5 cover partial nulls with an increasing number of baskets where the treatment is effective. Scenarios 7 and 8 both consider cases where one of the baskets without historic information is effective and varied the effectiveness in baskets with historic information. Each of these 8 data scenarios are split into four sub-cases consisting of four different historic data settings, resulting in a total of 32 simulation scenarios. As the null response rate is 0.1, observing a single positive response in a historic basket out of the 13 patients corresponds to an ineffective treatment. In contrast, observing three responses corresponds to an effective treatment. The right-hand side of Table 1 shows the four different historic data sub-cases (a) to (d) that are considered, which vary the number of historic baskets that have a positive response to treatment, where (a) corresponds to the treatment being ineffective in all historic baskets and (d) corresponds to the treatment being effective in all historic baskets. Sub-cases (b) and (c) is where the treatment is effective in some historic baskets and ineffective in others.

**Table 1.** True response rate data scenarios considered in the simulation study and historic data sub-cases considered for each of the eight data scenarios.

Scenario	$ p_{1} $	$ p_{2} $	$ p_{3} $	$ p_{4} $	$ p_{5} $	Sub-case	$ y_{1^{*}} $	$ y_{2^{*}} $	$ y_{3^{*}} $
1	0.10	0.10	0.10	0.10	0.10	(a)	1	1	1
2	0.25	0.10	0.10	0.10	0.10	(b)	3	1	1
3	0.25	0.25	0.10	0.10	0.10	(c)	3	3	1
4	0.25	0.25	0.25	0.10	0.10	(d)	3	3	3
5	0.25	0.25	0.25	0.25	0.10				
6	0.25	0.25	0.25	0.25	0.25				
7	0.10	0.10	0.10	0.25	0.10				
8	0.25	0.10	0.10	0.25	0.10				

Efficacy is determined using posterior distributions, so having observed current data $ D $ and historic data $ D_{h} $, basket $ k $ is deemed sensitive to the treatment if $ \mathbb{P}(p_{k}\geq 0.1|D, D_{h})\geq\Delta_{k} $. Traditionally this efficacy cut-off $ \Delta_{k} $ would be calibrated under the global null scenario to control the basket-wise type I error. However, this simulation study implements the Robust Calibration Procedure (RCaP, Daniells et al. 2025) in order to achieve an average basket-wise type I error rate of 10% across a number of scenarios as opposed to under just the null. RCaP is taken across all 8 scenarios presented in Table 1 to produce cut-off values $ \Delta_{k} $. Note that $ \Delta_{k} $ values are calibrated separately for each of the four sub-cases and for each of the six approaches. The calibrated $ \Delta_{k} $ values are presented alongside the RCaP outline in the Supplementary Materials.

For each scenario several operating characteristics were computed. This text focuses on the percentage of simulated data sets where the null hypothesis was rejected (% Reject). If the true response rate is null, this is the basket-wise type I error rate, else it is the power. The family-wise error rates (FWER), the mean point estimates for the response rates and their standard deviations are provided in the Supplementary Materials. A total of 5,000 simulations were run for each scenario using the “rjags” package v 4.15 (Plummer 2023) with R v 4.1.2 (R Core Team 2021). The MCMC was conducted with 100,000 iterations for each simulation.

### 4.1. Competing approach

An alternative approach to historic information borrowing, is one in which the historic data is used to define the probabilities of exchangeability prior to observing data from the current baskets. These prior probabilities are then used as mixture weights in (3.3) in the EXNEX model to analyze data from the current baskets. Therefore, within this approach, denoted mEXNEX$ {}_{\mathrm{hist}} $, historical information is not borrowed from directly and data is not used in the analysis model itself beyond updating the mixture weights.

This can be viewed as a version of the modified exchangeability-nonexchangeability (mEXNEX$ {}_{c} $, Daniells et al. 2023) model, within which a baskets’ exchangeability weight is computed using a data-driven approach. The original mEXNEX$ {}_{c} $ approach applies simple independent Beta-Binomial models to each basket, then utilizes the average Hellinger distance between the resulting posteriors in order to compute $ \pi_{k} $. For mEXNEX$ {}_{\mathrm{hist}} $, rather than computing the Hellinger distance between current baskets, it is computed between the posteriors of pooled historic response data. To find the posterior distributions, for each of the $ K^{*} $ historic baskets, pool the results of all $ H_{k} $ studies associated with basket $ k $ and define $ \hat{y}_{k^{*}}=\sum_{j\,=\,1}^{H_{k}}y_{k^{*^{(j)}}} $ and $ \hat{n}_{k^{*}}=\sum_{j\,=\,1}^{H_{k}}n_{k^{*^{(j)}}} $. Simple Beta-Binomial models are fit to each of the historic responses $ \hat{y}_{k^{*}} $ with an uninformative Beta(1,1) prior implemented. For baskets with historic information, the probability of exchangeability is set as the average Hellinger distance between all historic baskets:


(4.9) \begin{align*}\pi_{k}=\sum\limits_{i^{*}=1, i^{*}\neq k^{*}}^{K^{*}}\frac{1-h_{i^{*},k^{*}}}{K^{*}-1}\quad\text{for }k=1, \ldots, K^{*},\end{align*}

where $ h_{i^{*},k^{*}} $ is the Hellinger distance between the historic baskets (consisting of pooled data) for two current baskets $ i $ and $ k $. For baskets without historic information, define their probabilities of exchangeability as:


(4.10) \begin{align*}\pi_{k}=\zeta_{k}\sum^{K^{*}}_{i=1}\frac{\pi_{i}}{K^{*}}\quad\text{for }k=K^{*}+1, \ldots, K, \end{align*}

that is as the average probability of exchangeability of those baskets that *do* have historic data available, down-weighted by a scalar, $ \zeta_{k}\in[0,1] $. The purpose of this $ \zeta_{k} $ is to account for uncertainty in the probability of exchangeability in baskets in which there is no previously observed data. Due to the exchangeability assumption, a priori it is believed that those with and without historic data are exchangeable and thus in this approach it is assumed the $ \pi_{k} $ values will be similar for those without historic data. However, this assumption may not hold, in which case the exchangeability for baskets without previous data may not equate to those with historic data. The scalar $ \zeta_{k} $ limits the potential impact this could have on inflated error rates.

### 4.2. Prior and parameter choices

The six models for comparison are as follows:

1. **Ind**: An independent/stratified analysis for each basket, i.e no information shared between current baskets *or* from historic data.
2. **EXNEX**: the EXNEX model independent of any historic information as described in Model 3.7.
3. **EXNEX$ {}_{\mathrm{pool}} $**: an EXNEX model which incorporates historic information by pooling the results of basket $ k $ and all results from the $ H_{k} $ previous studies. An EXNEX model as described in Model 3.7 is applied to the pooled responses. This takes into account that the historic baskets are associated with a specific basket on the current trial.
4. **mEXNEX$ {}_{\mathrm{hist}} $**: a modified EXNEX approach with data-driven exchangeability weights based on the historic data.
5. **EXppNEX**: an EXNEX model with a power prior placed on the NEX component.
6. **MLMixture**: a multi-level mixture model consisting of two EXNEX models.

All of the models consist of several priors and parameters. For the EXNEX, EXNEX$ {}_{\mathrm{pool}} $, EXppNEX and the MLMixture models equal mixture weights of 0.5 were utilized throughout to fully allow the model to update the weights based on homogeneity/heterogeneity. Other values of $ \pi_{\lambda_{k}} $, $ \pi_{\mathrm{all},i} $ and $ \pi_{\mathrm{curr},i} $ were considered for the MLMixture model and further discussed later in this paper, however, the choice of equal weights throughout demonstrated a good balance between error control and power improvement. For EXppNEX the power prior parameter, $ \alpha $, was set at 0.5 in order to discount the historical information in the informative prior. Alternative $ \alpha $ values are later discussed when investigating sensitivity, the findings of which suggest setting $ \alpha\,=\,0.5 $ as a reasonable choice. Full model outlines are given in Appendix S1 of the Supplementary Materials.

For all borrowing methods, a hierarchy is placed on an EX component within which hyper-priors are placed on the common mean $ \mu $ and borrowing parameter $ \sigma $. For each of the approaches which possess an EX component, the hyper-priors $ \mu\sim\mathrm{N}(\mathrm{logit}(q_{0}),10^{2}) $ and $ \sigma\sim\mathrm{Half-Normal}(0,1) $ are applied as suggested by Neuenschwander et al. (2016). For the mEXNEX$ {}_{\mathrm{hist}} $ model, a historic scalar of $ \zeta_{k}=0.8 $ is implemented to down-weight the contribution of historic data to borrowing in the former and to reduce the prior borrowing probability for baskets with unobserved historic data in the latter.

### 4.3. Simulation results

**Fig. 1.**
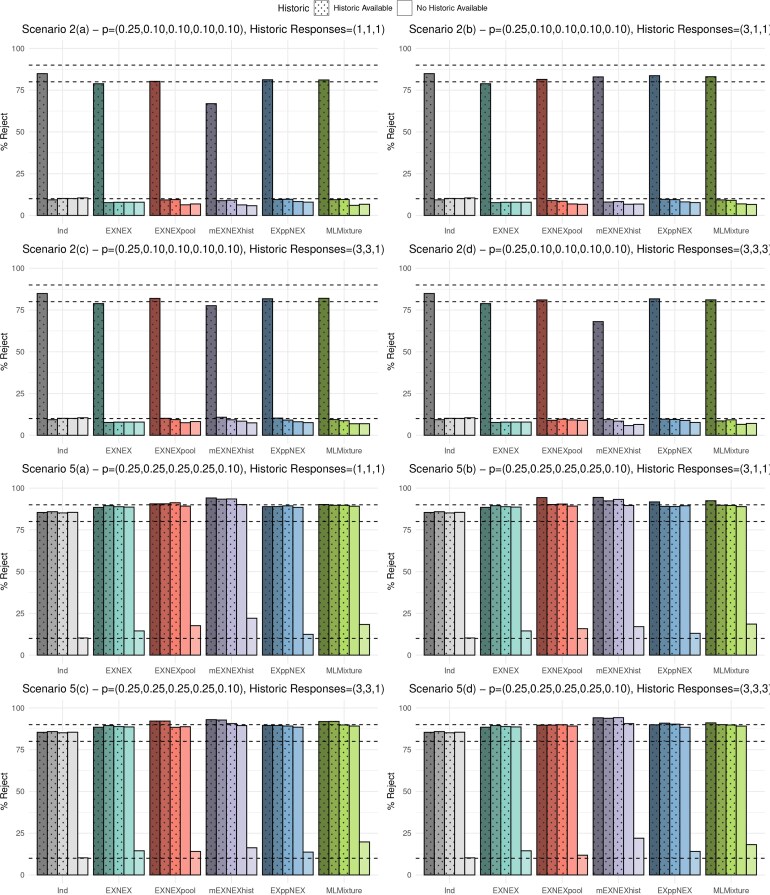
Simulation results: type I error rate and power under each of the six approaches for scenarios 2 and 5 cases a) to d).

**Fig. 2.**
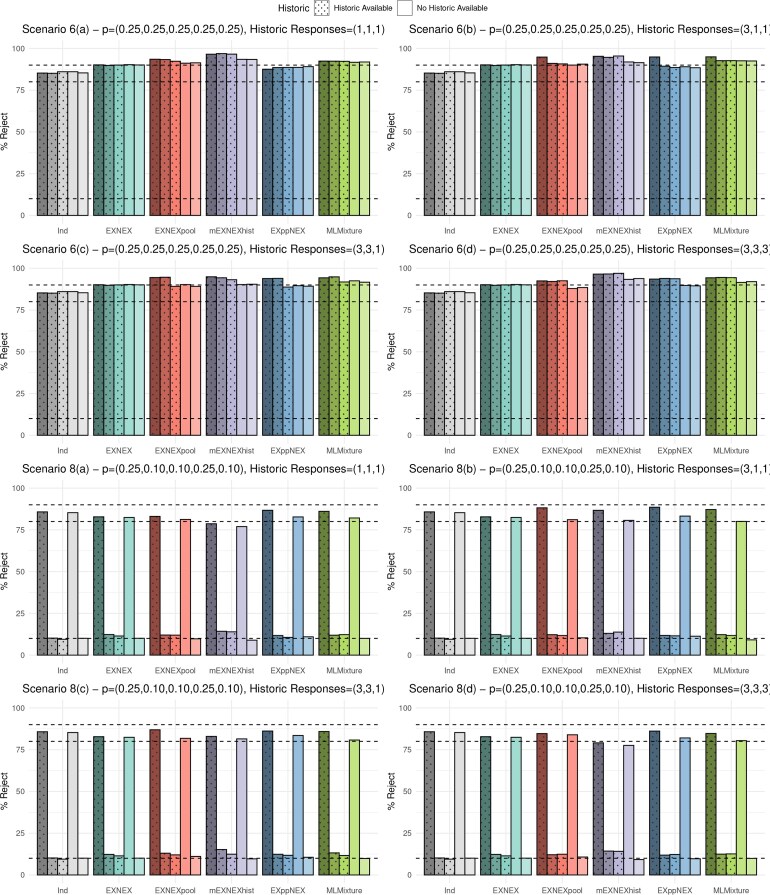
Simulation results: type I error rate and power under each of the six approaches for scenarios 6 and 8 cases a) to d).

The results under four of the eight scenarios are presented in Figs 1 and 2, which show the type I error rate and power for each of the five baskets under all six modeling approaches. Dashed lines are provided to highlight the nominal 10% type error rate and 80% power, as well as, 90% in order to distinguish power improvement between approaches when the nominal level is exceeded. Scenarios 2 and 5 were selected to demonstrate the more “extreme” cases wherein just a single baskets is effective or ineffective respectively. These two scenarios tend to give the lowest power and highest error inflation respectively and thus assessment of the performance of the approaches under these cases is most compelling. Scenario 6 is the global alternative best demonstrates power improvement across the approaches. Scenario 8 differs as it consists of a basket without historic information being effective to the treatment alongside just a single effective basket with historic information. Thus this scenario allows a comparison in power dependent on whether or not a basket has historic information. The plotted results of the remaining four scenarios are given in the Supplementary Materials.

Beginning with scenario 2, the power under the EXNEX model lies below the nominal level at 78.9%. The mEXNEX$ {}_{\mathrm{hist}} $ model also fails to reach the nominal level in almost all cases with power as little as 66.9%. In cases (a) and (d), responses in historic baskets are identical, thus in the mEXNEX$ {}_{\mathrm{hist}} $ approach, the probabilities of exchangeability $ \pi_{k}=1 $ for baskets with historic information and $ \pi_{k}=0.8 $ for those without. This results in strong borrowing so the posterior for the one and only effective basket is pulled down toward the four ineffective baskets, resulting in a loss in power. In fact, an independent analysis has the highest power under scenario 2 as any borrowing in this instance will reduce the power due to the pull in posterior.

All approaches under scenario 2 have type I errors at or below the nominal 10% level. Only minute differences are observed between the EXNEX$ {}_{\mathrm{pool}} $, EXppNEX, and MLMixture approaches in terms of power. The EXppNEX model gives marginally higher power under scenario 2(a) at 81.2% compared to the EXNEX model at 78.9%. In cases of homogeneity to the current data, the MLMixture and EXppNEX models have up to a 4.2% increase in power over an EXNEX model, whilst still maintaining error control at or below 10%. The MLMixture model has consistently lower type I error rate than EXppNEX under scenario 2.

Under scenario 5, one basket is ineffective against the treatment and has no historic information available. The independent analysis and EXNEX model demonstrate lower power than the proposed borrowing models. For baskets 1-3, the mEXNEX$ {}_{\mathrm{hist}} $ approach has the highest power in all sub-cases ranging from 90.7-94.4% depending on homogeneity amongst the historic sources. However, this also came with the greatest type I errors in sub-cases (a) and (d) at around 22%. Inflation in error rates is also an issue in the MLMixture model, where the type I error rate across the four sub-cases ranges from 18.2-19.8%, a substantial increase over the EXNEX model which has a 14.5% error rate. In contrast, the EXppNEX approach has reduced error rates compared to the standard EXNEX model ranging from 12.4-14.1% but only shows improvement in power for some baskets, for instance sub-case 5(b) shows the EXppNEX model has an improvement in power in basket 1 at 91.7% compared to 88.5% under the EXNEX model. For the EXNEX$ {}_{\mathrm{pool}} $ approach, the error rate reduces as the number of effective historic baskets increases, resulting in a type I error 2.6% lower than the standard EXNEX model under scenario 5(d).

Under scenario 6 all current baskets have a homogeneous and effective response rate. Under sub-case (a) in which the historic responses are all heterogeneous to the current baskets, the EXNEX$ {}_{\mathrm{pool}} $ gives power of approximately 93.1% for baskets 1 to 3 and 91.2% for baskets 4 and 5. However, under sub-case (d) where all historic responses are homogeneous to the current response data, these power values actually decrease to around 92.4% and 88.2%. This may seem counter-intuitive given that the EXNEX$ {}_{\mathrm{pool}} $ pools the results from current and historic baskets (and hence has the strongest possible borrowing), so in cases of homogeneity one would expect a further increase in power. However, this is not observed due to the calibration of the efficacy decision criteria. Under sub-case (a), the cut-off value for baskets 1 to 3 is 0.826 which is less conservative than the calibrated value obtained under sub-case (d) at 0.966. This is due to the pooling itself, under sub-case (a), the three ineffective historic baskets will always pull the posteriors down, reducing the chance of a type I error, therefore requiring a less stringent cut-off value. This lower $ \Delta_{k} $ value also makes it easier to reject the null, thus improving the power. The higher $ \Delta_{k} $ value in sub-case (d) results in a reduction of power. This highlights the importance of calibration and how, if done correctly, it can drastically change results of any studies.

The mEXNEX$ {}_{\mathrm{hist}} $ model again demonstrates the greatest power under scenario 6 and under sub-case (d) has average power of 96.7% for baskets with historic information and 93.7% for those without. This compares to an average power of 90.1% under the EXNEX model and 85.6% under an independent analysis. The MLMixture appears to handle heterogeneity between historic sources better than the EXppNEX model in terms of power, giving values consistently above 90% in all sub-cases. The EXppNEX approach has lower power than the EXNEX model under (a) at around 88.5%. This reduction is maintained for all baskets and sub-cases in which the historic and current data are conflicting. However, in cases of homogeneity, power can be substantially increased, for instance under scenario 6(b) EXppNEX has power 94.88% for basket 1. The benefits of utilizing information borrowing from both current and historic sources are highlighted in scenario 6(d) in which the responses rates in all current and historic baskets are homogeneous and the treatment is effective in each. In this case, all of the proposed methods demonstrate substantial power gain compared to the EXNEX model.

Finally, in scenario 8 one basket with and without historic information is effective, with the rest ineffective to treatment. The results for scenario 8 show similar findings to those in scenarios 2, 5 and 6. Across all four sub-cases, of the proposed approaches, the EXppNEX model has highest power for both baskets 2 and 4, whilst maintaining a type I error rate close to that of the EXNEX model. The MLMixture model achieves similar power to the EXppNEX model, however demonstrates greater error inflation with maximum error of 13.2%. For comparison, the EXNEX model has maximum error of 12.3%. The EXNEX$ {}_{\mathrm{pool}} $ model provides reasonable power, however, in cases (a)-(c), this power is reduced compared to the EXNEX model for basket 4. This approach also demonstrates error inflation, particularly in the cases in which the historic information is heterogeneous to the current data. An independent analysis gives reasonably good operating characteristics under this scenario giving the highest power for basket 4 across sub-cases (a) to (d).

To summarize, given these results, the EXppNEX model would be recommended due to its superior error control compared to the other approaches considered in this paper, including the standard EXNEX model. The EXppNEX model also substantially improves power in cases of homogeneity between historic and current data sources compared to the EXNEX model. The mEXNEX$ {}_{\mathrm{hist}} $ model is not recommended due to its inconsistent performance, showing substantially decreased power compared to the nominal level under several scenarios. The MLMixture is far more computationally intensive without substantially improving performance in both power and type I error rate compared to the alternative approaches. Although results were only presented here for half of the 32 total scenarios considered, results of the remaining 16 proved similar.

### 4.4. Sensitivity

Although the results in the previous section highlighted fairly substantial error inflation under the MLMixture compared to the standard EXNEX model, this error inflation can be shown to be limited by adjusting the mixture weights $ \pi_{\lambda, k} $, $ \pi_{\mathrm{curr},i} $ and $ \pi_{\mathrm{all},i} $. Table 2 summarizes the operating characteristics across the 8 scenarios under several combinations of mixture weights, with $ \pi_{\lambda, k} $, $ \pi_{\mathrm{curr},i} $ and $ \pi_{\mathrm{all},i} $ taking values 0.25, 0.5 or 0.75. The maximum type I error rate and average power was taken across all 8 scenarios split by basket and by historic sub-case. Both $ \pi_{\mathrm{curr},i} $ and $ \pi_{\mathrm{all},i} $ are set as equal, thus the mixture weights in the two EXNEX models in the MLMixture model follow the same distribution.

**Table 2.** Sensitivity analysis.^a^

		MLMixture weights									
Sub-case	Basket(s)	0.25,0.25		0.25,0.75		0.75,0.25		0.75,0.75		0.50,0.50	
		E	P	E	P	E	P	E	P	E	P
(a) $ y_{k^{*}}=(1,1,1) $	1,2,3	12.12	87.33	14.06	88.34	12.00	86.73	13.48	88.72	12.26	88.53
	4,5	11.86	86.04	22.40	84.43	11.74	86.35	22.86	84.61	18.34	86.81
(b) $ y_{k^{*}}=(3,1,1) $	1	11.46	87.70	12.56	89.77	11.36	87.17	12.19	89.46	11.26	88.12
	2,3	11.84	88.30	13.74	90.41	12.10	87.12	13.56	90.32	12.52	89.57
	4,5	11.72	87.00	22.60	84.69	12.14	85.43	23.34	84.42	18.58	86.37
(c) $ y_{k^{*}}=(3,3,1) $	1,2	11.82	87.05	14.20	88.48	12.44	87.20	15.02	88.24	13.22	88.91
	3	12.46	88.49	13.90	91.86	10.74	88.55	15.56	92.16	13.28	90.69
	4,5	12.16	86.22	23.26	83.78	12.52	86.24	21.60	84.65	19.78	86.24
(d) $ y_{k^{*}}=(3,3,3) $	1,2,3	12.36	87.88	14.02	88.61	12.18	87.49	13.72	88.65	12.58	88.59
	4,5	12.04	85.58	22.26	84.62	12.00	86.06	22.06	83.98	18.16	86.10

		EXppNEX Power Parameter $ \boldsymbol{\alpha} $					
Sub-Case	Basket(s)	0.25		0.5		1	
		E	P	E	P	E	P
(a) $ y_{k^{*}}=(1,1,1) $	1,2,3	11.92	87.49	12.08	87.20	11.98	87.18
	4,5	13.12	86.17	12.42	85.79	12.44	85.88
(b) $ y_{k^{*}}=(3,1,1) $	1	11.84	88.25	11.58	89.24	11.86	88.49
	2,3	12.04	88.48	11.76	88.25	12.14	87.77
	4,5	14.32	86.18	13.04	85.90	13.16	85.66
(c) $ y_{k^{*}}=(3,3,1) $	1,2	12.84	88.40	12.40	88.21	12.26	88.69
	3	12.12	89.12	11.76	88.89	11.76	88.60
	4,5	13.64	86.37	13.70	86.15	12.92	86.05
(d) $ y_{k^{*}}=(3,3,3) $	1,2,3	12.52	88.84	12.26	88.91	12.38	88.68
	4,5	14.32	86.39	14.10	86.02	13.52	86.31

^a^ (1) Weights $ \pi_{\lambda, k} $, $ \pi_{\mathrm{curr},j} $ and $ \pi_{\mathrm{all},j} $ are set at 0.25, 0.5 or 0.75 in the MLMixture model. Settings are labeled as $ x, y $ where $ x=\pi_{\lambda, k} $ and $ y=\pi_{\mathrm{curr},j}=\pi_{\mathrm{all},j} $; (2) power parameter, $ \alpha $, is set at 0.25, 0.5 or 1 in the EXppNEX model. The maximum type I error rate (E) and average power (P) are computed across the 8 scenarios under all 4 sub-cases. The maximum/average is only taken across baskets of the same type, ie with or without historic baskets and only between baskets with an identical number of responses in the historic basket.

Setting all mixture weights to 0.25 showed a reduction in maximum type I error rate (maximum error rate is 12.5%) in all baskets and historic sub-cases compared to both settings in which $ \pi_{\mathrm{curr},i} $ and $ \pi_{\mathrm{all},i} $ are set to 0.75 (maximum type I error rates of 22.6% and 23.3%). Placing a $ \pi_{\mathrm{curr},i}=\pi_{\mathrm{all},i}=0.75 $ weight increases the probabilities of being in both EX components, therefore encouraging borrowing between baskets. In cases of heterogeneity this increased borrowing results in more substantial error inflation over the nominal level. A lower maximum error rate is also observed when using 0.25 for all mixture weights compared to when mixture weights are set to 0.5 (maximum type I error rate of 19.8%) in all bar one setting. Using equal weights of 0.25 across all mixtures gives a reduction in power compared to the setting where $ \pi_{\lambda, k}=0.25 $ and $ \pi_{\mathrm{curr},i}=\pi_{\mathrm{all},i}=0.75 $. The maximum difference in power is a reduction of 3.4%, however an increase of up to 2.4% in power is observed in some baskets. Both cases in which $ \pi_{\mathrm{curr},i}=\pi_{\mathrm{all},i}=0.25 $ produce similar maximum type I error rate and average power regardless of the choice of $ \pi_{\lambda, k} $. Using equal weights of 0.5 for all mixtures balances the error control observed under the equal mixture weights of 0.25 with the improved power of setting $ \pi_{\mathrm{curr},i}=\pi_{\mathrm{all},i}=0.75 $. Results of the full simulation study exploring weights in the MLMixture model are presented in the Supplementary  Materials.

Similarly, in the conducted simulation study, only a single value of power parameter $ \alpha $ was considered and set to allow for a moderate amount of borrowing. As stated throughout the literature, there is difficulty surrounding the selection of $ \alpha $, as operating characteristics can be highly dependent on the value (Duan et al. 2006). Three alternative values of $ \alpha $ were considered: 0.25, 0.5, and 1 are now considered. A choice of $ \alpha\,=\,1 $ fully incorporates the historic data in the NEX component with no discounting, whilst 0.25 discounts the historic data heavily. This simulation study has the same setting as in the previous simulation study, with only the power parameter varied. The maximum type I error rate and average power across the 8 scenarios are also presented in Table 2, split by historic sub-case and basket. All power values are consistent across all choices of $ \alpha $, ranging by no more than 1%. Slightly more variation is observed in the maximum type I error rate, with $ \alpha\,=\,0.25 $ giving marginally higher error rates in almost all baskets and sub-cases. A choice of $ \alpha\,=\,0.5 $ results in the smallest error in almost all cases, with an improvement in power compared to $ \alpha\,=\,1 $ in most cases too. Full results of this simulation study are presented in Appendix S8 of the Supplementary Materials.

Note that a sensitivity analysis was not conducted for other model parameters such as the prior distributions on the exchangeable components, as this has already been explored extensively in the literature. Thus this analysis was focused on the model parameters for which the sensitivity was unknown. However, similar simulation studies could be conducted prior to a trial in order to fine-tune these parameters alongside the power parameters and mixture weights. As model parameters are difficult to specify given the limited information prior to the trial, particularly on the homogeneity between the historic and current data, it is recommended that such a sensitivity analysis be conducted in order to explore the effect of changing the model parameters on inference. As historic data is already available at this stage, only data for the current trial needs to be simulated under several possible data scenarios. The models are applied with varying prior parameter values. The operating characteristics are compared and an optimal parameter value selected based on balancing the trade-off between type I error control and maximizing power.

## 5. EXAMPLE TRIAL ANALYSIS

**Fig. 3.**
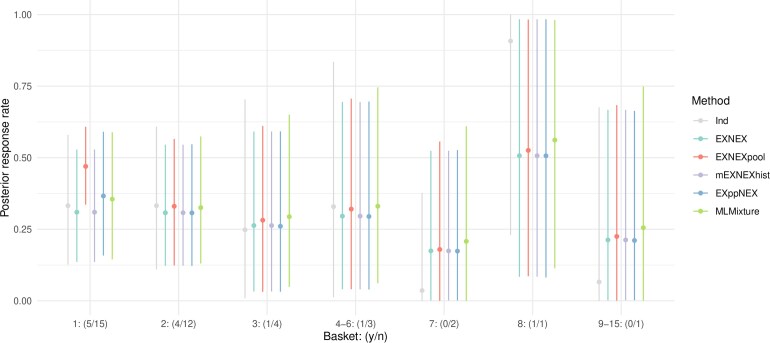
Mean posterior probabilities of a response (and 95% confidence intervals) in each of the baskets under the various borrowing approaches when fit to the BELIEVE trial data, with historic information drawn from the ROAR trial for basket 1. Baskets 9 to 15 had the same sample size and number of responses and have hence been combined, as have baskets 4 to 6.

In order to demonstrate how the models would perform in practice, the results of actual trial data from the BELIEVE trial (Shimoi et al. 2024) are now re-analyzed using the proposed approaches with historic information drawn from the ROAR trial (Subbiah et al. 2023). The BELIEVE trial consisted of 15 baskets, one of which (thyroid cancer) was in common with the ROAR trial. In the ROAR trial, the thyroid cancer basket consisted of 36 patients, out of which 20 responded to the treatment, whilst in the BELIEVE trial, the same basket consisted of 15 patients, out of which 5 responded. The posterior estimates for the response rates in all baskets, obtained under each of the borrowing approaches, are presented in Fig. 3. Note baskets 4-6 and 9-15 have been combined as they each observed the same sample size and number of responses.

Considering the thyroid cancer basket, where historic information was available from the ROAR trial, the EXNEX$ {}_{\mathrm{pool}} $ approach results in the smallest standard deviation due to the increased pooled sample size from 15 to 51 but also pulls the posterior point estimates up toward the historic ORR. The EXppNEX and MLMixture models give the same standard deviation as the independent analysis with higher point estimates. Due to the heterogeneity between the current and historic ORRs, these models do not borrow strongly from the historic data, however some information is shared hence the differing point estimates to the EXNEX model. The mEXNEX$ {}_{\mathrm{hist}} $ is equivalent to the EXNEX model in this setting as only a single basket has historic information available. For baskets without historic information, an independent analysis gives point estimates closest to the ORRs presented in the trial results, however, in cases where baskets consist of $ n\geq 3 $ patients (baskets 1-6), the standard deviations are larger than all of the borrowing approaches. The preferred EXppNEX model from the simulation study has similar standard deviations and posterior response rates to the standard EXNEX model for baskets without historic information. Posterior point estimates are generally higher under the MLMixture model compared to the EXppNEX. For basket 8 in which the one recruited patient responded to the treatment, the independent analysis gives a point estimate of 0.91. Under all borrowing approaches this is pulled down to around 0.5 with a much larger standard deviation. This reflects the uncertainty of the point estimate when the response in just a single patient is observed.

Overall, as the true ORR is not known, it is challenging to draw conclusions regarding the model performance from a single model fit. However, the key results are the change in standard deviations dependent on the method used, with borrowing approaches generally increasing the precision when sample sizes are larger than 3.

## 6. DISCUSSION

In this paper, several approaches for borrowing from both historic and current baskets under one framework are proposed. Most approaches built on the EXNEX model which has previously been implemented to borrow information between baskets on the current trial. This model was used as a basis due to its popularity in the field of basket trials and due to its flexible structure in terms of allowing both borrowing and an independent analysis in one model. The conclusion of the simulation study presented in this paper favored the use of the EXppNEX approach, however, it is stated throughout the literature that the performance of a power prior is sensitive to the choice of power parameter, $ \alpha $. This was explored in another simulation study, where results demonstrated that in this simulation setting, the choice of power parameter had minimal effect on operating characteristics. There are several possible contributions to the insensitivity to $ \alpha $ in this setting, the most prominent of which is the calibration of efficacy criteria. The efficacy criteria were calibrated under each of the choices of $ \alpha $ separately, controlling the average type I error rate to 10% across the considered scenarios. Therefore, the type I error is expected to be similar for each of the $ \alpha $ values, and as in turn will the power. Another contributing factor is the mixture of the power prior with the BHM in the EXppNEX approach. The weight placed on both components could impact sensitivity to the choice of $ \alpha $. In the presented simulation study the heterogeneity between baskets with an ineffective and effective response are not too substantial and thus still a relatively large weight will be placed on the EX component rather than concentrating the weight on the power prior, thereby limiting the impact of the power prior itself. Alternative approaches to the power prior such as the modified power prior (Duan et al. 2006) or the commensurate prior (Hobbs et al. 2011) could also be implemented in place of the power prior in the EXppNEX approach, most of which eliminate the need to specify the power parameter. However, no such methods were considered in this paper. We also considered replacing the power prior in the EXppNEX model with a further BHM or EXNEX model to borrow information between the current basket and its own associated historic data. However, in cases where there is only one or two sources of historic information, it is difficult to accurately estimate the degree of of exchangeability between the current and historic baskets. It is also important to consider that such alternative approaches would increase the model complexity and thus make these approaches more difficult to implement in practice.

The Robust calibration procedure (RCaP) was implemented in the simulation studies to calibrate efficacy criteria. Should calibration have been conducted under the traditional approach of calibrating under the global null, more substantial error inflation would have been observed and with that a greater improvement in power would also be present, however, the comparison of approaches remained the same, with differences between their performance slightly more pronounced. The calibration of these efficacy cut-off values is a key component to any simulation study and heavily impact operating characteristics. This is evident in some of the results observed in this paper, particularly when comparing the EXNEX and EXNEX$ {}_{\mathrm{pool}} $ approaches as their cut-off values, $ \Delta_{k} $, varied so much in their conservative nature. Other approaches to calibrate model hyper-parameters and tuning parameters have also been proposed to aide in optimizing operating characteristics. Kaizer et al. (2021) propose the use of a weighted sum of the FWERs to provide error control when utilizing a multisource-exchangeability model (MEM), whilst Jiang et al. (2021) select the prior on the borrowing parameter based on maximizing a utility function that balances the trade-off in error control and power improvement. The impact of implementing these calibration approaches has not yet been explored but it is expected that results would be similar to those presented in this paper.

The proposed models could be extended to increase their sensitivity to the presence of heterogeneity between current baskets and to historic data sources. The MLMixture as it stands does not control error rates to the nominal level in cases of heterogeneity across all baskets current and historic. Simulations found that more weight was placed on the informative NEX prior than was desirable. A weight metric was considered to shift these weights based on homogeneity of response data, but this inflated error and decreased power in some cases. Also, the computational cost of the MLMixture model means that it quickly becomes infeasible to conduct large-scale simulations as the number of current and historic baskets increases. A comparison of computational time of all proposed approaches is presented in the Supplementary Material.

Additionally, this paper focused on a simulation setting where design parameters were motivated by the MyPathway and VE-BASKET trials, with sample sizes of 34 and 13 used in the current and historic baskets respectively. These were based on the planned sample sizes for both studies. A sample size of 34 patients is rather large for a basket trial which typically studies rare diseases. In fact, in the MyPathway study, in the BRAFV600 mutation branch of the trial, not a single basket achieved this planned sample size, with a total of 55 patients ultimately recruited across the five baskets. The use of the larger sample size in the simulation study down-plays the benefits of borrowing from the historic information, as smaller baskets benefit more greatly from this additional source of information. To address this, a further simulation study was conducted with the sample size in the current study reduced from 34 to 20 patients with all other design parameters kept the same. Results can be found in the Supplementary Materials. The results demonstrate comparable findings between the performance of methods as presented in this paper, however, due to the smaller sample size, the nominal power value is rarely reached and the difference between the independent analysis and the borrowing models substantially increases in some scenarios. In fact, the nominal power of 80% is not achieved using the standard EXNEX model in which historic data is ignored, thus encouraging the use of the proposed historical borrowing techniques. An additional simulation study was conducted that assumed unequal sample sizes for the historic studies, with the historic data set at the number of responses and sample size as observed in the VE-BASKET trial. The results of this study can also be found in the Supplementary Materials. Further simulations could be conducted to alter other design parameters such as the number of sources of historic information and the sample sizes in the current baskets.

Finally, a limitation of this work is the absence of interim analysis and early stopping for efficacy/futility. Interim analyses in basket trials has been considered throughout the literature and come with the advantage of reducing the required sample sizes and terminating baskets that do not show promise early. This will reduce the type I error rate but also risks a reduction in power (Zhou and Ji 2024). Conducting an interim analyses in basket trials poses its own challenges, particularly due to the small sample sizes. The lack of data at interim will limit the ability to accurately estimate the exchangeability between baskets and could substantially bias estimates when information borrowing models are implemented. Therefore, such interims were excluded from this work to focus on the impact of including historic information only, however, the methods could be extended to include such adaptive features.

## Supplementary Material

kxaf016_Supplementary_Data

## Data Availability

All simulations were conducted through the computing software JAGS in R through the “rjags” package (Plummer 2023). No new data have been used in this publication. Simulations can be reproduced using the open accessible code available at https://github.com/LibbyDaniells/HistoricBorrowingBasketTrial.
