# Supplementary material for: Incorporating historic information to further improve power when conducting Bayesian information borrowing in basket trials
Source: Biostatistics. 2025 Jun 18;26(1):kxaf016. doi: 10.1093/biostatistics/kxaf016 (PMC12204204; doi:10.1093/biostatistics/kxaf016)
Supplement: kxaf016_Supplementary_Data [file kxaf016_supplementary_data.zip › biosts-24269-File002.pdf]

Biostatistics (2025), **0**, 0, *pp.* 1–**??**
doi:10.1093/biostatistics/output

Incorporating historic information to further improve power when conducting Bayesian information borrowing in basket trials: Supplementary Material

Libby Daniells[1*], Pavel Mozgunov[2], Helen Barnett[3],

Alun Bedding[4], Thomas Jaki[2,5]

[1]*STOR-i Centre for Doctoral Training, Department of Mathematics and Statistics, Lancaster University, Lancaster, UK.* [2]*MRC Biostatistics Unit, University of Cambridge, Cambridge, UK.* [3]*Department of Mathematics and Statistics, Lancaster University, Lancaster, UK.* [4]*Roche Products Ltd, Welwyn Garden City, UK.* [5]*Faculty of Informatics and Data Science, University of Regensburg, Regensburg, Germany.*

libby.daniells@mrc-bsu.cam.ac.uk

APPENDIX

A. Models

Presented below are the full model specifications for the simulation study presented in the main text, including all parameter choices and prior specifications. Note that for this simulation study $H_k = 1$ for $k = 1, 2, 3$ and $H_k = 0$ for $k = 4, 5$ and thus only one source of historic information was included, allowing the superscript j to distinguish the historical study, to be dropped.

1. **Ind:**

© The Author 2025. Published by Oxford University Press. All rights reserved. For permissions, please e-mail: journals.permissions@oup.com

$$Y_k \sim \text{Binomial}(n_k, p_k), \qquad k = 1, 2, 3, 4, 5,$$

$$\theta_k = \text{logit}(p_k) \sim \text{N}(\text{logit}(0.1), 10^2).$$

2. **EXNEX:**

$$\begin{aligned} & Y_k \sim \text{Binomial}(n_k, p_k), \quad k = 1, 2, 3, 4, 5, && \theta_{1k} = \text{logit}(M_{1k}) \sim \text{N}(\mu, \sigma^2), \text{ (EX)} \\ & p_k = \delta_k M_{1k} + (1 - \delta_k) M_{2k}, && \mu \sim \text{N}(\text{logit}(0.1), 10^2), \\ & \delta_k \sim \text{Bernoulli}(\pi_k), && \sigma \sim \text{Half-Normal}(0, 1), \\ & && \theta_{2k} = \text{logit}(M_{2k}) \sim \text{N}(-1.386, 6.25^2). \text{ (NEX)} \end{aligned}$$

with $\pi_k = 0.5$ for $k = 1, 2, 3, 4, 5$.

3. **EXNEX$_{\mathbf{pool}}$:**

$$\begin{aligned} & Y_k = y_k + y_{k^*} \sim \text{Binomial}(n_k + n_{k^*}, p_k), \quad k = 1, 2, 3, 4, 5, && \theta_{1k} = \text{logit}(M_{1k}) \sim \text{N}(\mu, \sigma^2), \text{ (EX)} \\ & p_k = \delta_k M_{1k} + (1 - \delta_k) M_{2k}, && \mu \sim \text{N}(\text{logit}(0.1), 10^2), \\ & \delta_k \sim \text{Bernoulli}(\pi_k), && \sigma \sim \text{Half-Normal}(0, 1), \\ & && \theta_{2k} = \text{logit}(M_{2k}) \sim \text{N}(-1.386, 6.25^2). \text{ (NEX)} \end{aligned}$$

where $y_{k^*} = n_{k^*} = 0$ for $k = 4, 5$, i.e. in baskets without historic information available. Mixture weights are $\pi_k = 0.5$ for $k = 1, 2, 3, 4, 5$.

4. **mEXNEX$_{\mathbf{hist}}$:** same as the EXNEX model above but with

$$\pi_k = \sum_{i^*=1, i^* \neq k^*}^{3} \frac{1 - h_{i^*,k^*}}{3 - 1} \quad \text{for } k = 1, 2, 3, \quad \text{then} \quad \pi_k = 0.8 \sum_{i=1}^{3} \frac{\pi_i}{3} \quad \text{for } k = 4, 5,$$

where h_{i^*,k^*} is the Hellinger distance between the posteriors of two historic baskets i^* and k^*.

5. **EXppNEX:**

$$
\begin{aligned}
&Y_k \sim \text{Binomial}(n_k, p_k), \quad k = 1,2,3,4,5, && \theta_{1k} = \text{logit}(M_{1k}) \sim \text{N}(\mu, \sigma^2), \quad \text{(EX)}\\
&p_k = \delta_k M_{1k} + (1-\delta_k) M_{2k}, && \mu \sim \text{N}(\text{logit}(0.1), 10^2),\\
&\delta_k \sim \text{Bernoulli}(\pi_k), && \sigma \sim \text{Half-Normal}(0,1),\\
&\mathbb{I}_k = 1 \text{ if } y_{k^*} \text{ exists for basket } k, && M_{2k} = \mathbb{I}_k P_{1k} + (1-\mathbb{I}_k) P_{0k},\\
& && P_{1k} \sim \text{Beta}(1 + \alpha y_{k^*}, 1 + \alpha(n_{k^*} - y_{k^*})),\\
& && \theta_{2k} = \text{logit}(P_{0k}) \sim \text{N}(-1.386, 6.25^2),
\end{aligned}
$$

with $\pi_k = 0.5$ for $k = 1,2,3,4,5$ and $\alpha = 0.5$ for all baskets.

6. **MLMixture:**

$$
\begin{aligned}
&Y_i \sim \text{Binomial}(n_i, p_i) \qquad i = 1,2,3,4,5,1^*,2^*,3^*,\\
&\psi_i = \begin{cases} 1 & \text{if basket } i \text{ is a historic basket,}\\ 0 & \text{otherwise.}\end{cases}
\end{aligned}
$$

$$
\begin{aligned}
&\text{EXNEX}_{\text{all},i} = \delta_{i,\text{all}} M_{\text{all},1i} + (1-\delta_{i,\text{all}}) M_{\text{all},2i},\\
&\delta_{i,\text{all}} \sim \text{Bernoulli}(\pi_{\text{all},i}),\\
&\theta_{\text{all},1i} = \text{logit}(M_{\text{all},1i}) \sim \text{N}(\mu_{\text{all}}, \sigma^2_{\text{all}}),\\
&\mu_{\text{all}} \sim \text{N}(\text{logit}(0.1), 10^2),\\
&\sigma_{\text{all}} \sim \text{Half-Normal}(0,1),\\
&M_{\text{all},2i} \sim \text{Beta}\left(1 + (1-\psi_i)\sum_{t=1}^{H_i} y_{i^{*(t)}}, 1 + (1-\psi_i)\sum_{t=1}^{H_i}(n_{i^{*(t)}} - y_{i^{*(t)}})\right)\\
&\text{EXNEX}_{\text{curr},i} = \delta_{i,\text{curr}} M_{\text{curr},1i} + (1-\delta_{i,\text{curr}}) M_{\text{curr},2i},\\
&\delta_{i,\text{curr}} \sim \text{Bernoulli}((1-\psi)\pi_{\text{curr},i}),\\
&\theta_{\text{curr},1i} = \text{logit}(M_{\text{curr},1i}) \sim \text{N}(\mu_{\text{curr}}, \sigma^2_{\text{curr}}),\\
&\mu_{\text{curr}} \sim \text{N}(\text{logit}(0.1), 10^2),\\
&\sigma_{\text{curr}} \sim \text{Half-Normal}(0,1),\\
&M_{\text{curr},2i} \sim \text{Beta}(1,1),
\end{aligned}
$$

with

$$p_k = \lambda_k \text{EXNEX}_{\text{all},k} + (1-\lambda_k)\text{EXNEX}_{\text{curr},k}, \quad k = 1,2,3,4,5, \tag{A.1}$$

$$\lambda_k \sim \text{Bernoulli}(\pi_{\lambda,k}). \tag{A.2}$$

All mixture weights are set equal such that $\pi_{\lambda,k} = 0.5$ for all $k = 1,2,3,4,5$ and $\pi_{\text{curr},i} = \pi_{\text{all},i} = 0.5$ for all $i = 1,2,3,4,5,1^*,2^*,3^*$.

The models' prior parameter choices are also summarised in Table 1.

Table 1. *Prior and parameter choice for the simulation study.*

Model	Parameters and Priors
EXNEX	$\pi_k = 0.5$ for $k = 1,\ldots$,K, $m_\mu = \text{logit}(0.1)$, $\nu_\mu = 10^2$, $g(\cdot)$=Half-Normal(0,1), m_k and ν_k are computed based on $\rho_k = 0.2$.
$\text{EXNEX}_{\text{pool}}$	$\pi_k = 0.5$ for $k = 1,\ldots$,K, $m_\mu = \text{logit}(0.1)$, $\nu_\mu = 10^2$, $g(\cdot)$=Half-Normal(0,1), m_k and ν_k are computed based on $\rho_k = 0.2$.
$\text{mEXNEX}_{\text{hist}}$	$m_\mu = \text{logit}(0.1)$, $\nu_\mu = 10^2$, $g(\cdot)$=Half-Normal(0,1), m_k and ν_k are computed based on $\rho_k = 0.2$, ζ_k=0.8.
EXppNEX	$\pi_k = 0.5$ for $k = 1,\ldots$,K, $m_\mu = \text{logit}(0.1)$, $\nu_\mu = 10^2$, $g(\cdot)$=Half-Normal(0,1), m_k and ν_k are computed based on $\rho_k = 0.2$, $a_k = b_k = 1$, $\alpha_j = 0.5$ for $j = 1,\ldots,H_k$ for all k.
MLMixture	$\pi_{\lambda,k} = 0.5$ for $k = 1,\ldots,K$, $\pi_{\text{all},i} = \pi_{\text{curr},i} = 0.5$ and $a_i = b_i = 1$ for $i = 1,\ldots,K,1^*,\ldots,K^*$, $m_{\mu_{\text{all}}} = m_{\mu_{\text{curr}}} = \text{logit}(0.1)$, $\nu_{\mu_{\text{all}}} = \nu_{\mu_{\text{curr}}} = 10^2$, $g(\cdot)$=$f(\cdot)$=Half-Normal(0,1).

B. Robust Calibration Procedure (RCaP)

Algorithm 1 describes the Robust Calibration Procedure (RCaP, Daniells *and others*, 2024) specifically for the control of the type I error rate. Recall that in the simulation study in the main text, calibration of efficacy thresholds, Δ_k, was conducted separately for the four historical data settings: (a) $y_{k^*} = (1,1,1)$, (b) $y_{k^*} = (3,1,1)$, (c) $y_{k^*} = (3,3,1)$ and (d) $y_{k^*} = (3,3,3)$. Therefore the RCaP was conducted four times with all 8 scenarios listed in Table 2 of the main text included. As sample sizes were equal, for baskets with identical historic data, Δ_k's are set as equal and to the basket whose RCaP calibrates across the most scenarios. For example, in historic setting (b)

in which the 2nd and 3rd baskets are identical, Δ_2 is set as the value of Δ_3 obtained from the RCaP procedure. This is because basket 3 is calibrated across scenarios 1, 2, 3, 7 and 8 (i.e. cases where the true response rate of p_3 is null), whereas, basket 3 is only null under scenarios 1, 2, 7 and 8, thus better error control is expected if Δ_3 is set as the efficacy cut-off for both baskets 2 and 3.

Algorithm 1: RCaP - Calibrate Δ_k across several simulation scenarios for type I error rate.

Data: Total number of simulation scenarios, M, scenarios $\boldsymbol{p_1}, \ldots, \boldsymbol{p_M}$, basket sample sizes $\boldsymbol{n}$, number of simulation runs for each scenario, R, weights for the scenarios, $\omega_1, \ldots, \omega_M$ and null response rate, q_0;

Initialization: $\boldsymbol{PP_1}, \ldots, \boldsymbol{PP_K}$;

```
for m = 1 to M do
    for r = 1 to R do
        Generate data X ~ Binomial(p_m, n);
        Fit information borrowing model to obtain posterior densities;
        for k = 1 to K do
            Compute the posterior probability of a type I error P(p_mk > q_0|X), in basket k;
            if T(p_mk ⩽ q_0) then
                for j = 1 to ω_m do
                    PP_k = PP_k ∪ P(p_mk > q_0|X);
```

$\Delta_k = 100(1-\alpha)\%$ quantile of $\boldsymbol{PP}_k$ for each basket k.;

Result: Cut-off values Δ_k for each basket k;

C. Calibrated Δ_k Values under the RCaP

Table 2. *Calibrated Δ_k values obtained using the RCaP procedure across the 8 scenarios presented in the main text. This is conducted under each of the four historic data settings separately.*

**Method**	$\mathbf{p_1}$	$\mathbf{p_2}$	$\mathbf{p_3}$	$\mathbf{p_4}$	$\mathbf{p_5}$
$\mathbf{y_{k^*}}$	**1**	**1**	**1**		
Independent	0.893	0.893	0.893	0.893	0.893
EXNEX	0.900	0.900	0.900	0.900	0.900
$\text{EXNEX}_{\text{pool}}$	0.826	0.826	0.826	0.902	0.902
$\text{mEXNEX}_{\text{hist}}$	0.914	0.914	0.914	0.935	0.935
EXppNEX	0.897	0.897	0.897	0.889	0.889
MLMixture	0.863	0.863	0.863	0.899	0.899
$\mathbf{y_{k^*}}$	**3**	**1**	**1**		
Independent	0.893	0.893	0.893	0.893	0.893
EXNEX	0.900	0.900	0.900	0.900	0.900
$\text{EXNEX}_{\text{pool}}$	0.944	0.838	0.838	0.905	0.905
$\text{mEXNEX}_{\text{hist}}$	0.857	0.894	0.894	0.909	0.909
EXppNEX	0.909	0.895	0.896	0.890	0.890
MLMixture	0.895	0.875	0.875	0.911	0.911
$\mathbf{y_{k^*}}$	**3**	**3**	**1**		
Independent	0.893	0.893	0.893	0.893	0.893
EXNEX	0.900	0.900	0.900	0.900	0.900
$\text{EXNEX}_{\text{pool}}$	0.955	0.955	0.851	0.912	0.912
$\text{mEXNEX}_{\text{hist}}$	0.891	0.891	0.891	0.912	0.912
EXppNEX	0.917	0.917	0.889	0.884	0.884
MLMixture	0.917	0.917	0.885	0.921	0.921
$\mathbf{y_{k^*}}$	**3**	**3**	**3**		
Independent	0.893	0.893	0.893	0.893	0.893
EXNEX	0.900	0.900	0.900	0.900	0.900
$\text{EXNEX}_{\text{pool}}$	0.966	0.966	0.966	0.918	0.918
$\text{mEXNEX}_{\text{hist}}$	0.912	0.912	0.912	0.933	0.933
EXppNEX	0.916	0.916	0.916	0.879	0.879
MLMixture	0.930	0.930	0.930	0.930	0.930

D. Simulation Results: Additional Simulation Scenarios

Figures 1 and 2 present the results of the additional simulation scenarios. Table 3 presents the average power and maximum type I error across all 8 data scenarios for all methods and historic sub-case and the maximum type I error inflation, the type I error inflation is far more substantial in baskets without historic information under the mEXNEX$_{\text{hist}}$ approach, as well as the MLMixture model. The EXppNEX has far better error control across all five baskets in the trial with a maximum of just 14.1% which occurs in sub-case (d) for baskets 4 and 5. Under this sub-case, the MLMixture model gives maximum error of 18.2% and mEXNEX$_{\text{hist}}$ 22%. This reduction in error rate under the EXppNEX approach does come alongside a reduction in power compared to the EXNEX model in a handful of cases, however, this reduction does not exceed 0.5%. That being said, power is improved in several cases, with power improving up to 4.2% over the EXNEX model. The MLMixture model produces similar average power to the EXppNEX approach, improving over the EXNEX model by up to 3.8%, however, has substantially increased error rates in baskets 4 and 5. The performance of mEXNEX$_{\text{hist}}$ fluctuated substantially between baskets and sub-cases, with mEXNEX$_{\text{hist}}$ giving the highest average power under sub-case (b) but substantially reduced power for baskets 4 and 5 under sub-case (d). The EXNEX$_{\text{pool}}$ model presented similar average power values to the EXppNEX approach, with a higher power in a number of sub-cases, however, error rates were consistently higher. Finally, the independent analysis produced an average power less than that of the EXppNEX model in all cases.

Table 3. *The average power and the maximum type I error rates, computed across the 8 scenarios under all 4 historic data sub-cases. Note that the average is only taken across baskets of the same type i.e. with or without historic baskets and only between baskets with an identical number of responses in the historic basket.*

		**Average Power**					
**Sub-Case**	**Basket(s)**	Independent	EXNEX	$\text{EXNEX}_{\text{pool}}$	$\text{mEXNEX}_{\text{hist}}$	EXppNEX	MLMixture
(a)	1,2,3	85.58	86.65	88.53	88.44	87.20	88.53
	4,5	85.57	86.28	86.28	83.56	85.79	86.81
(b)	1	85.47	85.05	89.64	89.99	89.24	88.82
	2,3	85.67	88.03	89.00	91.00	88.25	89.57
	4,5	85.57	86.28	86.03	86.04	85.90	86.37
(c)	1,2	85.56	86.00	89.07	88.60	88.21	88.91
	3	85.64	88.83	88.08	90.99	88.89	89.87
	4,5	85.57	86.28	85.89	85.52	86.15	86.24
(d)	1,2,3	85.58	86.65	88.11	88.69	88.91	88.59
	4,5	85.57	86.28	86.23	84.17	86.02	86.10
		**Maximum Type I Error Rate**					
**Sub-Case**	**Basket(s)**	Independent	EXNEX	$\text{EXNEX}_{\text{pool}}$	$\text{mEXNEX}_{\text{hist}}$	EXppNEX	MLMixture
(a)	1,2,3	10.18	12.32	13.66	16.02	12.08	12.26
	4,5	10.48	14.48	17.64	22.08	12.42	18.34
(b)	1	9.92	7.08	11.88	11.62	11.58	11.26
	2,3	10.18	12.32	12.72	13.78	11.76	12.52
	4,5	10.48	14.48	15.84	17.04	13.04	18.58
(c)	1,2	10.18	12.32	13.04	15.24	12.40	13.22
	3	10.16	11.44	12.04	13.76	11.76	12.64
	4,5	10.48	14.48	14.10	16.28	13.70	19.78
(d)	1,2,3	10.18	12.32	12.42	16.34	12.26	12.68
	4,5	10.48	14.48	11.84	22.00	14.10	18.16

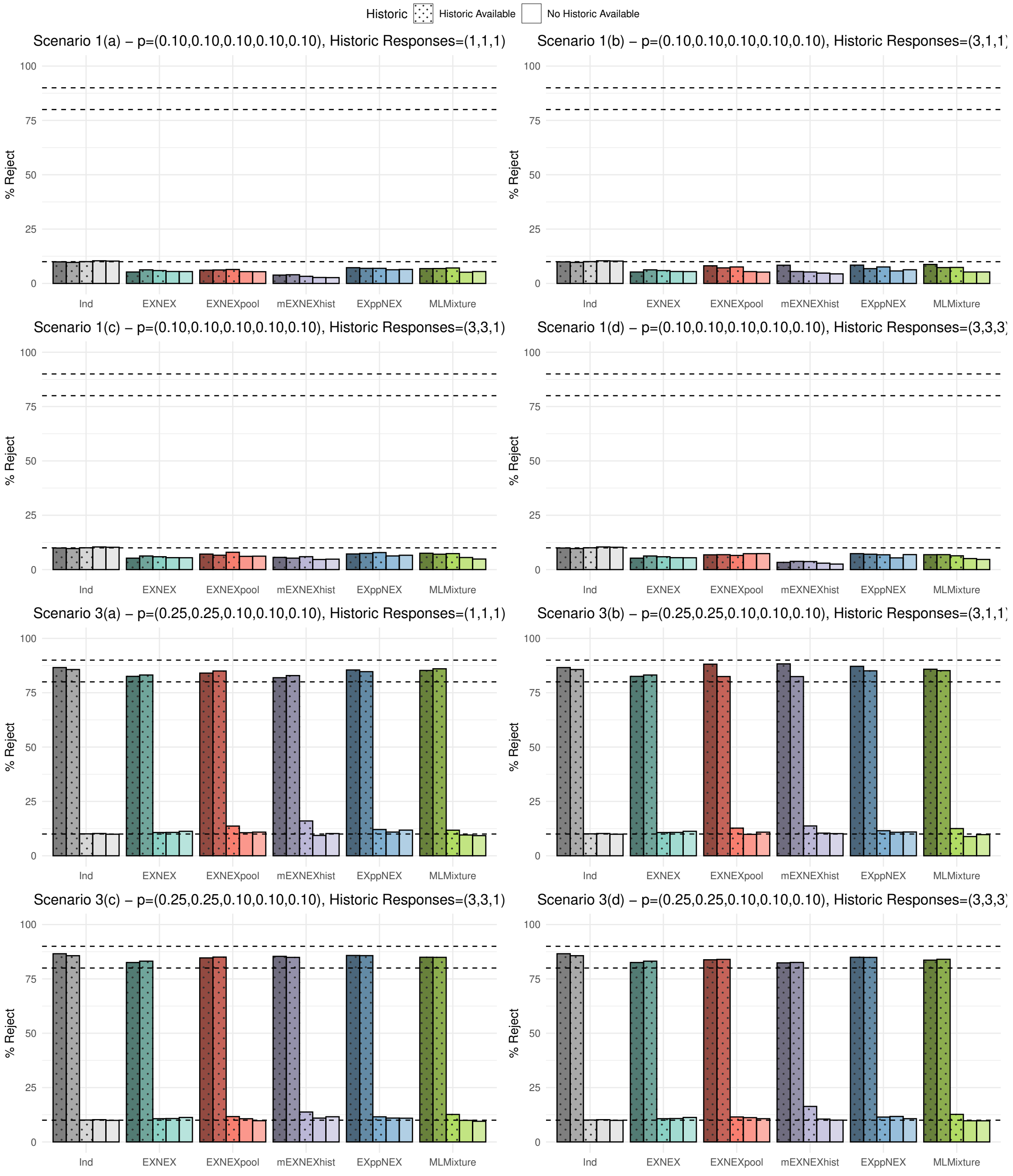

Fig. 1: Simulation results: type I error rate and power under each of the 8 approaches for scenarios 1 and 3 cases (a)-(d).

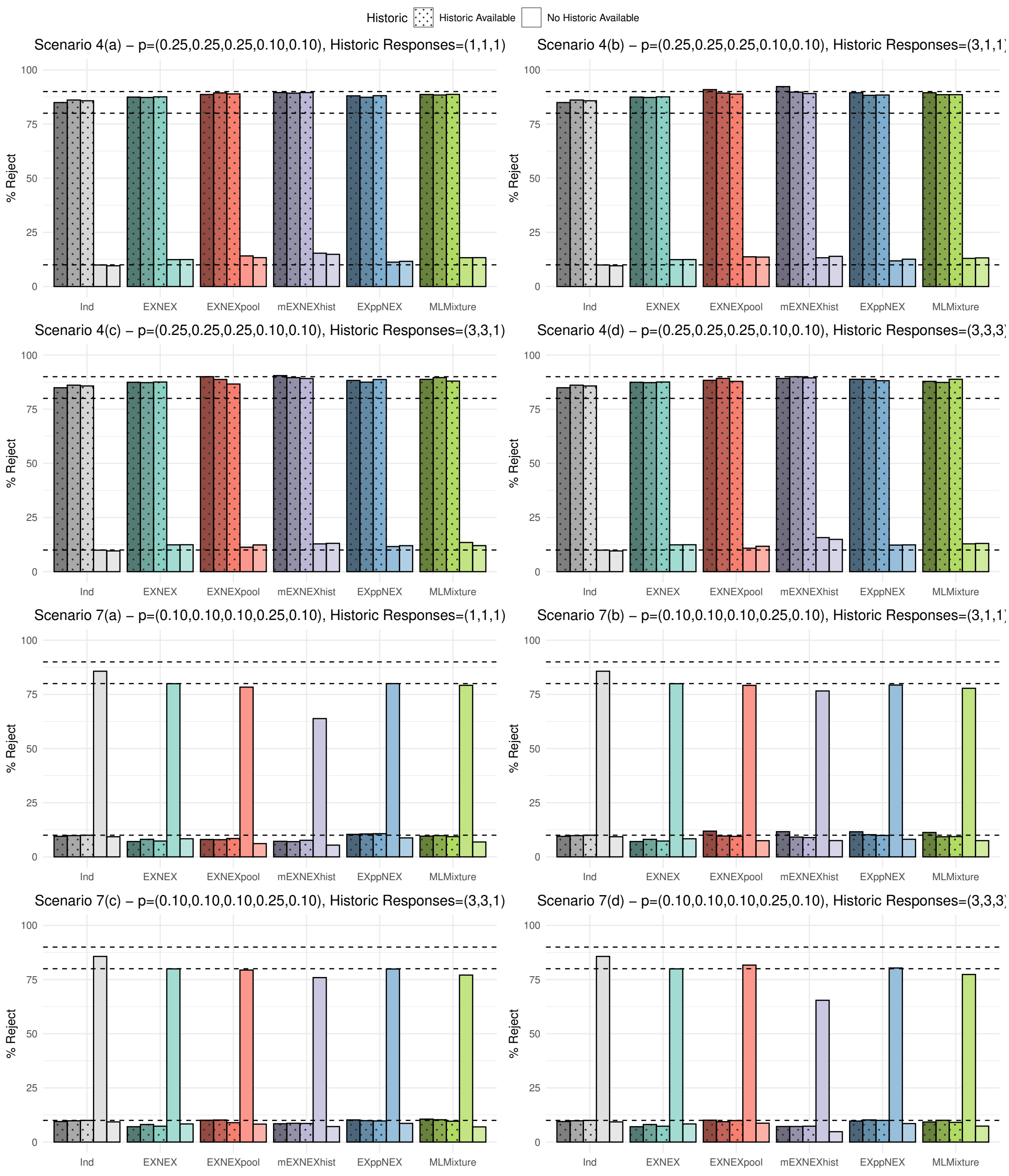

Fig. 2: Simulation results: type I error rate and power under each of the 8 approaches for scenarios 4 and 7 cases (a)-(d).

E. Competing Approaches

E.1 *Adapted Fujikawa's Design (histFujikawa)*

For comparison within the simulation study, an alternative empirical approach is also considered. Fujikawa *and others* (2020) developed a closed-form Beta posterior for each basket, within which the parameters incorporate the response data of other baskets in the trial in order to borrow information. Fujikawa's design first fits independent Beta-Binomial models to the response rates, p_k, with a Beta(a_k, b_k) prior used. This results in the following posterior: $\pi(p_k|Y_k = y_k) =$ Beta$(a_k + y_k, b_k + n_k - y_k)$ for basket k. Fujikawa proposed borrowing of information by taking the weighted sum of these posterior parameters with weight, $\omega_{k,i}$ representing the degree of homogeneity between baskets k and i. The posterior of the response rate given observed data D for all baskets is then

$$\pi(p_k|D) = \text{Beta}\left(\sum_{i=1}^{K} \mathbb{I}(\omega_{k,i}^{\epsilon} > \tau)\omega_{k,i}^{\epsilon}(a_k + y_i), \sum_{i=1}^{K} \mathbb{I}(\omega_{k,i}^{\epsilon} > \tau)\omega_{k,i}^{\epsilon}(b_k + n_i - y_i)\right), \quad \text{(E.1)}$$

where $\epsilon \geqslant 1$ and $\tau \in [0, 1]$ are tuning parameters. ϵ controls how quickly the weights move to 0 to discourage borrowing as the baskets response rates become increasingly heterogeneous to one another, whilst τ acts as a cut-off which sets the weights to 0 should heterogeneity cross the threshold, τ.

Fujikawa suggested setting the weights $\omega_{k,i}$ based on 1 minus the pair-wise Jensen-Shannon divergence (JSD) between the Beta-Binomial posteriors for baskets k and i:

$$\omega_{k,i} = 1 - \text{JSD}(\pi(p_k|Y_k = y_k), \pi(p_i|Y_i = y_i)), \quad \text{(E.2)}$$

where the JSD between two distributions P and Q is $\text{JSD}(P, Q) = 1/2(KL(P||M) + DL(Q||M))$ with $M = 1/2(P + Q)$ (Fuglede and Topsoe, 2004). $KL(P||M) = \sum_x P(x)\log(P(x)/M(x))$ is the Kullback-Leibler Divergence (KLD). In order to obtain weights bounded between 0 and 1, KLD is used with base 2 logarithm (Baumann *and others*, 2023).

A similar approach can be used to also incorporate historical information, with the parameters of the Beta posterior now including both a weighted sum of current data and a weighted sum of historic data, with weights determined as a function of homogeneity between baskets:

$$\pi(p_k|D, D_h) = \text{Beta}\Bigg(a_k + \sum_{i=1}^{K} \Bigg(\mathbb{I}\{\omega^{\epsilon}_{k,i} > \tau\}\omega^{\epsilon}_{k,i} y_i + \zeta_k \sum_{j=1}^{H_k} \Big(\mathbb{I}\{\omega^{\epsilon}_{k,i^*(j)} > \tau\}\omega^{*\epsilon}_{k,i^*(j)} y_{i^*(j)} \Big) \Bigg), \\ b_k + \sum_{i=1}^{K} \Bigg(\mathbb{I}\{\omega^{\epsilon}_{k,i} > \tau\}\omega^{\epsilon}_{k,i}(n_i - y_i) + \zeta_k \sum_{j=1}^{H_k} \Big(\mathbb{I}\{\omega^{*\epsilon}_{k,i^*(j)} > \tau\}\omega^{*\epsilon}_{k,i^*(j)} \left(n_{i^*(j)} - y_{i^*(j)}\right) \Big) \Bigg) \Bigg), \tag{E.3}$$

where weights between current data sources, $\omega_{k,i}$ are computed as in (E.2) using the JSD. Weights between a current basket k and a historic basket associated with basket i from one of the H_i previous studies are also computed using JSD but are set to 0 should no historic information be available for basket i:

$$\omega_{k,i^*(j)} = \begin{cases} 1 - JSD\left(\pi(p_k|Y_k = y_k), \pi(p_{i^*(j)}|Y_{i^*(j)} = y_{i^*(j)})\right) & \text{if historic information is available for basket } i, \\ 0 & \text{otherwise.} \end{cases}$$

Note that unlike in Fujikawa's design, information is not shared between the prior distributions, with the prior parameters moved outside of the sum. The tuning parameters ϵ and τ are still defined in the same way as in Fujikawa's design, with ϵ defining the degree of decline of weights with heterogeneity and τ as a cut-off for borrowing when the degree of heterogeneity becomes too large. This approach has a computational advantage over the other methods proposed due to the closed form solution of posteriors it provides.

E.1.1 *Simulation Study Model Specification* Within the simulation results presented in this Supplementary Materials, results of the histFujikawa's design are also presented. The simulation setting is the same as that in the main text, with 5 current baskets and historic information available for the first 3. The model applied is:

$$\pi(p_k|D, D_h) = \text{Beta}\Bigg(1+\sum_{i=1}^{5}(\mathbb{I}\{\omega^{\epsilon}_{k,i} > \tau\}\omega^{\epsilon}_{k,i}y_i + 0.8\mathbb{I}\{\omega^{*\epsilon}_{k,i^*} > \tau\}\omega^{*\epsilon}_{k,i^*}y_{i^*}),$$
$$1+\sum_{i=1}^{5}(\mathbb{I}\{\omega^{\epsilon}_{k,i} > \tau\}\omega^{\epsilon}_{k,i}(n_i - y_i) + 0.8\mathbb{I}\{\omega^{*\epsilon}_{k,i^*} > \tau\}\omega^{*\epsilon}_{k,i^*}(n_{i^*} - y_{i^*}))\Bigg)$$

where $\omega_{k,i} = 1 - \text{JSD}(\pi(p_k|Y_k = y_k), \pi(p_i|Y_i = y_i))$ and

$$\omega_{k,i^{*(j)}} = \begin{cases} 1 - JSD\left(\pi(p_k|Y_k = y_k), \pi(p^{(j)}_{i^*}|Y^{(j)}_{i^*} = y^{(j)}_{i^*})\right) & \text{if historic information is available for basket } i, \\ 0 & \text{otherwise.} \end{cases}$$

with $\epsilon = 2$ and $\tau = 0.2$.

E.2 *EXNEX With SAM Prior in the NEX Component (EXsamNEX)*

As described in the main text, selecting the power α in the power prior can be challenging and significantly effect inference. In the EXppNEX approach this power prior was placed on the NEX component in the EXNEX model. To avoid the specification of the power α, the power prior can be replaced with a self-adaptive mixture (SAM) prior:

$$\begin{aligned}
&Y_k \sim \text{Binomial}(n_k, p_k), \quad k = 1, \ldots, K && \theta_k = \text{logit}(M_{1k}) \sim \text{N}(\mu, \sigma^2),\\
&p_k = \delta_k M_{1k} + (1 - \delta_k)M_{2k} && \mu \sim \text{N}(m_\mu, \nu_\mu),\\
&\mathbb{I}_k = 1 \text{ if } y^{(j)}_{k^*} \text{ exists for basket } k \text{ for some } j \geqslant 1, && \sigma \sim g(\cdot),\\
&\delta_k \sim \text{Bernoulli}(\pi_k), && \tilde{\omega}_k \sim \text{Bernoulli}(\phi_k),\\
& && M_{2k} = \mathbb{I}_k\tilde{\omega}_k\pi_1(p_k) + (1 - \tilde{\omega}_k)\pi_0(p_k).
\end{aligned} \tag{E.4}$$

Note that the parameters for the EX component remain unchanged compared to the EXNEX and EXppNEX models. The SAM prior is placed on the NEX, M_{2k}, component consisting of a mixture of an informative prior, π_1, and uninformative prior, π_0. The non-informative prior is simply $\pi_0(p_k) = \text{Beta}(a_k, b_k)$ with values $a_k = b_k = 1$ recommended. The informative prior is a Meta-analytic predictive prior (Weber *and others*, 2021). The MAP prior in not tractable and thus MCMC methods would need to be utilized, however, it is approximated by a mixture of

conjugate priors (Schmidli *and others*, 2014):

$$\pi_1(p_k) = \sum_{i=1}^{H_k} \kappa_i \text{Beta}(a_k + y_{k^*}^{(i)}, b_k + n_{k^*}^{(i)} - y_{k^*}^{(i)}),$$

where the κ_i weights are positive and sum to one. Weights can be defined as fixed in the model or can be updated in the posterior. Should there be a single source of historic data, this weight is set at $\kappa_k = 1$ thus $\pi_1(p_k) = \text{Beta}(a_k + y_{k^*}, b_k + n_{k^*} - y_{k^*})$.

The SAM prior mixture weights follow a Bernoulli distribution with probability ϕ_k, where ϕ_k is computed as guided by Yang *and others* (2023), utilizing the likelihood ratio test statistic. Let $\hat{p}_{k^*} = \int p_k \pi_1(p_k) dp_k$ be the expected value of p_k based on π_1. In cases of a single source of historic data in a basket $\hat{p}_{k^*} = (a_k + y_{k^*})/(a_k + b_k + n_{k^*})$ is the estimate of p_{k^*}, so the likelihood ratio test statistic is then:

$$R_k = \frac{\hat{p}_{k^*}^{y_k}(1-\hat{p}_{k^*})^{n_k - y_k}}{\max\{(\hat{p}_{k^*}+\Omega)^{y_k}(1-\hat{p}_{k^*}-\Omega)^{n_k-y_k}, (\hat{p}_{k^*}-\Omega)^{y_k}(1-\hat{p}_{k^*}+\Omega)^{n_k-y_k}\}}, \tag{E.5}$$

where ϕ_k is then set as $\phi_k = R_k/(1 + R_k)$.

E.2.1 *Simulation Study Model Specification* Within the simulation results presented in this Supplementary Materials, results of the EXsamNEX are also presented. The simulation setting is the same as that in the main text, with 5 current baskets and historic information available for the first 3. The model applied is:

$$\begin{aligned}
&Y_k \sim \text{Binomial}(n_k, p_k), \qquad k = 1,2,3,4,5, && \theta_k = \text{logit}(M_{1k}) \sim \text{N}(\mu, \sigma^2),\\
&p_k = \delta_k M_{1k} + (1-\delta_k) M_{2k}, && \mu \sim \text{N}(\text{logit}(0.1), 10^2),\\
&\mathbb{I}_k = 1 \text{ if } y_{k^*} \text{ exists for basket } k, && \sigma \sim \text{Half-Normal}(0,1),\\
&\delta_k \sim \text{Bernoulli}(\pi_k), && \tilde{\omega}_k \sim \text{Bernoulli}(\phi_k),\\
& && M_{2k} = \mathbb{I}_k \tilde{\omega}_k \pi_1(p_k) + (1-\tilde{\omega}_k)\pi_0(p_k),\\
& && \pi_1(p_k) = \text{Beta}(1 + y_{k^*}, 1 + n_{k^*} - y_{k^*}),\\
& && \pi_0(p_k) = \text{Beta}(1,1),
\end{aligned}$$

where $\pi_k = 0.5$ for $k = 1, 2, 3, 4, 5$. Prior probabilities, ϕ_k are computed as follows: let $\hat{p}_{k^*} = (1 + y_{k^*})/(1 + 1 + n_{k^*})$ and

$$R_k = \frac{\hat{p}_{k^*}^{y_k}(1 - \hat{p}_{k^*})^{n_k - y_k}}{\max\{(\hat{p_k^*} + 0.15)^{y_k}(1 - \hat{p}_{k^*} - 0.15)^{n_k - y_k}, (\hat{p}_{k^*} - 0.15)^{y_k}(1 - \hat{p}_{k^*} + 0.15)^{n_k - y_k}\}},$$

where 0.15 is the clinically relevant difference. $\phi_k = R_k/(1 + R_k)$.

F. Simulation Results

Table 4. *Simulation Results for scenario 1 under historic cases (a), (b), (c) and (d).*

	y_{k^*}	% Reject					FWER	% All Correct
**Scenario 1**		**0.1**	**0.1**	**0.1**	**0.1**	**0.1**		
**(a)**	**(1,1,1)**							
Independent		9.92	9.68	10.04	10.42	10.28	41.00	59.00
EXNEX		5.26	6.26	5.92	5.50	5.46	22.66	77.34
$\text{EXNEX}_{\text{pool}}$		6.10	6.20	6.42	5.44	5.40	23.12	76.88
$\text{mEXNEX}_{\text{hist}}$		3.84	4.00	3.26	2.74	2.68	11.44	88.56
histFujikawa		4.42	4.08	4.42	1.90	2.04	10.66	89.34
EXppNEX		7.24	6.94	6.94	.30	6.46	25.72	74.28
EXsamNEX		7.70	7.48	7.34	5.82	5.80	26.96	73.04
MLMixture		6.82	6.86	7.12	5.16	5.48	24.70	75.30
**(b)**	**(3,1,1)**							
Independent		9.92	9.68	10.04	10.42	10.28	41.00	59.00
EXNEX		5.26	6.26	5.92	5.50	5.46	22.66	77.34
$\text{EXNEX}_{\text{pool}}$		8.12	7.16	7.58	5.46	5.22	25.36	74.46
$\text{mEXNEX}_{\text{hist}}$		8.38	5.48	5.28	4.74	4.40	21.08	78.92
histFujikawa		7.02	4.42	4.08	2.16	2.32	12.64	87.36
EXppNEX		8.42	6.80	7.58	5.74	6.30	27.10	72.90
EXsamNEX		7.96	7.44	7.82	5.10	5.46	26.18	73.82
MLMixture		8.74	7.30	7.36	5.26	5.28	26.12	73.88
**(c)**	**(3,3,1)**							
Independent		9.92	9.68	10.04	10.42	10.28	41.00	59.00
EXNEX		5.26	6.26	5.92	5.50	5.46	22.66	77.34
$\text{EXNEX}_{\text{pool}}$		7.14	6.62	7.98	6.10	6.18	25.98	74.02
$\text{mEXNEX}_{\text{hist}}$		5.62	5.28	5.94	4.66	4.80	19.52	80.48
histFujikawa		4.50	4.96	4.66	2.84	2.86	13.00	87.00
EXppNEX		7.20	7.44	7.84	6.28	6.60	26.98	73.02
EXsamNEX		7.98	7.42	7.10	5.28	5.50	26.32	73.68
MLMixture		7.52	7.08	7.36	5.56	4.86	25.14	74.86
**(d)**	**(3,3,3)**							
Independent		9.92	9.68	10.04	10.42	10.28	41.00	59.00
EXNEX		5.26	6.26	5.92	5.50	5.46	22.66	77.34
$\text{EXNEX}_{\text{pool}}$		6.80	6.86	6.52	7.32	7.36	26.24	73.76
$\text{mEXNEX}_{\text{hist}}$		3.32	3.80	3.70	2.96	2.54	10.96	89.04
histFujikawa		4.94	4.66	4.68	3.04	2.62	13.82	86.18
EXppNEX		7.34	7.08	6.82	5.40	6.90	25.78	74.22
EXsamNEX		6.42	6.94	7.18	5.78	6.26	24.98	75.02
MLMixture		6.86	6.88	6.36	5.08	4.68	23.36	76.64

Table 5. *Simulation Results for scenario 2 under historic cases (a), (b), (c) and (d).*

	y_{k^*}	% Reject					FWER	% All Correct
**Scenario 2**		**0.25**	**0.1**	**0.1**	**0.1**	**0.1**		
**(a)**	**(1,1,1)**							
Independent		84.90	9.32	10.16	10.10	10.48	34.78	55.46
EXNEX		78.86	7.68	7.88	7.92	7.92	24.02	58.40
$\text{EXNEX}_{\text{pool}}$		80.30	9.28	9.48	6.36	6.86	24.36	58.72
$\text{mEXNEX}_{\text{hist}}$		66.92	8.84	9.12	6.30	5.80	21.8	47.04
histFujikawa		70.24	8.20	8.84	5.08	5.54	19.64	51.90
EXppNEX		81.24	9.52	9.68	8.32	7.96	27.78	56.92
EXsamNEX		83.00	10.32	9.48	6.88	7.02	26.96	58.82
MLMixture		81.12	9.42	9.52	6.02	6.68	25.42	58.88
**(b)**	**(3,1,1)**							
Independent		84.90	9.32	10.16	10.10	10.48	34.78	55.46
EXNEX		78.86	7.68	7.88	7.92	7.92	24.02	58.40
$\text{EXNEX}_{\text{pool}}$		81.44	8.90	8.50	6.86	6.66	23.70	60.72
$\text{mEXNEX}_{\text{hist}}$		82.96	8.02	8.32	6.70	6.80	22.18	62.74
histFujikawa		76.16	8.34	7.88	5.94	5.78	19.28	57.60
EXppNEX		83.68	9.66	9.44	8.10	7.70	27.46	59.36
EXsamNEX		82.76	9.24	8.82	7.40	6.74	25.96	59.54
MLMixture		83.06	9.30	9.04	6.90	6.56	25.40	60.74
**(c)**	**(3,3,1)**							
Independent		84.90	9.32	10.16	10.10	10.48	34.78	55.46
EXNEX		78.66	7.68	7.88	7.92	7.92	24.02	58.40
$\text{EXNEX}_{\text{pool}}$		81.94	10.16	9.38	7.52	8.22	27.18	57.88
$\text{mEXNEX}_{\text{hist}}$		77.62	10.82	9.28	8.46	7.44	27.50	52.90
histFujikawa		72.42	10.02	8.50	5.10	5.84	21.42	52.40
EXppNEX		81.76	10.28	9.10	8.10	7.56	28.28	56.40
EXsamNEX		82.08	9.18	10.26	6.70	6.46	26.32	58.80
MLMixture		82.02	9.40	8.72	6.90	6.92	24.86	60.04
**(d)**	**(3,3,3)**							
Independent		84.90	9.32	10.16	10.10	10.48	34.78	55.46
EXNEX		78.86	7.68	7.88	7.92	7.92	24.02	58.40
$\text{EXNEX}_{\text{pool}}$		81.06	8.98	9.64	9.18	8.92	28.94	56.14
$\text{mEXNEX}_{\text{hist}}$		68.06	9.38	8.48	5.80	6.48	21.60	48.44
histFujikawa		71.74	8.40	8.90	5.84	5.44	20.52	52.76
EXppNEX		81.70	9.68	9.48	8.88	7.68	28.18	56.78
EXsamNEX		80.84	9.22	8.82	6.74	6.80	24.74	59.52
MLMixture		81.10	8.60	9.26	6.54	7.10	24.96	59.54

Table 6. *Simulation Results for scenario 3 under historic cases (a), (b), (c) and (d).*

	y_{k^*}	% Reject					FWER	% All Correct
**Scenario 3**		**0.25**	**0.25**	**0.1**	**0.1**	**0.1**		
**(a)**	**(1,1,1)**							
Independent		86.58	85.68	10.08	10.24	9.92	27.24	54.24
EXNEX		82.54	83.16	10.70	10.76	11.26	28.66	45.40
$\text{EXNEX}_{\text{pool}}$		84.04	85.00	13.66	10.62	10.88	28.90	47.82
$\text{mEXNEX}_{\text{hist}}$		81.92	82.90	16.02	9.36	10.22	27.38	48.04
histFujikawa		81.24	81.04	13.88	9.76	10.68	26.12	45.66
EXppNEX		85.48	84.7	12.08	10.90	11.80	30.02	48.64
EXsamNEX		84.96	84.70	11.90	10.34	9.44	27.22	50.68
MLMixture		85.26	86.02	11.76	9.56	9.26	25.46	52.62
**(b)**	**(3,1,1)**							
Independent		86.58	85.68	10.08	10.24	9.92	27.24	54.24
EXNEX		82.54	83.16	10.70	10.76	11.26	28.66	45.40
$\text{EXNEX}_{\text{pool}}$		88.14	82.46	12.72	9.86	10.86	28.32	49.64
$\text{mEXNEX}_{\text{hist}}$		88.28	82.44	13.72	10.42	10.20	27.88	49.88
histFujikawa		86.02	81.58	14.32	11.18	10.28	27.32	47.96
EXppNEX		87.14	85.08	11.52	10.82	10.94	29.26	50.82
EXsamNEX		86.30	85.26	12.00	9.96	9.66	27.04	51.54
MLMixture		85.82	85.18	12.52	8.84	9.74	25.46	52.62
**(c)**	**(3,3,1)**							
Independent		86.58	85.68	10.08	10.24	9.92	27.24	54.24
EXNEX		82.54	83.16	10.70	10.76	11.26	28.66	45.40
$\text{EXNEX}_{\text{pool}}$		84.66	85.04	11.66	10.68	9.76	27.92	49.66
$\text{mEXNEX}_{\text{hist}}$		85.38	84.90	13.76	10.96	11.58	30.26	48.56
histFujikawa		82.74	82.70	13.02	10.14	9.74	25.34	48.08
EXppNEX		85.78	85.72	11.56	10.98	10.92	29.62	50.42
EXsamNEX		85.82	85.74	11.64	9.16	9.76	26.60	52.56
MLMixture		84.96	84.92	12.64	9.92	9.50	26.26	50.36
**(d)**	**(3,3,3)**							
Independent		86.58	85.68	10.08	10.24	9.92	27.24	54.24
EXNEX		82.54	83.16	10.70	10.76	11.26	28.66	45.40
$\text{EXNEX}_{\text{pool}}$		83.80	83.98	11.50	11.18	10.70	28.74	47.76
$\text{mEXNEX}_{\text{hist}}$		82.38	82.58	16.34	10.52	10.04	28.88	46.72
histFujikawa		82.56	82.12	13.16	10.00	10.30	25.46	47.66
EXppNEX		84.94	84.90	11.44	11.72	10.70	28.42	50.10
EXsamNEX		83.18	83.82	11.78	8.70	9.56	25.04	49.80
MLMixture		83.64	84.04	12.68	9.74	9.72	25.78	49.78

Table 7. *Simulation Results for scenario 4 under historic cases (a), (b), (c) and (d).*

	y_{k^*}	% Reject					FWER	% All Correct
**Scenario 4**		**0.25**	**0.25**	**0.25**	**0.1**	**0.1**		
**(a)**	**(1,1,1)**							
Independent		84.92	86.10	85.74	9.94	9.58	18.74	51.70
EXNEX		87.44	87.28	87.56	12.40	12.44	22.20	52.94
$\text{EXNEX}_{\text{pool}}$		88.64	89.38	88.92	14.12	13.34	23.56	53.98
$\text{mEXNEX}_{\text{hist}}$		89.52	89.26	89.52	15.38	14.86	25.26	52.86
histFujikawa		88.10	88.60	89.10	16.02	15.76	26.86	48.44
EXppNEX		88.02	87.36	88.08	11.26	11.62	21.36	54.08
EXsamNEX		88.34	88.18	88.28	12.36	12.28	21.44	54.38
MLMixture		88.64	88.42	88.68	13.30	13.34	22.86	53.66
**(b)**	**(3,1,1)**							
Independent		84.92	86.10	85.74	9.94	9.58	18.74	51.70
EXNEX		87.44	87.28	87.56	12.40	12.44	22.20	52.94
$\text{EXNEX}_{\text{pool}}$		90.90	89.30	88.84	13.72	13.58	23.52	55.04
$\text{mEXNEX}_{\text{hist}}$		92.30	89.76	89.08	13.28	13.94	23.70	56.26
histFujikawa		90.54	88.14	87.80	15.80	14.82	26.14	50.00
EXppNEX		89.46	88.26	88.40	11.84	12.60	23.00	53.90
EXsamNEX		88.30	87.84	88.18	12.78	12.92	22.24	53.14
MLMixture		89.46	88.56	88.56	12.98	13.22	21.80	54.08
**(c)**	**(3,3,1)**							
Independent		84.92	86.10	85.74	9.94	9.58	18.74	51.70
EXNEX		87.44	87.28	87.56	12.40	12.44	22.20	52.94
$\text{EXNEX}_{\text{pool}}$		90.02	88.74	86.64	11.32	12.36	21.58	54.00
$\text{mEXNEX}_{\text{hist}}$		90.52	89.58	89.16	12.84	13.08	22.92	54.74
histFujikawa		88.36	89.38	88.90	16.22	15.44	26.62	49.62
EXppNEX		88.28	87.50	88.70	11.60	12.00	21.46	53.94
EXsamNEX		88.58	88.24	88.94	13.14	12.68	22.34	53.68
MLMixture		88.78	89.60	87.98	13.48	12.02	22.08	53.96
**(d)**	**(3,3,3)**							
Independent		84.92	86.10	85.74	9.94	9.58	18.74	51.70
EXNEX		87.44	87.28	87.56	12.40	12.44	22.20	52.94
$\text{EXNEX}_{\text{pool}}$		88.34	89.24	87.86	10.84	11.74	21.20	55.28
$\text{mEXNEX}_{\text{hist}}$		89.20	90.00	89.46	15.74	14.92	25.86	51.82
histFujikawa		88.50	88.32	88.26	15.58	15.68	26.62	48.50
EXppNEX		88.82	87.62	87.70	12.22	12.26	21.14	54.02
EXsamNEX		88.80	87.62	87.70	12.22	12.26	21.14	54.02
MLMixture		87.86	87.38	88.84	12.86	13.04	21.92	53.14

Table 8. *Simulation Results for scenario 5 under historic cases (a), (b), (c) and (d).*

	y_{k^*}	% Reject					FWER	% All Correct
**Scenario 5**		**0.25**	**0.25**	**0.25**	**0.25**	**0.1**		
**(a)**	**(1,1,1)**							
Independent		85.42	85.86	85.14	85.48	10.26	10.26	47.52
EXNEX		88.48	89.48	88.86	88.64	14.48	14.48	52.34
$\text{EXNEX}_{\text{pool}}$		89.58	89.60	91.24	89.24	17.64	17.64	53.60
$\text{mEXNEX}_{\text{hist}}$		94.10	93.40	93.50	90.16	22.08	22.08	56.82
histFujikawa		91.68	91.68	92.16	88.16	20.46	20.46	54.18
EXppNEX		88.90	88.94	89.40	88.48	12.42	12.42	54.14
EXsamNEX		88.24	88.82	88.66	88.28	17.40	17.40	49.02
MLMixture		90.20	89.72	89.72	89.16	18.34	18.34	50.40
**(b)**	**(3,1,1)**							
Independent		85.42	85.86	85.14	85.48	10.26	10.26	47.52
EXNEX		88.48	89.48	88.86	88.64	14.48	14.48	52.34
$\text{EXNEX}_{\text{pool}}$		94.32	90.18	90.46	89.24	15.84	15.84	56.88
$\text{mEXNEX}_{\text{hist}}$		94.42	92.36	93.24	89.60	17.04	17.04	60.56
histFujikawa		93.06	91.96	91.60	88.26	20.16	20.16	54.72
EXppNEX		91.74	89.08	88.96	89.44	13.04	13.04	56.86
EXsamNEX		90.96	87.96	88.90	88.54	17.22	17.22	50.30
MLMixture		92.44	89.76	89.62	88.92	18.58	18.58	51.68
**(c)**	**(3,3,1)**							
Independent		85.42	85.86	85.14	85.48	10.26	10.26	47.52
EXNEX		88.48	89.48	88.86	88.64	14.48	14.48	52.34
$\text{EXNEX}_{\text{pool}}$		92.16	92.15	88.42	88.78	14.10	14.10	57.60
$\text{mEXNEX}_{\text{hist}}$		93.00	92.78	90.68	89.48	16.28	16.28	58.14
histFujikawa		92.30	92.40	91.300	89.78	19.10	19.10	57.00
EXppNEX		89.54	89.52	89.20	88.50	13.70	13.70	54.00
EXsamNEX		90.36	90.84	88.90	88.50	17.94	17.94	50.80
MLMixture		91.88	91.94	89.82	89.18	19.78	19.78	51.94
**(d)**	**(3,3,3)**							
Independent		85.42	85.86	85.14	85.48	10.26	10.26	47.52
EXNEX		88.48	89.48	88.86	88.64	14.48	14.48	52.34
$\text{EXNEX}_{\text{pool}}$		89.70	89.74	89.96	89.18	11.84	11.84	56.20
$\text{mEXNEX}_{\text{hist}}$		94.12	93.82	94.20	90.64	22.20	22.20	57.86
histFujikawa		91.68	92.08	90.96	88.74	19.66	19.66	54.66
EXppNEX		89.94	90.90	90.32	88.46	14.10	14.10	54.76
EXsamNEX		90.56	89.48	90.56	88.26	17.54	17.54	51.38
MLMixture		91.09	90.02	89.78	89.16	18.16	18.16	51.60

Table 9. *Simulation Results for scenario 6 under historic cases (a), (b), (c) and (d).*

	y_{k^*}	% Reject					FWER	% All Correct
**Scenario 6**		**0.25**	**0.25**	**0.25**	**0.25**	**0.25**		
**(a)**	**(1,1,1)**							
Independent		85.26	85.12	86.04	86.04	85.34		45.98
EXNEX		90.16	89.82	90.06	90.28	90.10		61.10
$\text{EXNEX}_{\text{pool}}$		93.52	93.34	92.32	91.16	91.38		69.50
$\text{mEXNEX}_{\text{hist}}$		96.54	96.92	96.58	93.46	93.40		72.42
histFujikawa		94.24	94.54	94.78	92.62	92.84		75.60
EXppNEX		87.56	88.54	88.62	88.64	89.10		54.56
EXsamNEX		89.70	88.42	88.30	91.40	91.72		60.94
MLMixture		92.40	92.36	92.28	91.70	91.88		69.98
**(b)**	**(3,1,1)**							
Independent		85.26	85.12	86.04	86.04	85.34		45.98
EXNEX		90.16	89.82	90.06	90.28	90.10		61.10
$\text{EXNEX}_{\text{pool}}$		94.82	91.04	90.74	90.04	90.60		65.34
$\text{mEXNEX}_{\text{hist}}$		95.28	94.68	95.46	91.90	9.146		73.60
histFujikawa		95.14	94.22	94.44	92.18	92.64		75.38
EXppNEX		94.88	89.36	88.58	89.04	88.44		59.60
EXsamNEX		94.16	89.70	88.28	90.96	91.88		64.98
MLMixture		94.96	92.66	92.68	92.58	92.48		72.62
**(c)**	**(3,3,1)**							
Independent		85.26	85.12	86.04	86.04	85.34		45.98
EXNEX		90.16	89.82	90.06	90.28	90.10		61.10
$\text{EXNEX}_{\text{pool}}$		94.44	94.62	89.18	90.26	89.18		65.20
$\text{mEXNEX}_{\text{hist}}$		94.92	94.30	93.14	90.26	90.44		69.86
histFujikawa		94.94	94.70	94.38	93.00	91.96		75.90
EXppNEX		93.86	93.96	88.76	89.58	89.28		64.82
EXsamNEX		94.44	93.86	88.90	91.86	91.48		68.70
MLMixture		94.28	94.86	91.80	92.48	91.66		72.80
**(d)**	**(3,3,3)**							
Independent		85.26	85.12	86.04	86.04	85.34		45.98
EXNEX		90.16	89.82	90.06	90.28	90.10		61.10
$\text{EXNEX}_{\text{pool}}$		92.44	92.10	92.52	87.88	88.46		62.86
$\text{mEXNEX}_{\text{hist}}$		96.52	96.58	96.98	96.36	93.84		81.90
histFujikawa		93.70	94.56	94.70	92.80	92.26		75.42
EXppNEX		93.52	93.90	93.74	89.72	89.48		69.52
EXsamNEX		93.38	93.80	93.14	91.62	90.90		71.68
MLMixture		94.30	94.50	94.38	91.52	92.00		73.84

Table 10. *Simulation Results for scenario 7 under historic cases (a), (b), (c) and (d).*

	y_{k^*}	% Reject					FWER	% All Correct
**Scenario 7**		**0.1**	**0.1**	**0.1**	**0.25**	**0.1**		
**(a)**	**(1,1,1)**							
Independent		9.48	9.82	10.00	85.70	9.30	33.10	57.22
EXNEX		7.08	8.10	7.32	79.94	8.36	24.00	59.56
$\text{EXNEX}_{\text{pool}}$		8.04	7.94	8.48	78.36	6.12	23.54	58.66
$\text{mEXNEX}_{\text{hist}}$		7.18	7.04	7.66	63.84	5.42	18.90	48.12
histFujikawa		9.18	9.44	9.28	58.74	5.46	22.28	39.98
EXppNEX		10.40	10.58	10.70	80.00	8.76	32.58	51.68
EXsamNEX		10.84	10.76	10.24	77.74	7.40	32.12	51.40
MLMixture		9.58	9.80	9.34	79.20	6.88	28.06	55.80
**(b)**	**(3,1,1)**							
Independent		9.48	9.82	10.00	85.70	9.30	33.10	57.22
EXNEX		7.08	8.10	7.32	79.94	8.36	24.00	59.56
$\text{EXNEX}_{\text{pool}}$		11.88	9.64	9.46	79.18	7.48	29.92	53.88
$\text{mEXNEX}_{\text{hist}}$		11.62	9.14	9.74	78.90	7.24	27.24	53.00
histFujikawa		12.90	9.54	9.34	61.40	6.24	25.56	40.42
EXppNEX		11.58	10.24	9.92	79.32	8.12	32.80	51.42
EXsamNEX		12.04	10.76	9.54	79.64	7.54	32.04	52.34
MLMixture		11.26	9.28	9.36	77.82	7.50	29.18	53.48
**(c)**	**(3,3,1)**							
Independent		9.48	9.82	10.00	85.70	9.30	33.10	57.22
EXNEX		7.08	8.10	7.32	79.94	8.36	24.00	59.56
$\text{EXNEX}_{\text{pool}}$		10.08	10.18	8.94	79.40	8.28	29.54	53.58
$\text{mEXNEX}_{\text{hist}}$		8.46	8.66	8.58	75.92	7.16	24.36	54.94
histFujikawa		9.70	9.80	9.36	65.42	5.96	23.86	45.02
EXppNEX		10.22	9.88	9.74	79.88	8.62	30.90	53.08
EXsamNEX		10.70	10.76	9.60	79.60	7.12	30.24	54.04
MLMixture		10.58	10.30	9.62	77.08	7.00	28.74	53.06
**(d)**	**(3,3,3)**							
Independent		9.48	9.82	10.00	85.70	9.30	33.10	57.22
EXNEX		7.08	8.10	7.32	79.94	8.36	24.00	59.56
$\text{EXNEX}_{\text{pool}}$		10.10	9.40	9.92	81.86	8.70	30.08	55.10
$\text{mEXNEX}_{\text{hist}}$		71.80	7.18	7.32	65.44	4.78	18.42	49.54
histFujikawa		8.68	8.52	9.50	64.52	5.68	23.16	44.82
EXppNEX		9.70	10.22	10.00	80.36	8.52	30.90	53.78
EXsamNEX		9.70	9.44	9.14	80.08	7.34	27.80	56.70
MLMixture		9.26	10.06	9.08	77.36	7.36	27.10	54.34

Table 11. *Simulation Results for scenario 8 under historic cases (a), (b), (c) and (d).*

	y_{k^*}	% Reject					FWER	% All Correct
**Scenario 8**		**0.25**	**0.1**	**0.1**	**0.25**	**0.1**		
**(a)**	**(1,1,1)**							
Independent		85.76	10.18	9.48	85.28	10.06	26.62	52.92
EXNEX		82.80	12.32	11.44	82.42	10.08	29.02	45.22
$\text{EXNEX}_{\text{pool}}$		83.06	12.02	11.96	81.24	9.76	27.74	45.14
$\text{mEXNEX}_{\text{hist}}$		78.66	14.28	13.94	76.96	8.94	27.50	41.56
histFujikawa		82.22	14.18	13.52	76.32	10.02	27.98	42.86
EXppNEX		86.72	11.72	10.60	82.74	10.96	29.54	49.42
EXsamNEX		86.16	11.72	11.04	82.22	10.66	30.06	47.40
MLMixture		86.10	11.92	12.26	82.12	10.02	28.72	48.42
**(b)**	**(3,1,1)**							
Independent		85.76	10.18	9.48	85.28	10.06	26.62	52.92
EXNEX		82.80	12.32	11.44	82.42	10.08	29.02	45.22
$\text{EXNEX}_{\text{pool}}$		88.20	12.22	11.72	81.10	10.36	29.22	48.08
$\text{mEXNEX}_{\text{hist}}$		86.68	13.04	13.78	80.68	10.10	28.14	47.42
histFujikawa		84.96	13.98	14.34	77.36	10.36	28.40	44.32
EXppNEX		88.50	11.76	11.54	83.24	11.30	30.74	49.72
EXsamNEX		86.74	11.96	11.80	81.30	10.46	30.03	47.92
MLMixture		87.16	12.26	11.72	80.06	9.12	27.48	48.56
**(c)**	**(3,3,1)**							
Independent		85.76	10.18	9.48	85.28	10.06	26.62	52.92
EXNEX		82.80	12.32	11.44	82.42	10.08	29.02	45.22
$\text{EXNEX}_{\text{pool}}$		86.94	13.04	12.04	81.82	11.10	30.18	47.26
$\text{mEXNEX}_{\text{hist}}$		82.98	15.24	12.44	81.48	9.66	30.12	44.22
histFujikawa		82.74	15.22	14.42	77.88	11.06	30.20	41.90
EXppNEX		86.18	12.40	11.76	83.52	10.60	30.22	48.52
EXsamNEX		86.24	12.62	11.40	81.40	10.54	29.44	47.64
MLMixture		85.90	13.22	11.64	80.80	9.88	28.34	47.40
**(d)**	**(3,3,3)**							
Independent		85.76	10.18	9.48	85.28	10.06	26.62	52.92
EXNEX		82.80	12.32	11.44	82.42	10.08	29.02	45.22
$\text{EXNEX}_{\text{pool}}$		84.66	12.12	12.42	83.94	10.74	29.94	47.96
$\text{mEXNEX}_{\text{hist}}$		79.12	14.30	14.16	77.58	9.22	27.90	41.82
histFujikawa		81.82	13.68	13.76	76.90	10.62	28.22	42.22
EXppNEX		86.14	11.88	12.26	82.06	9.72	28.96	48.24
EXsamNEX		83.62	12.72	13.08	81.82	10.22	29.56	45.66
MLMixture		84.74	12.48	12.62	80.44	9.94	28.22	45.82

Table 12. *Mean point estimate for the response rate (standard deviation) for scenario 1 under historic cases (a), (b), (c) and (d).*

	y_{k^*}	Mean Point Estimate (Sd)				
**Scenario 1**		**0.1**	**0.1**	**0.1**	**0.1**	**0.1**
**(a)**	**(1,1,1)**					
Independent		0.101 (0.051)	0.099 (0.050)	0.100 (0.051)	0.100 (0.051)	0.101 (0.051)
EXNEX		0.102 (0.039)	0.102 (0.041)	0.102 (0.040)	0.102 (0.039)	0.101 (0.040)
$\text{EXNEX}_{\text{pool}}$		0.096 (0.029)	0.096 (0.029)	0.095 (0.028)	0.101 (0.039)	0.101 (0.039)
$\text{mEXNEX}_{\text{hist}}$		0.100 (0.031)	0.100 (0.031)	0.100 (0.030)	0.101 (0.033)	0.101 (0.033)
histFujikawa		0.102 (0.029)	0.101 (0.030)	0.102 (0.030)	0.101 (0.029)	0.102 (0.030)
EXppNEX		0.107 (0.040)	0.108 (0.040)	0.107 (0.040)	0.101 (0.042)	0.101 (0.042)
EXsamNEX		0.104 (0.039)	0.104 (0.039)	0.105 (0.039)	0.105 (0.043)	0.105 (0.043)
MLMixture		0.103 (0.036)	0.104 (0.036)	0.104 (0.036)	0.103 (0.038)	0.103 (0.038)
**(b)**	**(3,1,1)**					
Independent		0.101 (0.051)	0.099 (0.050)	0.100 (0.051)	0.100 (0.051)	0.101 (0.051)
EXNEX		0.102 (0.039)	0.102 (0.041)	0.102 (0.040)	0.102 (0.039)	0.101 (0.040)
$\text{EXNEX}_{\text{pool}}$		0.129 (0.031)	0.099 (0.029)	0.099 (0.029)	0.103 (0.038)	0.102 (0.038)
$\text{mEXNEX}_{\text{hist}}$		0.102 (0.038)	0.101 (0.036)	0.101 (0.036)	0.102 (0.038)	0.102 (0.037)
histFujikawa		0.106 (0.033)	0.105 (0.032)	0.106 (0.032)	0.105 (0.032)	0.106 (0.033)
EXppNEX		0.113 (0.044)	0.107 (0.039)	0.107 (0.040)	0.100 (0.040)	0.102 (0.042)
EXsamNEX		0.107 (0.044)	0.104 (0.037)	0.104 (0.038)	0.104 (0.041)	0.104 (0.041)
MLMixture		0.113 (0.042)	0.105 (0.036)	0.105 (0.036)	0.105 (0.038)	0.105 (0.038)
**(c)**	**(3,3,1)**					
Independent		0.101 (0.051)	0.099 (0.050)	0.100 (0.051)	0.100 (0.051)	0.101 (0.051)
EXNEX		0.102 (0.039)	0.102 (0.041)	0.102 (0.040)	0.102 (0.039)	0.101 (0.040)
$\text{EXNEX}_{\text{pool}}$		0.130 (0.030)	0.130 (0.029)	0.101 (0.029)	0.106 (0.038)	0.105 (0.039)
$\text{mEXNEX}_{\text{hist}}$		0.101 (0.036)	0.100 (0.036)	0.101 (0.037)	0.101 (0.039)	0.101 (0.038)
histFujikawa		0.110 (0.036)	0.110 (0.036)	0.109 (0.037)	0.109 (0.036)	0.109 (0.036)
EXppNEX		0.113 (0.043)	0.113 (0.044)	0.107 (0.039)	0.100 (0.041)	0.101 (0.042)
EXsamNEX		0.107 (0.044)	0.107 (0.043)	0.104 (0.037)	0.103 (0.040)	0.104 (0.041)
MLMixture		0.114 (0.041)	0.114 (0.040)	0.107 (0.035)	0.107 (0.039)	0.107 (0.038)
**(d)**	**(3,3,3)**					
Independent		0.101 (0.051)	0.099 (0.050)	0.100 (0.051)	0.100 (0.051)	0.101 (0.051)
EXNEX		0.102 (0.039)	0.102 (0.041)	0.102 (0.040)	0.102 (0.039)	0.101 (0.040)
$\text{EXNEX}_{\text{pool}}$		0.134 (0.028)	0.133 (0.028)	0.133 (0.029)	0.109 (0.039)	0.109 (0.038)
$\text{mEXNEX}_{\text{hist}}$		0.100 (0.030)	0.100 (0.031)	0.100 (0.030)	0.100 (0.033)	0.100 (0.033)
histFujikawa		0.114 (0.040)	0.114 (0.039)	0.115 (0.040)	0.114 (0.040)	0.114 (0.039)
EXppNEX		0.113 (0.044)	0.113 (0.043)	0.112 (0.043)	0.099 (0.040)	0.100 (0.041)
EXsamNEX		0.106 (0.043)	0.107 (0.043)	0.107 (0.043)	0.104 (0.040)	0.105 (0.041)
MLMixture		0.115 (0.041)	0.116 (0.041)	0.115 (0.040)	0.109 (0.038)	0.109 (0.038)

Table 13. *Mean point estimate for the response rate (standard deviation) for scenario 2 under historic cases (a), (b), (c) and (d).*

	y_{k^*}	Mean Point Estimate (Sd)				
**Scenario 2**		**0.25**	**0.1**	**0.1**	**0.1**	**0.1**
**(a)**	**(1,1,1)**					
Independent		0.249 (0.075)	0.102 (0.051)	0.101 (0.052)	0.100 (0.051)	0.099 (0.051)
EXNEX		0.233 (0.074)	0.107 (0.043)	0.107 (0.043)	0.106 (0.043)	0.106 (0.043)
$\text{EXNEX}_{\text{pool}}$		0.187 (0.052)	0.100 (0.031)	0.100 (0.031)	0.111 (0.040)	0.112 (0.040)
$\text{mEXNEX}_{\text{hist}}$		0.202 (0.066)	0.112 (0.036)	0.114 (0.036)	0.112 (0.038)	0.111 (0.037)
histFujikawa		0.214 (0.083)	0.106 (0.034)	0.107 (0.035)	0.106 (0.034)	0.106 (0.034)
EXppNEX		0.229 (0.064)	0.110 (0.041)	0.110 (0.041)	0.104 (0.044)	0.104 (0.043)
EXsamNEX		0.239 (0.077)	0.108 (0.040)	0.107 (0.040)	0.110 (0.044)	0.110 (0.045)
MLMixture		0.224 (0.071)	0.106 (0.038)	0.106 (0.037)	0.106 (0.039)	0.106 (0.040)
**(b)**	**(3,1,1)**					
Independent		0.249 (0.075)	0.102 (0.051)	0.101 (0.052)	0.100 (0.051)	0.099 (0.051)
EXNEX		0.233 (0.074)	0.107 (0.043)	0.107 (0.043)	0.106 (0.043)	0.106 (0.043)
$\text{EXNEX}_{\text{pool}}$		0.229 (0.055)	0.100 (0.032)	0.100 (0.032)	0.106 (0.042)	0.105 (0.042)
$\text{mEXNEX}_{\text{hist}}$		0.230 (0.073)	0.106 (0.039)	0.106 (0.039)	0.106 (0.040)	0.105 (0.040)
histFujikawa		0.219 (0.076)	0.110 (0.037)	0.110 (0.037)	0.111 (0.037)	0.111 (0.037)
EXppNEX		0.246 (0.067)	0.110 (0.041)	0.109 (0.040)	0.104 (0.044)	0.104 (0.043)
EXsamNEX		0.243 (0.068)	0.107 (0.039)	0.106 (0.038)	0.109 (0.044)	0.108 (0.043)
MLMixture		0.239 (0.067)	0.108 (0.037)	0.107 (0.037)	0.109 (0.040)	0.110 (0.040)
**(c)**	**(3,3,1)**					
Independent		0.249 (0.075)	0.102 (0.051)	0.101 (0.052)	0.100 (0.051)	0.099 (0.051)
EXNEX		0.233 (0.074)	0.107 (0.043)	0.107 (0.043)	0.106 (0.043)	0.106 (0.043)
$\text{EXNEX}_{\text{pool}}$		0.228 (0.053)	0.135 (0.032)	0.103 (0.032)	0.108 (0.042)	0.110 (0.043)
$\text{mEXNEX}_{\text{hist}}$		0.220 (0.070)	0.111 (0.040)	0.109 (0.042)	0.109 (0.041)	0.108 (0.041)
histFujikawa		0.222 (0.068)	0.116 (0.040)	0.116 (0.039)	0.114 (0.039)	0.115 (0.040)
EXppNEX		0.246 (0.066)	0.118 (0.045)	0.109 (0.041)	0.104 (0.044)	0.103 (0.043)
EXsamNEX		0.244 (0.069)	0.109 (0.045)	0.108 (0.039)	0.107 (0.043)	0.107 (0.042)
MLMixture		0.241 (0.067)	0.117 (0.041)	0.109 (0.037)	0.111 (0.040)	0.112 (0.040)
**(d)**	**(3,3,3)**					
Independent		0.249 (0.075)	0.102 (0.051)	0.101 (0.052)	0.100 (0.051)	0.099 (0.051)
EXNEX		0.233 (0.074)	0.107 (0.043)	0.107 (0.043)	0.106 (0.043)	0.106 (0.043)
$\text{EXNEX}_{\text{pool}}$		0.226 (0.052)	0.139 (0.031)	0.139 (0.031)	0.112 (0.042)	0.112 (0.042)
$\text{mEXNEX}_{\text{hist}}$		0.202 (0.065)	0.114 (0.035)	0.113 (0.035)	0.111 (0.037)	0.111 (0.038)
histFujikawa		0.227 (0.063)	0.120 (0.043)	0.119 (0.044)	0.119 (0.043)	0.119 (0.042)
EXppNEX		0.245 (0.068)	0.117 (0.045)	0.116 (0.045)	0.104 (0.043)	0.102 (0.043)
EXsamNEX		0.242 (0.070)	0.110 (0.044)	0.111 (0.044)	0.107 (0.041)	0.108 (0.041)
MLMixture		0.239 (0.064)	0.120 (0.041)	0.121 (0.041)	0.114 (0.040)	0.115 (0.040)

Table 14. *Mean point estimate for the response rate (standard deviation) for scenario 3 under historic cases (a), (b), (c) and (d).*

	y_{k^*}	Mean Point Estimate (Sd)				
**Scenario 3**		**0.25**	**0.25**	**0.1**	**0.1**	**0.1**
**(a)**	**(1,1,1)**					
Independent		0.250 (0.075)	0.250 (0.073)	0.100 (0.051)	0.099 (0.049)	0.100 (0.051)
EXNEX		0.235 (0.069)	0.236 (0.069)	0.112 (0.046)	0.112 (0.046)	0.112 (0.046)
$\text{EXNEX}_{\text{pool}}$		0.190 (0.052)	0.191 (0.049)	0.105 (0.033)	0.111 (0.043)	0.112 (0.043)
$\text{mEXNEX}_{\text{hist}}$		0.218 (0.061)	0.218 (0.061)	0.126 (0.040)	0.120 (0.041)	0.120 (0.042)
histFujikawa		0.224 (0.075)	0.224 (0.075)	0.111 (0.039)	0.111 (0.038)	0.112 (0.039)
EXppNEX		0.232 (0.062)	0.230 (0.062)	0.114 (0.043)	0.108 (0.046)	0.109 (0.047)
EXsamNEX		0.239 (0.076)	0.238 (0.075)	0.113 (0.041)	0.116 (0.046)	0.116 (0.045)
MLMixture		0.228 (0.069)	0.228 (0.068)	0.110 (0.038)	0.113 (0.042)	0.112 (0.042)
**(b)**	**(3,1,1)**					
Independent		0.250 (0.075)	0.250 (0.073)	0.100 (0.051)	0.099 (0.049)	0.100 (0.051)
EXNEX		0.235 (0.069)	0.236 (0.069)	0.112 (0.046)	0.112 (0.046)	0.112 (0.046)
$\text{EXNEX}_{\text{pool}}$		0.230 (0.052)	0.193 (0.049)	0.105 (0.035)	0.112 (0.044)	0.112 (0.044)
$\text{mEXNEX}_{\text{hist}}$		0.230 (0.066)	0.226 (0.067)	0.118 (0.044)	0.114 (0.044)	0.114 (0.043)
histFujikawa		0.226 (0.067)	0.225 (0.068)	0.118 (0.041)	0.117 (0.041)	0.117 (0.040)
EXppNEX		0.247 (0.065)	0.230 (0.062)	0.113 (0.042)	0.108 (0.047)	0.108 (0.047)
EXsamNEX		0.245 (0.067)	0.240 (0.076)	0.110 (0.041)	0.114 (0.046)	0.114 (0.045)
MLMixture		0.239 (0.065)	0.228 (0.068)	0.112 (0.039)	0.114 (0.041)	0.116 (0.042)
**(c)**	**(3,3,1)**					
Independent		0.250 (0.075)	0.250 (0.073)	0.100 (0.051)	0.099 (0.049)	0.100 (0.051)
EXNEX		0.235 (0.069)	0.236 (0.069)	0.112 (0.046)	0.112 (0.046)	0.112 (0.046)
$\text{EXNEX}_{\text{pool}}$		0.232 (0.052)	0.233 (0.051)	0.105 (0.036)	0.113 (0.046)	0.113 (0.045)
$\text{mEXNEX}_{\text{hist}}$		0.228 (0.065)	0.228 (0.065)	0.116 (0.062)	0.116 (0.045)	0.117 (0.045)
histFujikawa		0.231 (0.064)	0.231 (0.064)	0.120 (0.044)	0.121 (0.044)	0.120 (0.044)
EXppNEX		0.248 (0.065)	0.248 (0.065)	0.112 (0.042)	0.107 (0.046)	0.107 (0.045)
EXsamNEX		0.244 (0.067)	0.246 (0.068)	0.109 (0.041)	0.112 (0.045)	0.113 (0.045)
MLMixture		0.240 (0.063)	0.241 (0.064)	0.115 (0.039)	0.117 (0.042)	0.117 (0.042)
**(d)**	**(3,3,3)**					
Independent		0.250 (0.075)	0.250 (0.073)	0.100 (0.051)	0.099 (0.049)	0.100 (0.051)
EXNEX		0.235 (0.069)	0.236 (0.069)	0.112 (0.046)	0.112 (0.046)	0.112 (0.046)
$\text{EXNEX}_{\text{pool}}$		0.231 (0.051)	0.230 (0.050)	0.144 (0.032)	0.116 (0.046)	0.115 (0.045)
$\text{mEXNEX}_{\text{hist}}$		0.217 (0.061)	0.219 (0.061)	0.126 (0.039)	0.121 (0.042)	0.121 (0.041)
histFujikawa		0.235 (0.059)	0.234 (0.058)	0.126 (0.048)	0.126 (0.048)	0.126 (0.048)
EXppNEX		0.246 (0.065)	0.247 (0.065)	0.120 (0.045)	0.107 (0.045)	0.106 (0.045)
EXsamNEX		0.243 (0.068)	0.243 (0.068)	0.115 (0.047)	0.111 (0.043)	0.113 (0.044)
MLMixture		0.241 (0.064)	0.241 (0.063)	0.125 (0.043)	0.121 (0.042)	0.119 (0.043)

Table 15. *Mean point estimate for the response rate (standard deviation) for scenario 4 under historic cases (a), (b), (c) and (d).*

	y_{k^*}	Mean Point Estimate (Sd)				
**Scenario 4**		**0.25**	**0.25**	**0.25**	**0.1**	**0.1**
**(a)**	**(1,1,1)**					
Independent		0.250 (0.075)	0.249 (0.075)	0.250 (0.074)	0.099 (0.051)	0.100 (0.052)
EXNEX		0.241 (0.067)	0.240 (0.066)	0.240 (0.065)	0.118 (0.047)	0.117 (0.049)
$\text{EXNEX}_{\text{pool}}$		0.194 (0.046)	0.195 (0.046)	0.195 (0.046)	0.118 (0.046)	0.117 (0.046)
$\text{mEXNEX}_{\text{hist}}$		0.230 (0.056)	0.229 (0.055)	0.230 (0.056)	0.131 (0.041)	0.131 (0.046)
histFujikawa		0.231 (0.068)	0.232 (0.068)	0.233 (0.068)	0.119 (0.043)	0.118 (0.042)
EXppNEX		0.234 (0.061)	0.233 (0.060)	0.233 (0.061)	0.112 (0.048)	0.113 (0.048)
EXsamNEX		0.240 (0.071)	0.241 (0.071)	0.240 (0.071)	0.121 (0.047)	0.121 (0.047)
MLMixture		0.229 (0.065)	0.229 (0.065)	0.230 (0.066)	0.119 (0.043)	0.119 (0.044)
**(b)**	**(3,1,1)**					
Independent		0.250 (0.075)	0.249 (0.075)	0.250 (0.074)	0.099 (0.051)	0.100 (0.052)
EXNEX		0.241 (0.067)	0.240 (0.066)	0.240 (0.065)	0.118 (0.047)	0.117 (0.049)
$\text{EXNEX}_{\text{pool}}$		0.230 (0.048)	0.198 (0.046)	0.198 (0.045)	0.119 (0.047)	0.119 (0.047)
$\text{mEXNEX}_{\text{hist}}$		0.240 (0.063)	0.235 (0.061)	0.235 (0.062)	0.122 (0.049)	0.123 (0.049)
histFujikawa		0.233 (0.063)	0.234 (0.062)	0.234 (0.063)	0.123 (0.046)	0.123 (0.045)
EXppNEX		0.250 (0.063)	0.233 (0.060)	0.234 (0.060)	0.113 (0.049)	0.113 (0.049)
EXsamNEX		0.245 (0.064)	0.240 (0.073)	0.240 (0.072)	0.121 (0.048)	0.121 (0.048)
MLMixture		0.243 (0.062)	0.230 (0.065)	0.230 (0.065)	0.122 (0.044)	0.122 (0.044)
**(c)**	**(3,3,1)**					
Independent		0.250 (0.075)	0.249 (0.075)	0.250 (0.074)	0.099 (0.051)	0.100 (0.052)
EXNEX		0.241 (0.067)	0.240 (0.066)	0.240 (0.065)	0.118 (0.047)	0.117 (0.049)
$\text{EXNEX}_{\text{pool}}$		0.235 (0.048)	0.233 (0.048)	0.199 (0.046)	0.118 (0.048)	0.118 (0.048)
$\text{mEXNEX}_{\text{hist}}$		0.237 (0.061)	0.235 (0.061)	0.239 (0.062)	0.122 (0.048)	0.123 (0.049)
histFujikawa		0.235 (0.058)	0.235 (0.057)	0.236 (0.057)	0.130 (0.048)	0.129 (0.048)
EXppNEX		0.248 (0.063)	0.248 (0.063)	0.233 (0.061)	0.111 (0.048)	0.111 (0.049)
EXsamNEX		0.246 (0.065)	0.245 (0.065)	0.242 (0.073)	0.120 (0.047)	0.120 (0.046)
MLMixture		0.242 (0.060)	0.244 (0.062)	0.232 (0.065)	0.126 (0.044)	0.124 (0.043)
**(d)**	**(3,3,3)**					
Independent		0.250 (0.075)	0.249 (0.075)	0.250 (0.074)	0.099 (0.051)	0.100 (0.052)
EXNEX		0.241 (0.067)	0.240 (0.066)	0.240 (0.065)	0.118 (0.047)	0.117 (0.049)
$\text{EXNEX}_{\text{pool}}$		0.237 (0.047)	0.236 (0.046)	0.235 (0.046)	0.119 (0.049)	0.119 (0.050)
$\text{mEXNEX}_{\text{hist}}$		0.229 (0.057)	0.230 (0.056)	0.228 (0.056)	0.132 (0.046)	0.139 (0.046)
histFujikawa		0.239 (0.053)	0.239 (0.052)	0.239 (0.053)	0.135 (0.052)	0.135 (0.052)
EXppNEX		0.250 (0.060)	0.250 (0.063)	0.247 (0.062)	0.111 (0.047)	0.111 (0.048)
EXsamNEX		0.247 (0.065)	0.245 (0.065)	0.245 (0.064)	0.118 (0.047)	0.118 (0.048)
MLMixture		0.244 (0.061)	0.243 (0.061)	0.243 (0.060)	0.127 (0.045)	0.128 (0.045)

Table 16. *Mean point estimate for the response rate (standard deviation) for scenario 5 under historic cases (a), (b), (c) and (d).*

	y_{k^*}	Mean Point Estimate (Sd)				
**Scenario 5**		**0.25**	**0.25**	**0.25**	**0.25**	**0.1**
**(a)**	**(1,1,1)**					
Independent		0.249 (0.074)	0.250 (0.074)	0.251 (0.075)	0.248 (0.073)	0.101 (0.051)
EXNEX		0.245 (0.062)	0.246 (0.062)	0.246 (0.062)	0.245 (0.062)	0.112 (0.053)
$\text{EXNEX}_{\text{pool}}$		0.201 (0.044)	0.201 (0.043)	0.201 (0.044)	0.235 (0.061)	0.123 (0.048)
$\text{mEXNEX}_{\text{hist}}$		0.241 (0.052)	0.239 (0.051)	0.240 (0.051)	0.241 (0.053)	0.140 (0.052)
histFujikawa		0.237 (0.063)	0.237 (0.064)	0.238 (0.064)	0.235 (0.063)	0.126 (0.046)
EXppNEX		0.240 (0.059)	0.238 (0.058)	0.239 (0.058)	0.246 (0.064)	0.119 (0.052)
EXsamNEX		0.244 (0.068)	0.244 (0.067)	0.244 (0.068)	0.250 (0.065)	0.131 (0.050)
MLMixture		0.235 (0.064)	0.235 (0.064)	0.234 (0.063)	0.245 (0.067)	0.128 (0.046)
**(b)**	**(3,1,1)**					
Independent		0.249 (0.074)	0.250 (0.074)	0.251 (0.075)	0.248 (0.073)	0.101 (0.051)
EXNEX		0.245 (0.062)	0.246 (0.062)	0.246 (0.062)	0.245 (0.062)	0.112 (0.053)
$\text{EXNEX}_{\text{pool}}$		0.235 (0.045)	0.204 (0.044)	0.204 (0.043)	0.240 (0.061)	0.123 (0.050)
$\text{mEXNEX}_{\text{hist}}$		0.245 (0.059)	0.242 (0.056)	0.244 (0.056)	0.244 (0.058)	0.126 (0.052)
histFujikawa		0.237 (0.058)	0.239 (0.058)	0.239 (0.058)	0.238 (0.058)	0.131 (0.049)
EXppNEX		0.253 (0.058)	0.239 (0.059)	0.239 (0.049)	0.247 (0.065)	0.119 (0.053)
EXsamNEX		0.253 (0.057)	0.250 (0.064)	0.248 (0.065)	0.255 (0.059)	0.256 (0.059)
MLMixture		0.244 (0.059)	0.234 (0.063)	0.234 (0.062)	0.246 (0.066)	0.129 (0.047)
**(c)**	**(3,3,1)**					
Independent		0.249 (0.074)	0.250 (0.074)	0.251 (0.075)	0.248 (0.073)	0.101 (0.051)
EXNEX		0.245 (0.062)	0.246 (0.062)	0.246 (0.062)	0.245 (0.062)	0.112 (0.053)
$\text{EXNEX}_{\text{pool}}$		0.238 (0.045)	0.238 (0.044)	0.206 (0.044)	0.242 (0.060)	0.125 (0.052)
$\text{mEXNEX}_{\text{hist}}$		0.242 (0.056)	0.242 (0.057)	0.242 (0.059)	0.245 (0.058)	0.126 (0.052)
histFujikawa		0.241 (0.054)	0.242 (0.053)	0.241 (0.054)	0.242 (0.053)	0.137 (0.052)
EXppNEX		0.251 (0.060)	0.251 (0.060)	0.238 (0.058)	0.243 (0.066)	0.119 (0.051)
EXsamNEX		0.249 (0.062)	0.250 (0.061)	0.245 (0.070)	0.249 (0.065)	0.129 (0.049)
MLMixture		0.246 (0.057)	0.246 (0.057)	0.235 (0.060)	0.247 (0.064)	0.135 (0.047)
**(d)**	**(3,3,3)**					
Independent		0.249 (0.074)	0.250 (0.074)	0.251 (0.075)	0.248 (0.073)	0.101 (0.051)
EXNEX		0.245 (0.062)	0.246 (0.062)	0.246 (0.062)	0.245 (0.062)	0.112 (0.053)
$\text{EXNEX}_{\text{pool}}$		0.241 (0.044)	0.241 (0.043)	0.241 (0.044)	0.244 (0.058)	0.124 (0.053)
$\text{mEXNEX}_{\text{hist}}$		0.241 (0.052)	0.240 (0.051)	0.240 (0.051)	0.242 (0.054)	0.139 (0.052)
histFujikawa		0.244 (0.050)	0.245 (0.049)	0.244 (0.050)	0.244 (0.049)	0.145 (0.055)
EXppNEX		0.252 (0.060)	0.252 (0.059)	0.253 (0.060)	0.243 (0.067)	0.116 (0.051)
EXsamNEX		0.250 (0.060)	0.249 (0.063)	0.249 (0.061)	0.250 (0.068)	0.128 (0.050)
MLMixture		0.248 (0.056)	0.246 (0.057)	0.246 (0.056)	0.248 (0.062)	0.135 (0.049)

Table 17. *Mean point estimate for the response rate (standard deviation) for scenario 6 under historic cases (a), (b), (c) and (d).*

	y_{k^*}	Mean Point Estimate (Sd)				
**Scenario 6**		**0.25**	**0.25**	**0.25**	**0.25**	**0.25**
**(a)**	**(1,1,1)**					
Independent		0.249 (0.074)	0.250 (0.075)	0.249 (0.074)	0.250 (0.075)	0.248 (0.075)
EXNEX		0.251 (0.058)	0.250 (0.058)	0.250 (0.057)	0.251 (0.058)	0.250 (0.058)
$\text{EXNEX}_{\text{pool}}$		0.208 (0.042)	0.207 (0.042)	0.206 (0.043)	0.241 (0.058)	0.240 (0.057)
$\text{mEXNEX}_{\text{hist}}$		0.250 (0.046)	0.250 (0.045)	0.251 (0.046)	0.250 (0.049)	0.249 (0.048)
histFujikawa		0.241 (0.057)	0.242 (0.058)	0.242 (0.057)	0.242 (0.057)	0.243 (0.057)
EXppNEX		0.242 (0.057)	0.243 (0.056)	0.243 (0.056)	0.252 (0.062)	0.251 (0.060)
EXsamNEX		0.250 (0.064)	0.247 (0.065)	0.247 (0.064)	0.256 (0.060)	0.256 (0.060)
MLMixture		0.238 (0.060)	0.239 (0.06)	0.238 (0.060)	0.248 (0.060)	0.250 (0.062)
**(b)**	**(3,1,1)**					
Independent		0.249 (0.074)	0.250 (0.075)	0.249 (0.074)	0.250 (0.075)	0.248 (0.075)
EXNEX		0.251 (0.058)	0.250 (0.058)	0.250 (0.057)	0.251 (0.058)	0.250 (0.058)
$\text{EXNEX}_{\text{pool}}$		0.239 (0.042)	0.209 (0.043)	0.210 (0.043)	0.243 (0.057)	0.242 (0.057)
$\text{mEXNEX}_{\text{hist}}$		0.250 (0.055)	0.248 (0.051)	0.250 (0.051)	0.250 (0.054)	0.249 (0.054)
histFujikawa		0.244 (0.053)	0.245 (0.052)	0.245 (0.053)	0.243 (0.053)	0.245 (0.053)
EXppNEX		0.255 (0.055)	0.243 (0.055)	0.243 (0.056)	0.250 (0.061)	0.104 (0.061)
EXsamNEX		0.114 (0.047)	0.108 (0.040)	0.108 (0.039)	0.224 (0.076)	0.110 (0.044)
MLMixture		0.250 (0.055)	0.239 (0.057)	0.239 (0.057)	0.248 (0.058)	0.250 (0.059)
**(c)**	**(3,3,1)**					
Independent		0.249 (0.074)	0.250 (0.075)	0.249 (0.074)	0.250 (0.075)	0.248 (0.075)
EXNEX		0.251 (0.058)	0.250 (0.058)	0.250 (0.057)	0.251 (0.058)	0.250 (0.058)
$\text{EXNEX}_{\text{pool}}$		0.243 (0.042)	0.242 (0.042)	0.213 (0.043)	0.246 (0.054)	0.246 (0.056)
$\text{mEXNEX}_{\text{hist}}$		0.251 (0.052)	0.250 (0.053)	0.250 (0.055)	0.250 (0.055)	0.251 (0.056)
histFujikawa		0.248 (0.048)	0.247 (0.048)	0.248 (0.050)	0.248 (0.049)	0.248 (0.049)
EXppNEX		0.255 (0.056)	0.256 (0.057)	0.243 (0.056)	0.250 (0.062)	0.250 (0.061)
EXsamNEX		0.254 (0.057)	0.254 (0.057)	0.250 (0.067)	0.255 (0.060)	0.255 (0.061)
MLMixture		0.251 (0.054)	0.252 (0.054)	0.241 (0.057)	0.252 (0.058)	0.251 (0.059)
**(d)**	**(3,3,3)**					
Independent		0.249 (0.074)	0.250 (0.075)	0.249 (0.074)	0.250 (0.075)	0.248 (0.075)
EXNEX		0.251 (0.058)	0.250 (0.058)	0.250 (0.057)	0.251 (0.058)	0.250 (0.058)
$\text{EXNEX}_{\text{pool}}$		0.244 (0.041)	0.245 (0.041)	0.245 (0.040)	0.247 (0.056)	0.248 (0.055)
$\text{mEXNEX}_{\text{hist}}$		0.248 (0.045)	0.249 (0.046)	0.249 (0.046)	0.248 (0.048)	0.249 (0.048)
histFujikawa		0.250 (0.045)	0.251 (0.045)	0.251 (0.045)	0.250 (0.044)	0.250 (0.045)
EXppNEX		0.254 (0.056)	0.254 (0.057)	0.255 (0.056)	0.249 (0.062)	0.248 (0.061)
EXsamNEX		0.253 (0.058)	0.253 (0.057)	0.252 (0.058)	0.255 (0.062)	0.254 (0.061)
MLMixture		0.252 (0.053)	0.253 (0.052)	0.252 (0.053)	0.254 (0.055)	0.254 (0.057)

Table 18. *Mean point estimate for the response rate (standard deviation) for scenario 7 under historic cases (a), (b), (c) and (d).*

	y_{k^*}	Mean Point Estimate (Sd)				
**Scenario 7**		**0.1**	**0.1**	**0.1**	**0.25**	**0.1**
**(a)**	**(1,1,1)**					
Independent		0.100 (0.051)	0.101 (0.051)	0.100 (0.052)	0.249 (0.075)	0.101 (0.052)
EXNEX		0.105 (0.042)	0.106 (0.043)	0.106 (0.042)	0.233 (0.073)	0.107 (0.043)
$\text{EXNEX}_{\text{pool}}$		0.098 (0.030)	0.099 (0.030)	0.098 (0.031)	0.232 (0.074)	0.104 (0.040)
$\text{mEXNEX}_{\text{hist}}$		0.109 (0.035)	0.109 (0.034)	0.110 (0.035)	0.213 (0.072)	0.108 (0.037)
histFujikawa		0.107 (0.034)	0.106 (0.034)	0.107 (0.034)	0.216 (0.084)	0.106 (0.034)
EXppNEX		0.111 (0.041)	0.112 (0.041)	0.112 (0.041)	0.235 (0.073)	0.105 (0.044)
EXsamNEX		0.108 (0.041)	0.109 (0.040)	0.107 (0.040)	0.242 (0.076)	0.110 (0.044)
MLMixture		0.107 (0.036)	0.106 (0.037)	0.107 (0.036)	0.240 (0.077)	0.107 (0.040)
**(b)**	**(3,1,1)**					
Independent		0.100 (0.051)	0.101 (0.051)	0.100 (0.052)	0.249 (0.075)	0.101 (0.052)
EXNEX		0.105 (0.042)	0.106 (0.043)	0.106 (0.042)	0.233 (0.073)	0.107 (0.043)
$\text{EXNEX}_{\text{pool}}$		0.134 (0.032)	0.102 (0.031)	0.101 (0.031)	0.231 (0.073)	0.108 (0.041)
$\text{mEXNEX}_{\text{hist}}$		0.107 (0.040)	0.107 (0.039)	0.107 (0.039)	0.228 (0.074)	0.107 (0.041)
histFujikawa		0.112 (0.037)	0.111 (0.037)	0.112 (0.037)	0.219 (0.075)	0.111 (0.038)
EXppNEX		0.119 (0.045)	0.111 (0.042)	0.110 (0.041)	0.234 (0.074)	0.104 (0.043)
EXsamNEX		0.245 (0.066)	0.110 (0.042)	0.111 (0.040)	0.246 (0.073)	0.116 (0.047)
MLMixture		0.116 (0.041)	0.109 (0.037)	0.108 (0.037)	0.240 (0.078)	0.111 (0.040)
**(c)**	**(3,3,1)**					
Independent		0.100 (0.051)	0.101 (0.051)	0.100 (0.052)	0.249 (0.075)	0.101 (0.052)
EXNEX		0.105 (0.042)	0.106 (0.043)	0.106 (0.042)	0.233 (0.073)	0.107 (0.043)
$\text{EXNEX}_{\text{pool}}$		0.135 (0.030)	0.136 (0.031)	0.103 (0.031)	0.230 (0.072)	0.109 (0.041)
$\text{mEXNEX}_{\text{hist}}$		0.106 (0.039)	0.107 (0.039)	0.106 (0.041)	0.230 (0.074)	0.107 (0.041)
histFujikawa		0.116 (0.040)	0.116 (0.040)	0.116 (0.041)	0.221 (0.068)	0.115 (0.040)
EXppNEX		0.117 (0.045)	0.117 (0.045)	0.110 (0.040)	0.233 (0.074)	0.105 (0.044)
EXsamNEX		0.111 (0.045)	0.112 (0.045)	0.108 (0.039)	0.242 (0.076)	0.109 (0.042)
MLMixture		0.118 (0.042)	0.118 (0.041)	0.110 (0.038)	0.238 (0.075)	0.112 (0.040)
**(d)**	**(3,3,3)**					
Independent		0.100 (0.051)	0.101 (0.051)	0.100 (0.052)	0.249 (0.075)	0.101 (0.052)
EXNEX		0.105 (0.042)	0.106 (0.043)	0.106 (0.042)	0.233 (0.073)	0.107 (0.043)
$\text{EXNEX}_{\text{pool}}$		0.139 (0.030)	0.139 (0.029)	0.139 (0.030)	0.229 (0.070)	0.114 (0.040)
$\text{mEXNEX}_{\text{hist}}$		0.109 (0.035)	0.109 (0.035)	0.109 (0.035)	0.214 (0.072)	0.108 (0.037)
histFujikawa		0.119 (0.044)	0.118 (0.043)	0.120 (0.043)	0.225 (0.061)	0.119 (0.043)
EXppNEX		0.116 (0.044)	0.117 (0.045)	0.118 (0.044)	0.234 (0.075)	0.104 (0.042)
EXsamNEX		0.111 (0.045)	0.111 (0.044)	0.111 (0.044)	0.243 (0.077)	0.108 (0.042)
MLMixture		0.121 (0.042)	0.121 (0.042)	0.120 (0.042)	0.238 (0.075)	0.114 (0.041)

Table 19. *Mean point estimate for the response rate (standard deviation) for scenario 8 under historic cases (a), (b), (c) and (d).*

	y_{k^*}	Mean Point Estimate (Sd)				
**Scenario 8**		**0.25**	**0.1**	**0.1**	**0.25**	**0.1**
**(a)**	**(1,1,1)**					
Independent		0.250 (0.073)	0.100 (0.051)	0.100 (0.052)	0.250 (0.074)	0.101 (0.051)
EXNEX		0.237 (0.070)	0.113 (0.046)	0.112 (0.046)	0.235 (0.069)	0.112 (0.045)
$\text{EXNEX}_{\text{pool}}$		0.191 (0.051)	0.103 (0.033)	0.103 (0.033)	0.232 (0.070)	0.110 (0.043)
$\text{mEXNEX}_{\text{hist}}$		0.214 (0.062)	0.123 (0.039)	0.122 (0.039)	0.222 (0.066)	0.118 (0.040)
histFujikawa		0.226 (0.076)	0.111 (0.038)	0.112 (0.034)	0.226 (0.075)	0.112 (0.038)
EXppNEX		0.233 (0.061)	0.114 (0.043)	0.114 (0.042)	0.238 (0.072)	0.109 (0.046)
EXsamNEX		0.241 (0.076)	0.112 (0.042)	0.110 (0.040)	0.247 (0.072)	0.117 (0.047)
MLMixture		0.227 (0.068)	0.111 (0.038)	0.110 (0.039)	0.241 (0.074)	0.113 (0.042)
**(b)**	**(3,1,1)**					
Independent		0.250 (0.073)	0.100 (0.051)	0.100 (0.052)	0.250 (0.074)	0.101 (0.051)
EXNEX		0.237 (0.070)	0.113 (0.046)	0.112 (0.046)	0.235 (0.069)	0.112 (0.045)
$\text{EXNEX}_{\text{pool}}$		0.233 (0.052)	0.104 (0.034)	0.104 (0.034)	0.234 (0.070)	0.111 (0.044)
$\text{mEXNEX}_{\text{hist}}$		0.232 (0.070)	0.115 (0.043)	0.115 (0.043)	0.230 (0.069)	0.112 (0.044)
histFujikawa		0.226 (0.068)	0.116 (0.041)	0.117 (0.041)	0.229 (0.069)	0.116 (0.041)
EXppNEX		0.249 (0.065)	0.114 (0.043)	0.114 (0.042)	0.239 (0.071)	0.110 (0.047)
EXsamNEX		0.244 (0.067)	0.116 (0.047)	0.110 (0.040)	0.245 (0.074)	0.115 (0.046)
MLMixture		0.241 (0.065)	0.112 (0.039)	0.112 (0.039)	0.240 (0.075)	0.115 (0.041)
**(c)**	**(3,3,1)**					
Independent		0.250 (0.073)	0.100 (0.051)	0.100 (0.052)	0.250 (0.074)	0.101 (0.051)
EXNEX		0.237 (0.070)	0.113 (0.046)	0.112 (0.046)	0.235 (0.069)	0.112 (0.045)
$\text{EXNEX}_{\text{pool}}$		0.232 (0.051)	0.142 (0.033)	0.107 (0.035)	0.232 (0.068)	0.114 (0.045)
$\text{mEXNEX}_{\text{hist}}$		0.226 (0.068)	0.118 (0.044)	0.114 (0.044)	0.232 (0.069)	0.113 (0.044)
histFujikawa		0.230 (0.063)	0.123 (0.044)	0.122 (0.044)	0.230 (0.063)	0.123 (0.044)
EXppNEX		0.248 (0.065)	0.124 (0.046)	0.114 (0.042)	0.237 (0.071)	0.108 (0.046)
EXsamNEX		0.244 (0.045)	0.116 (0.047)	0.110 (0.040)	0.245 (0.074)	0.115 (0.046)
MLMixture		0.241 (0.063)	0.124 (0.043)	0.114 (0.039)	0.239 (0.072)	0.117 (0.042)
**(d)**	**(3,3,3)**					
Independent		0.250 (0.073)	0.100 (0.051)	0.100 (0.052)	0.250 (0.074)	0.101 (0.051)
EXNEX		0.237 (0.070)	0.113 (0.046)	0.112 (0.046)	0.235 (0.069)	0.112 (0.045)
$\text{EXNEX}_{\text{pool}}$		0.231 (0.051)	0.145 (0.032)	0.145 (0.032)	0.233 (0.067)	0.117 (0.045)
$\text{mEXNEX}_{\text{hist}}$		0.214 (0.062)	0.123 (0.039)	0.123 (0.039)	0.223 (0.065)	0.118 (0.040)
histFujikawa		0.233 (0.057)	0.126 (0.048)	0.127 (0.047)	0.233 (0.057)	0.126 (0.048)
EXppNEX		0.246 (0.064)	0.123 (0.046)	0.123 (0.045)	0.235 (0.072)	0.106 (0.044)
EXsamNEX		0.243 (0.068)	0.116 (0.046)	0.116 (0.047)	0.243 (0.074)	0.114 (0.044)
MLMixture		0.241 (0.063)	0.126 (0.043)	0.126 (0.043)	0.238 (0.070)	0.121 (0.043)

G. Computational Time of Proposed Approaches

Each of the seven approaches explored in the simulation study vary in their model complexity and thus have varying computational intensity. For example, the $\text{Fujikawa}_{\text{hist}}$ approach has an analytical form, therefore does not require MCMC methods, making the model fit far quicker than all other approaches. In contrast the MLMixture model requires the mixture of two EXNEX models under which each of the K baskets are modelled separately, resulting in slow computation time. To add to this, the computation time will only increase as the total number of baskets on the trial increases (as demonstrated in Figures 3 and 4). The MLMixture models' computation time is further increased with the number of historic baskets also present.

Table 20. *Computation time in seconds of all seven approaches measured in seconds. Each model is fit 100 times to the same data and the average computational time is taken and presented alongside the standard deviation. This is done for five different data sets (historic data available for the first three).*

**Method**	$y_k = (3,3,3,3,3)$ $y_{k^*} = (1,1,1,0,0)$	$y_k = (9,9,3,3,3)$ $y_{k^*} = (3,3,1,0,0)$	$y_k = (9,9,3,3,3)$ $y_{k^*} = (1,1,3,0,0)$	$y_k = (9,9,9,9,9)$ $y_{k^*} = (3,3,3,0,0)$	$y_k = (9,9,9,9,9)$ $y_{k^*} = (3,1,1,0,0)$
EXNEX	11.556 (0.147)	11.279 (0.129)	11.012 (0.111)	11.024 (0.138)	11.105 (0.138)
$\text{EXNEX}_{\text{pool}}$	11.083 (0.132)	11.145 (0.101)	10.861 (0.107)	11.011 (0.126)	11.067 (0.155)
$\text{mEXNEX}_{\text{hist}}$	11.025 (0.128)	11.023 (0.110)	10.764 (0.101)	10.819 (0.103)	10.910 (0.140)
histFujikawa	0.538 (0.013)	0.468 (0.014)	0.481 (0.015)	0.444 (0.015)	0.471 (0.014)
EXppNEX	12.052 (0.126)	11.869 (0.151)	11.602 (0.137)	11.725 (0.160)	11.500 (0.129)
EXsamNEX	16.115 (0.160)	15.910 (0.147)	15.915 (0.111)	16.100 (0.157)	15.778 (0.110)
MLMixture	166.530 (1.380)	165.368 (0.641)	163.718 (1.315)	167.203 (1.793)	161.857 (0.684)

Table 20 presents the average computation time for each approach for a several fixed data sets. Each average is computed across 100 simulation runs for the same data with the standard deviation also presented. This is considered for five separate data sets, considering different combinations of effective/ineffective baskets and homogeneity/heterogeneity levels. From the results in Table 20, it is clear that the data scenario has little effect on the computation time and thus it is expected that the only impacting factor will be the number of baskets present.

As expected, the histFujikawa approach takes a significantly shorter amount of time to conduct the model fit, averaging around half a second for each data set with a small standard deviation.

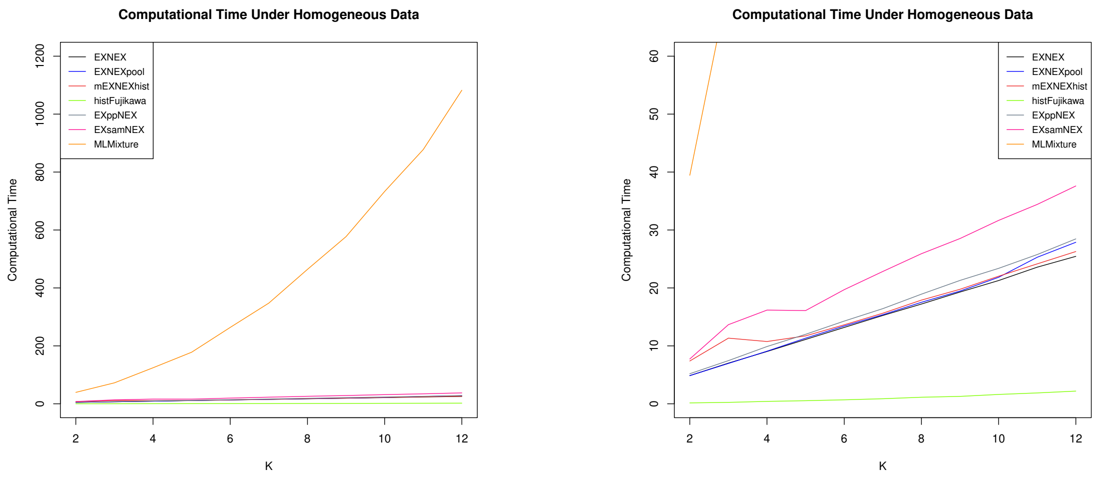

Fig. 3: Average computational time for a model fit on a fixed data set as the number of baskets, K changes. The Figure on the right is a zoomed-in version of the Figure on the left in order to distinguish the differences between methods. The fixed data set has all baskets homogeneous with current baskets each having a sample size of 34 with a total of 3 responses observed. Historic baskets have a sample size of 13 with 1 response observed. The number of historic baskets is $\lfloor K/2 \rfloor$.

At the opposite end of the spectrum, the MLMixture model takes around 15 times longer to fit the model compared to the standard EXNEX model. The EXNEX, $\text{EXNEX}_{\text{pool}}$, $\text{mEXNEX}_{\text{hist}}$ and EXppNEX approaches all take a similar amount of time, ranging from 10.8-12.1 seconds. The EXsamNEX model takes a couple of seconds longer at around 15.9 seconds due to the computation of mixture weights.

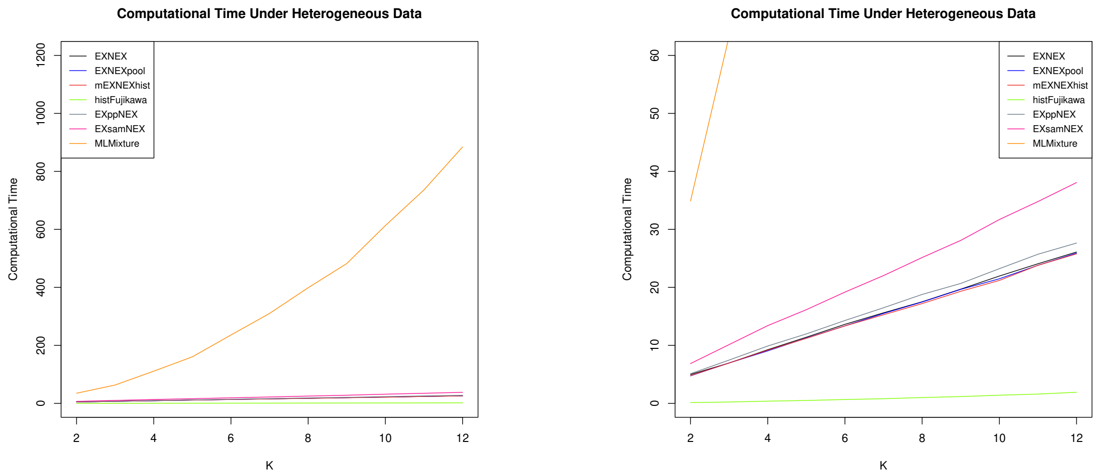

Fig. 4: Average computational time for a model fit on a fixed data set as the number of baskets, K changes. The Figure on the right is a zoomed-in version of the Figure on the left in order to distinguish the differences between methods. The fixed data set has heterogeneity with even numbered baskets observing 9 responses and odd 3 responses. Historic baskets observe 1 response. Current baskets have a sample size of 34 and historic baskets have a sample size of 13. The number of historic baskets is $\lfloor K/2 \rfloor$.

H. Sensitivity Analysis of α in the EXppNEX Approach

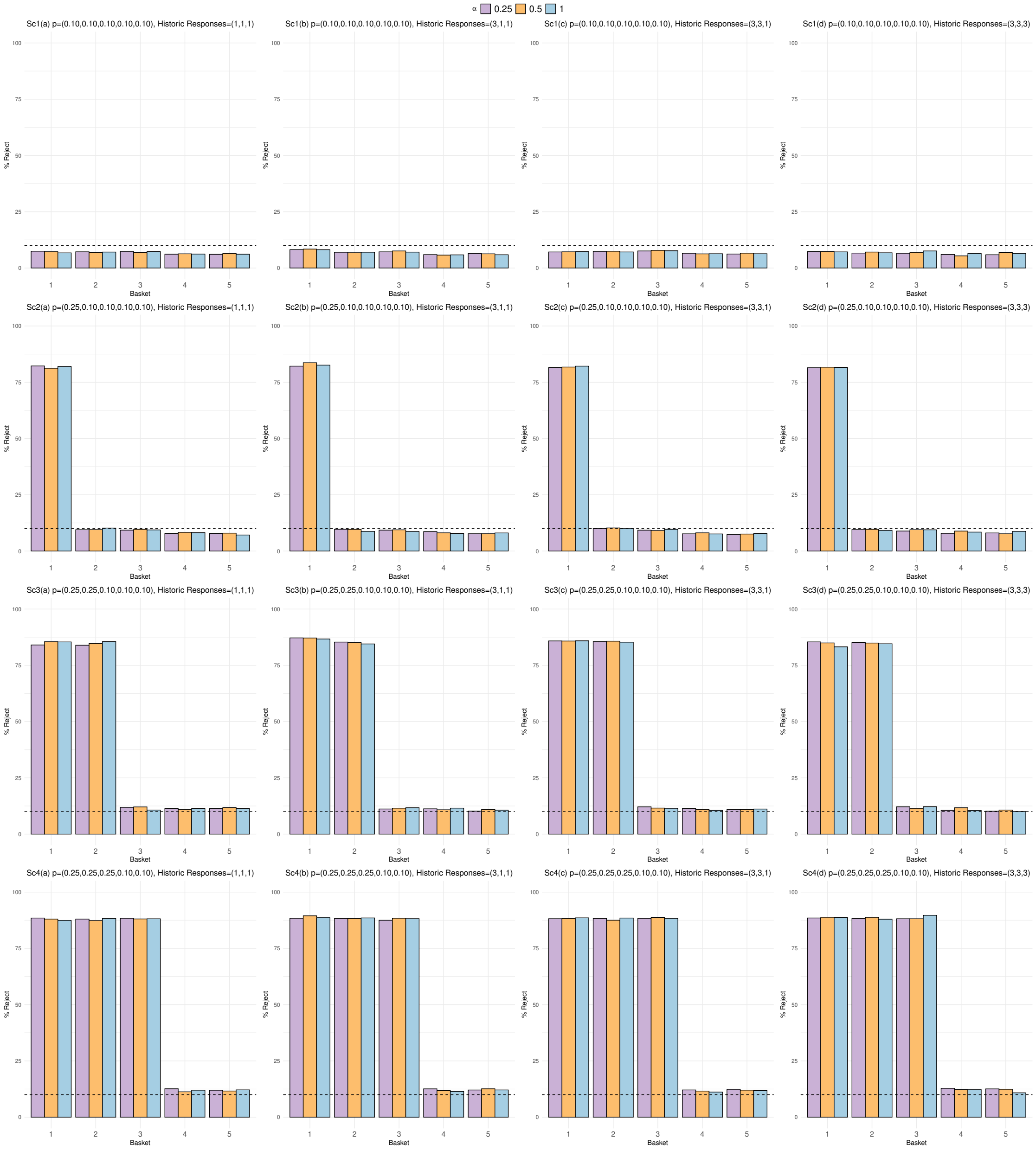

Fig. 5: The percentage of data sets where the null hypothesis were rejected per baskets under the EXppNEX model for scenarios 1-4 and 4 historic sub-cases. This is provided for three choices of α: 0.25, 0.5 and 1.

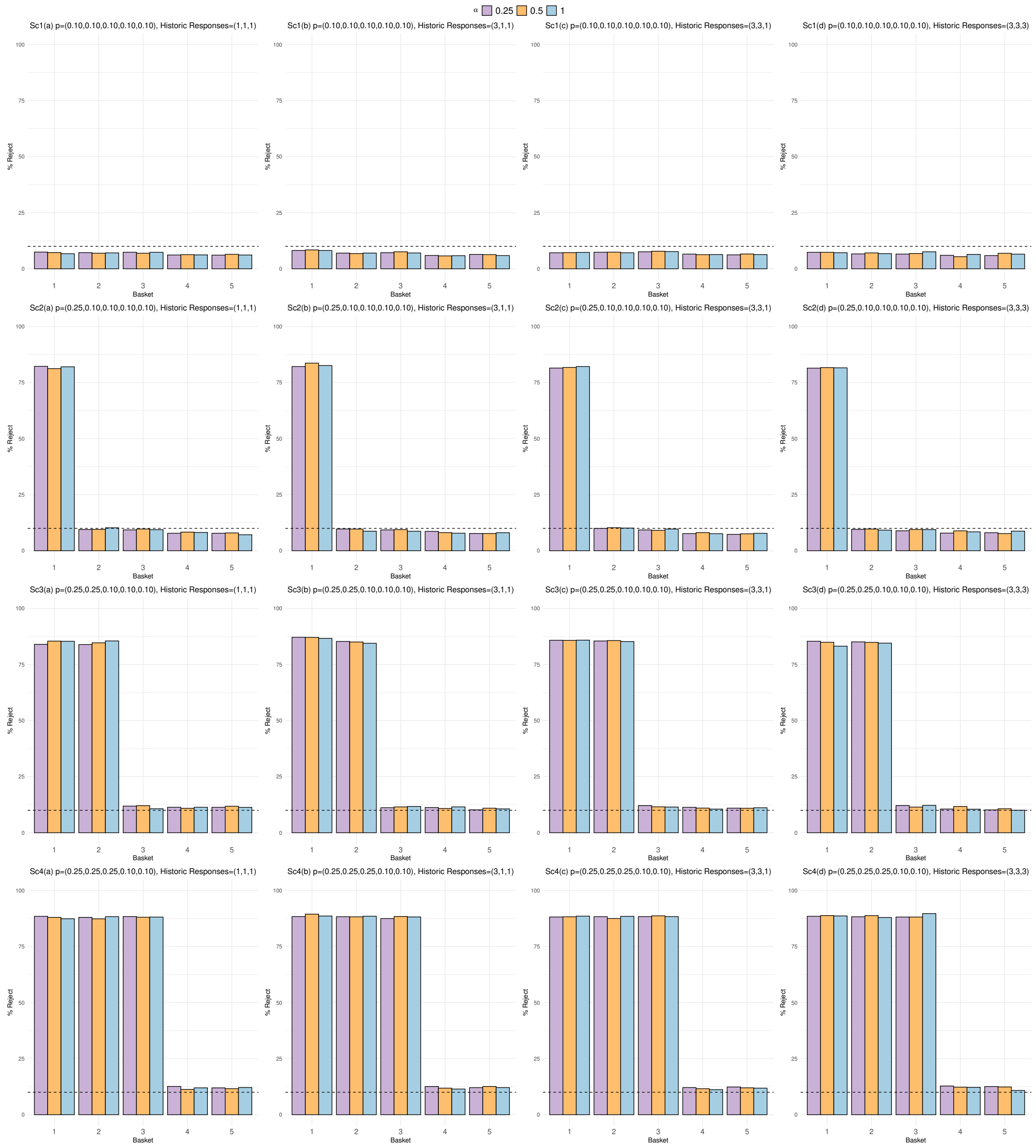

Fig. 6: The percentage of data sets where the null hypothesis were rejected per baskets under the EXppNEX model for scenarios 5-8 and 4 historic sub-cases. This is provided for three choices of α: 0.25, 0.5 and 1.

I. Sensitivity Analysis of Weights in the MLMixture Model

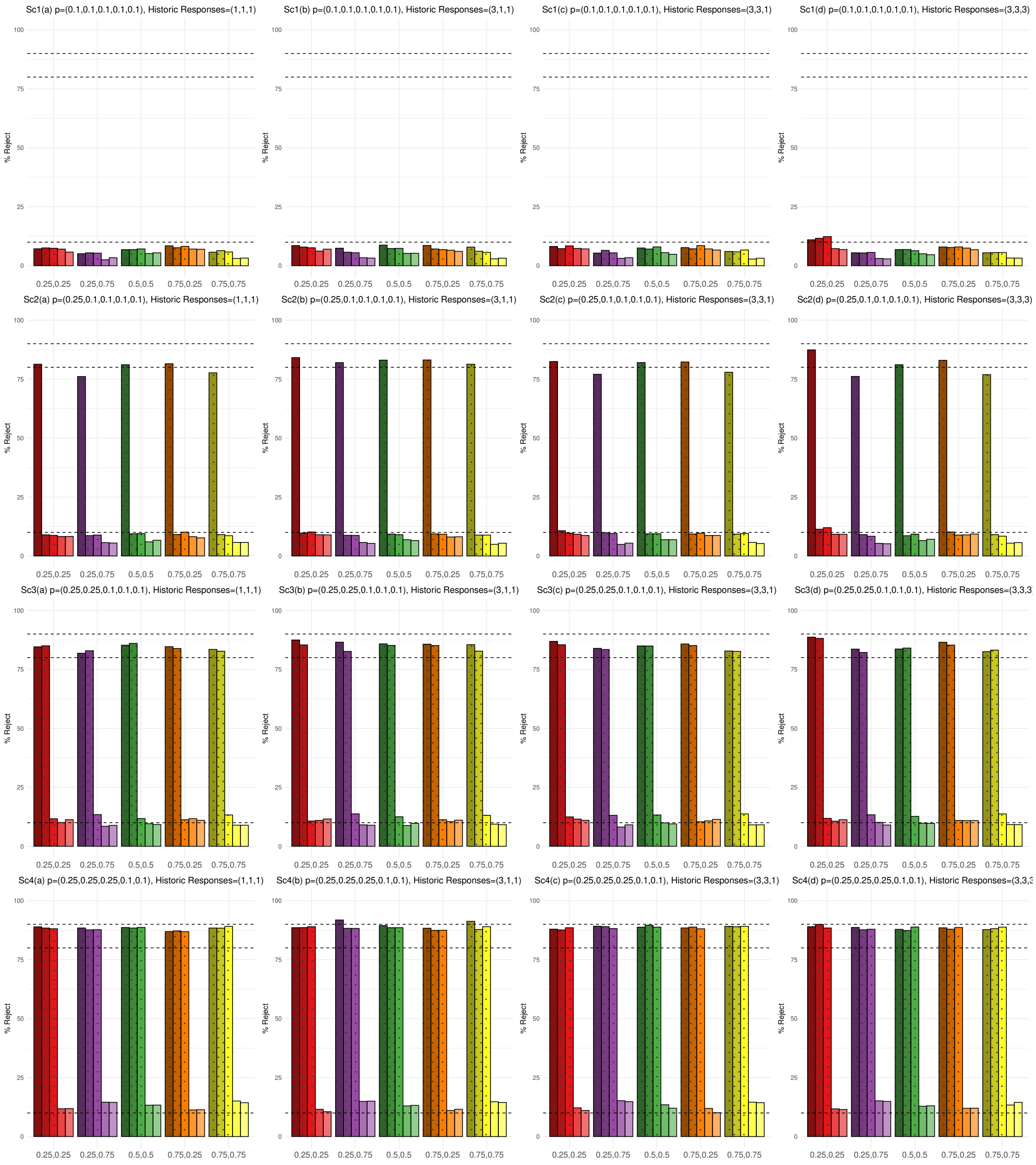

Fig. 7: The percentage of data sets where the null hypothesis were rejected per basket under the MLMixture model for scenarios 1-4 and 4 historic sub-cases. This is provided for several choices of $\pi_{\lambda,k}$ and $\pi_{\text{curr},i} = \pi_{\text{all},i}$. Each set of bars labelled x, y correspond to a setting of MLMixture weights where x is the value of $\pi_{\lambda,k}$ (set at 0.25, 0.5 or 0.75) and y are the values of $\pi_{\text{curr},i}$ and $\pi_{\text{all},i}$ which are set as equal and to either 0.25, 0.5 or 0.75.

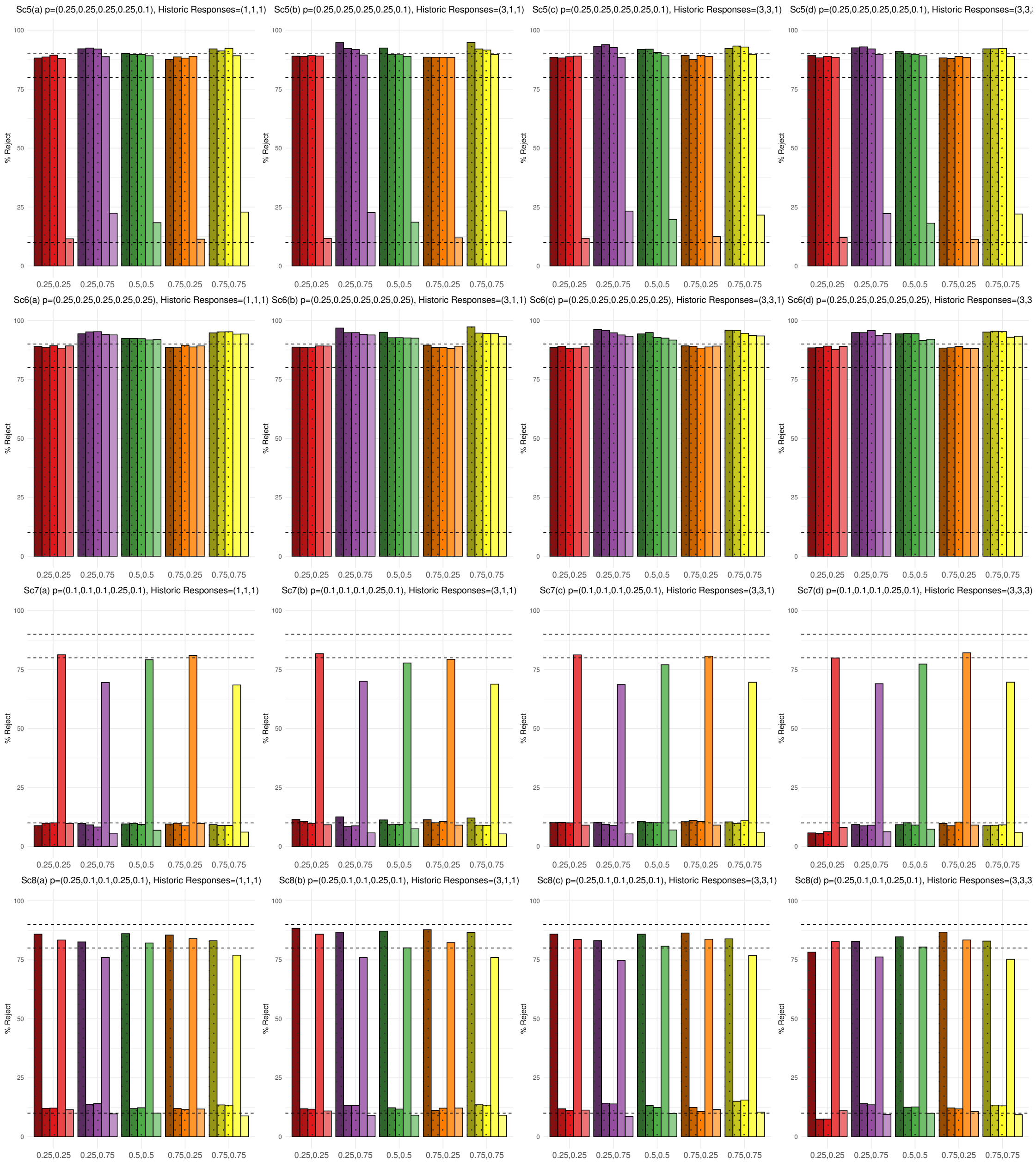

Fig. 8: The percentage of data sets where the null hypothesis were rejected per basket under the MLMixture model for scenarios 5-8 and 4 historic sub-cases. This is provided for several choices of $\pi_{\lambda,k}$ and $\pi_{\mathrm{curr},i} = \pi_{\mathrm{all},i}$. Each set of bars labelled x, y correspond to a setting of MLMixture weights where x is the value of $\pi_{\lambda,k}$ (set at 0.25, 0.5 or 0.75) and y are the values of $\pi_{\mathrm{curr},i}$ and $\pi_{\mathrm{all},i}$ which are set as equal and to either 0.25, 0.5 or 0.75.

J. Sensitivity Analysis of ρ_k in the EXNEX Model

In the EXNEX model, the ρ_k parameter is used to define the parameters in the NEX component $\theta_{2k} \sim \mathrm{N}(m_k, \nu_k)$, such that:

$$m_k = \log\left(\frac{1}{1-\rho_k}\right), \quad \nu_k = \frac{1}{\rho_k} + \frac{1}{1-\rho_k}.$$

This parameter is also implemented in the $\mathrm{EXNEX}_{\mathrm{pool}}$, $\mathrm{mEXNEX}_{\mathrm{hist}}$ and EXppNEX approach. We now conduct a sensitivity analysis on ρ_k in the EXNEX model by implementing all 8 scenarios under $\rho_k = 0.1, 0.2, \ldots, 0.9$. Table 21 presents m_k and ν_k under the various ρ_k values, whilst Figure 9 presents the mean type I error and power for all ρ_k values.

Table 21. *NEX parameters under various ρ_k values.*

ρ_k	0.1	0.2	0.3	0.4	0.5	0.6	0.7	0.8	0.9
m_k	-2.20	-1.39	-0.85	-0.41	0.00	0.41	0.85	1.39	2.20
ν_k	11.11	6.25	4.76	4.17	4.00	4.17	4.76	6.25	11.11

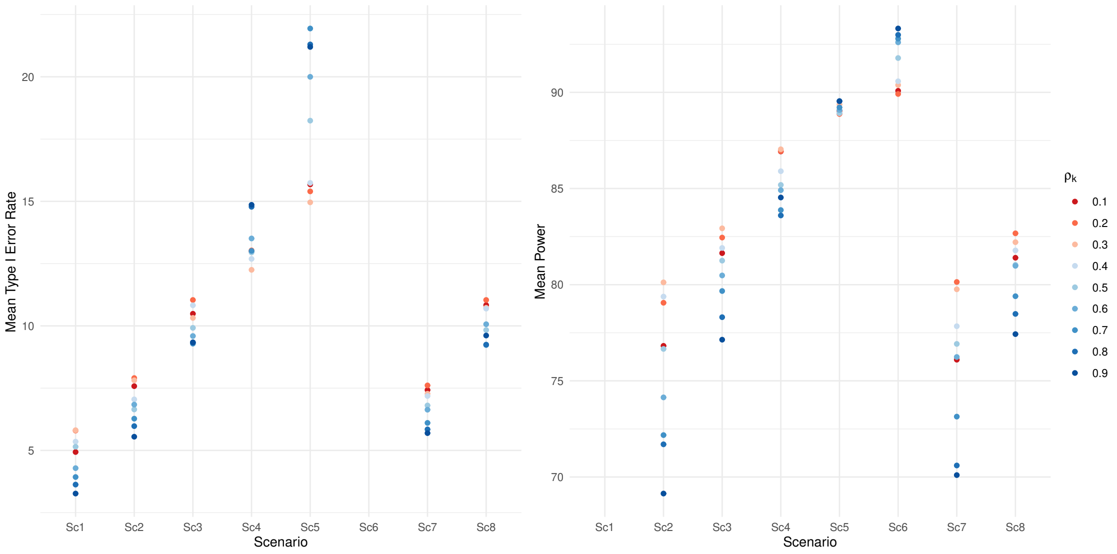

Fig. 9: Mean power and type I error rate under the EXNEX model where ρ_k (the parameter for defining the NEX parameters) varies from 0.1 up to 0.9.

It is clear that the operating characteristics are sensitive to the choice of ρ_k. In Figure 9, $\rho_k = 0.1,\ 0.2$ and 0.3, which lie between and around the null and target responses rates (0.1 and

0.25), are highlighted in red as our suggestion was to select ρ_k to fall between the null and target response rates. The mean type I error differs by less than 0.9% across these small values of ρ_k. Power is similar across some scenarios, however under scenarios 2 and 7 power differs by up to 4% between $\rho_k = 0.1$ and $\rho_k = 0.2$. This reflects the differing NEX parameters, with $\rho_k = 0.2$ giving a variance of 6.3 compared to 11.1 under $\rho_k = 0.1$. This increased precision is reflected by the increased power.

As ρ_k increases above the target response rate, the NEX distribution is centred much higher which in turn reduces power and type I error rate in all but scenarios 4 and 5. This is because the distribution of the NEX component conflicts the observed response data, thus the posterior weight on the EX component increases encouraging more borrowing, which in cases of heterogeneity such as scenarios 2 and 7, pulls the posterior probabilities down resulting in lower power and type I error. In scenarios 5 and 6 where baskets are fairly homogeneous, borrowing is appropriate, thus the increased weight on the EX component results in higher power for higher ρ_k values.

Thus in summary, selecting ρ_k between the null and target response rate is appropriate as it potentially reduces the conflict between the distribution of the NEX component and the true response data that is likely to be observed. As ρ_k increases above the target response rate, power and type I error rate tends to decrease. This implies that our choice of $\rho_k = 0.2$ throughout the simulation study gives an appropriate balance of power improvement and type I error inflation.

K. Simulation Study with $n_k = 20$ for all current baskets, k

The simulation study in the main text set the sample size of current baskets to be $n_k = 34$ for baskets $k = 1, 2, 3, 4, 5$. This is a particularly large sample size if you compare to the motivating VE-BASKET and MYPathway trials. The large sample size could potentially down-play the benefits of borrowing from the historic information, as baskets with a smaller sample size will benefit more greatly from this additional source of information. To address this, the same simulation

study as in the main text is conducted but the sample size of current baskets reduced to $n_k = 20$ for baskets $k = 1, 2, 3, 4, 5$ (sample size of historic data is still 13 in each). Results are presented in Figures 10-13.

The comparison between methods holds the same in this study as in the study presented in the main text with a larger sample size, with performances comparable. Due to the small sample size, the nominal power of 80% is rarely achieved, and in fact, is never achieved using the standard EXNEX model and an independent analysis, both of which do not consider historical data. The EXppNEX also fails to reach this nominal level, however, do get closer. $\text{mEXNEX}_{\text{hist}}$ achieves power above 80% under scenarios 5 and 6, whilst the MLMixture exceeds it in only a handful of sub-cases for select baskets.

In fact, a stratified analysis is actually preferable in cases where only one or two baskets are effective to treatment. An independent analysis has the highest power in these cases as the pull of the posterior towards the baskets with the lower responses is not present. However, when 3, 4 or 5 baskets have an effective response rate, the power is substantially higher using information borrowing approaches than a stratified analysis. To add to this, an independent analysis results in less precise point estimates in all scenarios compared to the borrowing models.

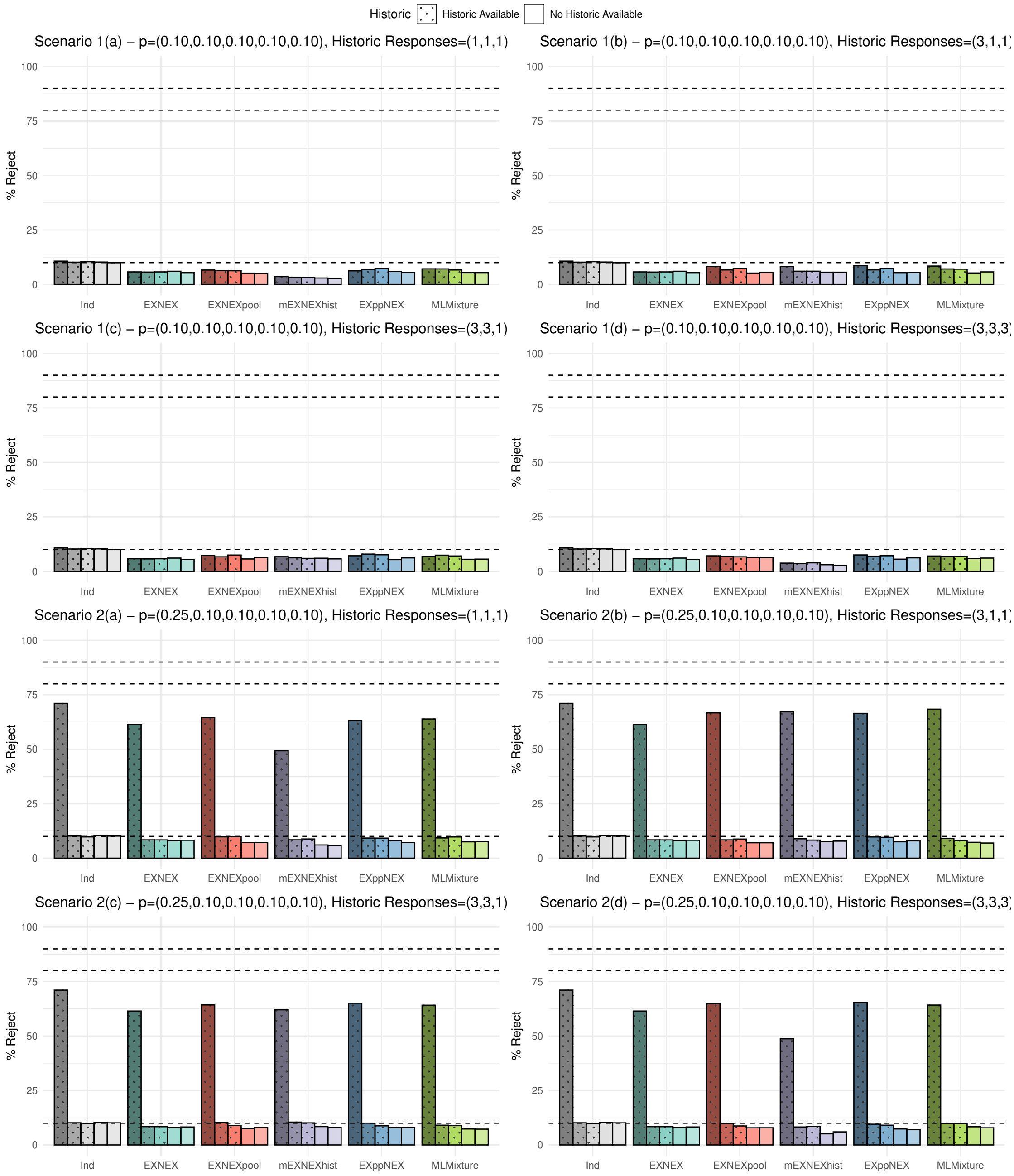

Fig. 10: Simulation results: type I error rate and power under each of the 8 approaches for scenarios 1 and 2 cases (a)-(d).

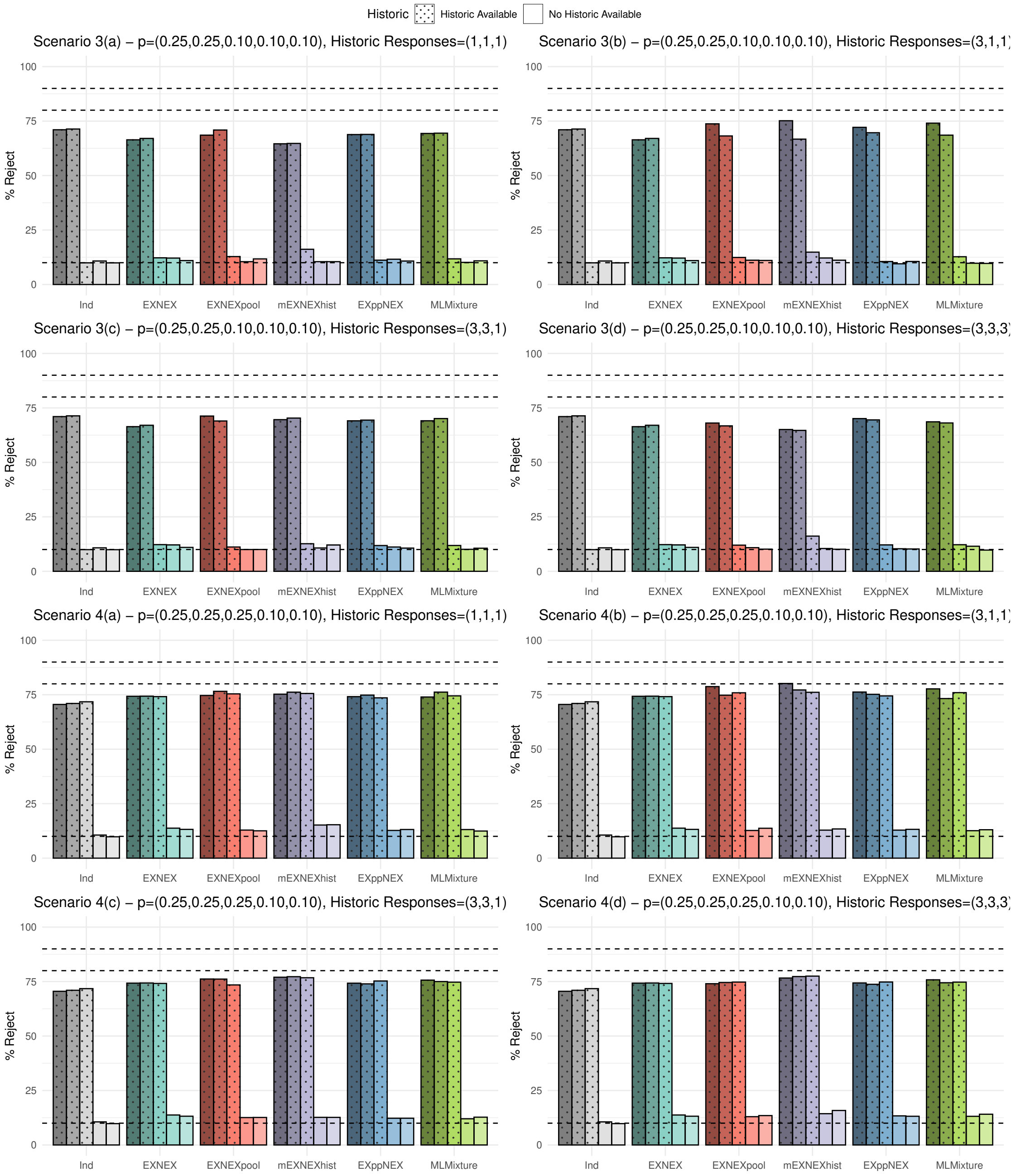

Fig. 11: Simulation results: type I error rate and power under each of the 8 approaches for scenarios 3 and 4 cases (a)-(d).

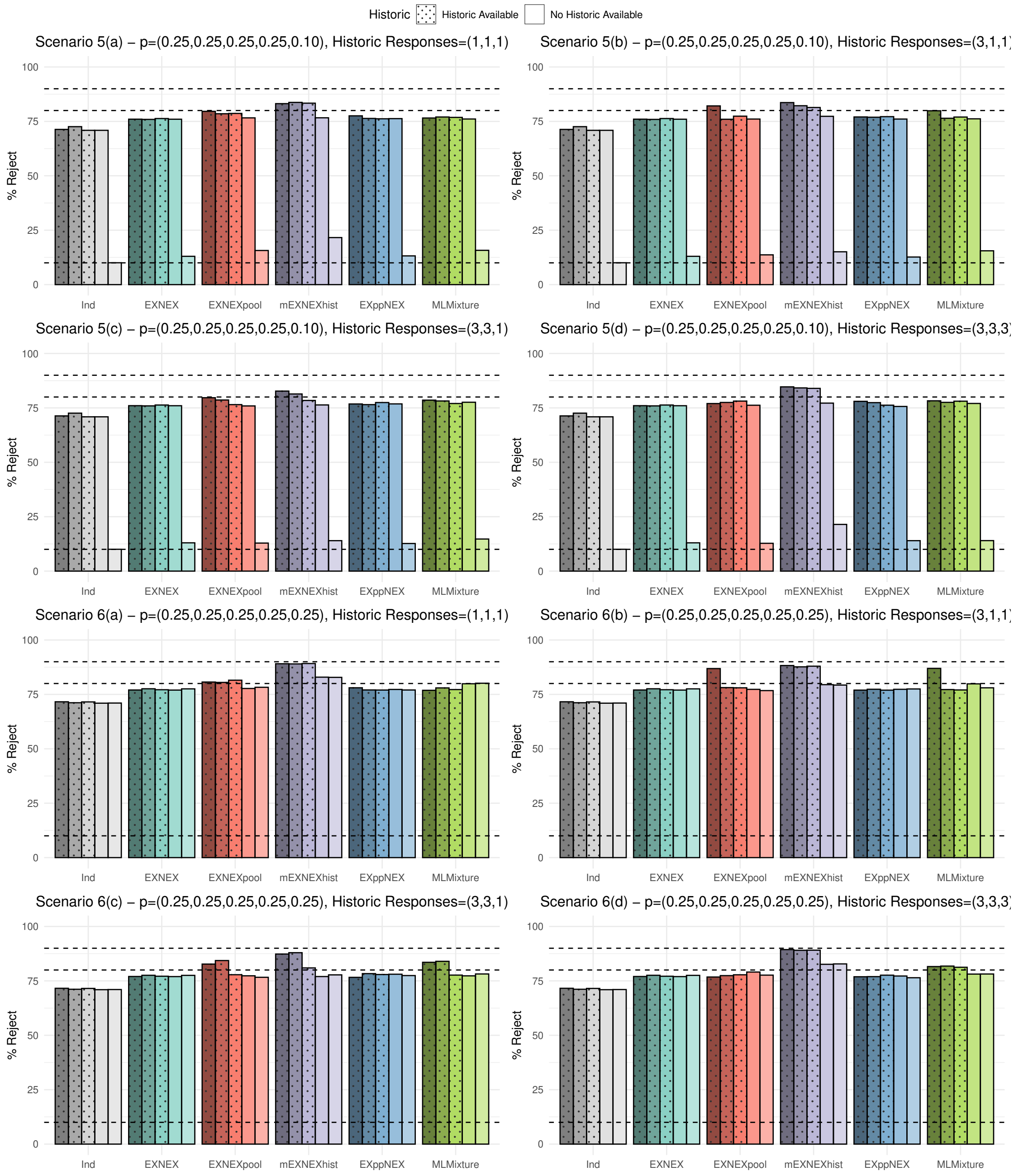

Fig. 12: Simulation results: type I error rate and power under each of the 8 approaches for scenarios 5 and 6 cases (a)-(d).

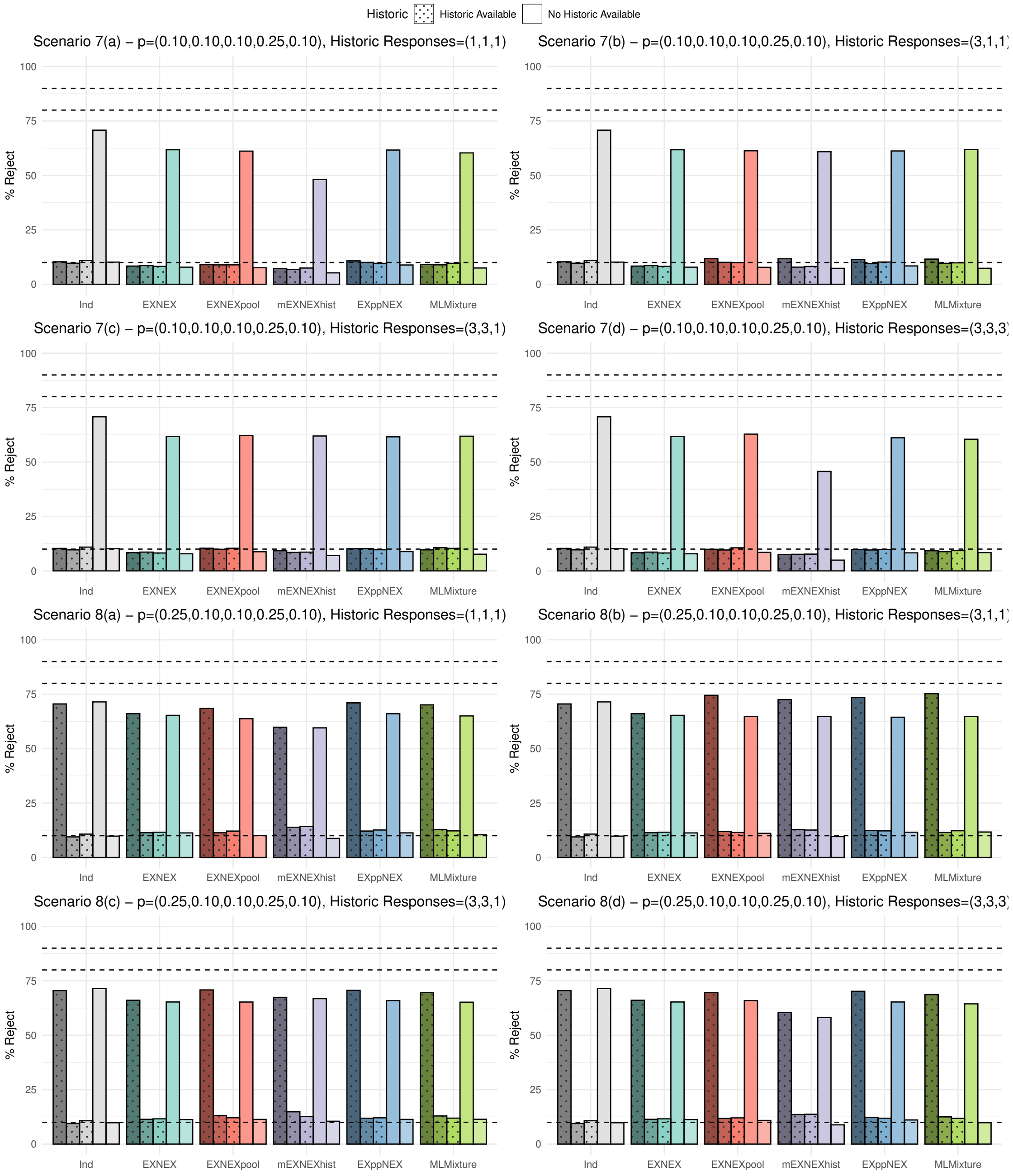

Fig. 13: Simulation results: type I error rate and power under each of the 8 approaches for scenarios 7 and 8 cases (a)-(d).

L. Unequal Sample Sizes in the Historic Information

The simulation studies presented in the main text, considered historic data of a fixed and equal sample size across baskets. This was set based on the planned sample size of the VE-BASKET trial at $n_{k^*} = 13$ for the three baskets that had historic information. However, the observed sample sizes differed from the planned values in this trial, varying from 7 to 20 patients. The observed sample sizes and total number of responses in the three historic baskets are presented in Table 22.

Table 22. *The total responses observed (y^*) and observed sample sizes (n^*) for baskets in the VE-BASKET trial. This is the set of historic data implemented in the new simulation, with two additional baskets with no historic information.*

Basket	y^*	n^*
NSCLC	8	20
Colorectal Cancer	0	10
Anaplastic Thyroid Cancer	2	7

A further simulation study is now presented where the historic data implemented is that presented in Table 22. This is done for the same 8 data scenarios presented in Table 1 in the main text. Results are presented in Figure 14. The results align with those found in the main simulation study, wherein the EXppNEX approach presents an improvement in power over the EXNEX model in all scenarios with similar type I error rate inflation. The EXppNEX has an improvement of up to a 5% in power over the EXNEX model. The first basket has the most substantial improvement in power under the EXppNEX which follows from the fact that the historic ORR is 0.4, which lies well above the target response rate of 0.25. Thus, borrowing information from the historic basket will increase the posterior probability of being greater than q_0, resulting in a higher power. This does result in an inflation in the type I error rate under scenario 7 at 12%. In contrast basket 2 has historic ORR of 0.00 and thus borrowing from it still improves the power over an EXNEX model but to a much lower degree. The MLMixture model improves power over the EXNEX model but to a lesser extent than the EXppNEX model (with

the exception of scenario 6) whilst having more substantial type I error inflation, with a 18.9% type I error rate under scenario 5.

The mEXNEXhist model performs better under this setting than the fixed sample size case presented in the main text. Using the observed historic data, the probabilities of exchangeability are set as $\pi = (0.41, 0.22, 0.50, 0.30, 0.30)$, thus favouring an independent analysis over borrowing due to the observed heterogeneity between baskets. This results in similar power to the independent analysis in scenario 2 and the highest power in scenario 8, with generally lower type I error rate than the EXNEX model throughout the scenarios. That being said, given the unfavourable operating characteristics observed in the simulation in the main text, we know that this approach is highly sensitive to the homogeneity between the historic data sources and thus is not recommended.

To summarise, the performance of the approaches under the unequal sample size setting for the historic information is akin to that under the equal sample size setting considered in the main text, with the EXppNEX model demonstrating favourable properties.

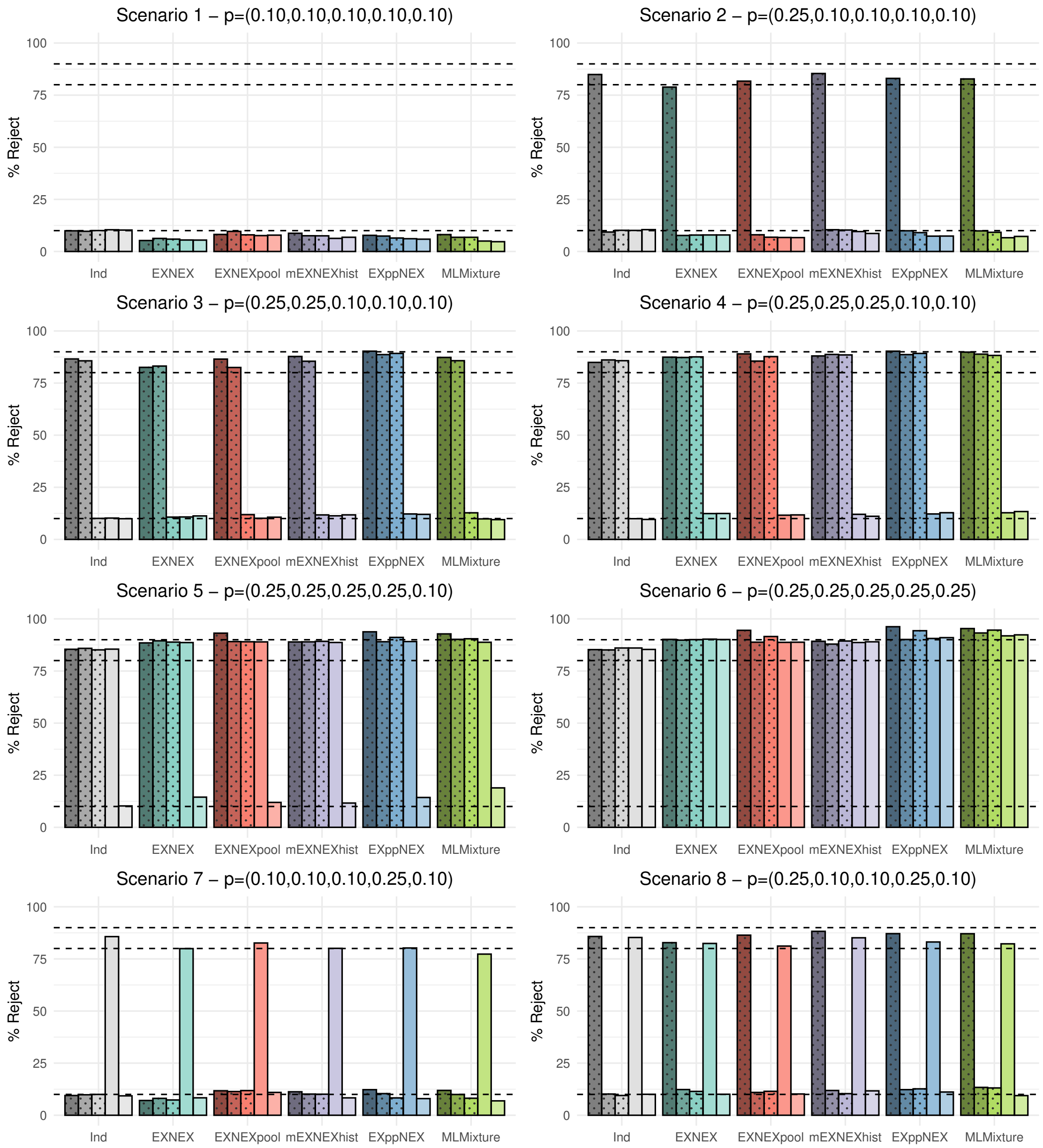

Fig. 14: Type I error rate and power under several approaches under the unequal sample size setting for the historical data.

References

Baumann, Lukas, Sauer, Lukas and Kieser, Meinhard. (2023). Basket trial designs based on power priors. *arXiv preprint arXiv:2309.06988*.

Daniells, Libby, Mozgunov, Pavel, Barnett, Helen, Bedding, Alun and Jaki, Thomas. (2024). How to add baskets to an ongoing basket trial with information borrowing. *arXiv preprint arXiv:2407.06069*.

Fuglede, Bent and Topsoe, Flemming. (2004). Jensen-shannon divergence and hilbert space embedding. In: *International symposium onInformation theory, 2004. ISIT 2004. Proceedings.*. IEEE. p. 31.

Fujikawa, Kei, Teramukai, Satoshi, Yokota, Isao and Daimon, Takashi. (2020). A bayesian basket trial design that borrows information across strata based on the similarity between the posterior distributions of the response probability. *Biometrical Journal* **62**(2), 330–338.

Schmidli, Heinz, Gsteiger, Sandro, Roychoudhury, Satrajit, O'Hagan, Anthony, Spiegelhalter, David and Neuenschwander, Beat. (2014). Robust meta-analytic-predictive priors in clinical trials with historical control information. *Biometrics* **70**.

Weber, Sebastian, Li, Yue, Seaman, John W., Kakizume, Tomoyuki and Schmidli, Heinz. (2021). Applying meta-analytic-predictive priors with the r bayesian evidence synthesis tools. *Journal of Statistical Software* **100**.

Yang, Peng, Zhao, Yuansong, Nie, Lei, Vallejo, Jonathon and Yuan, Ying. (2023). SAM: Self-adapting mixture prior to dynamically borrow information from historical data in clinical trials. *Biometrics* **79**(4), 2857–2868.
